# TRAF6 regulates the abundance of RIPK1 and inhibits the RIPK1/RIPK3/MLKL necroptosis signaling pathway and affects the progression of colorectal cancer

**DOI:** 10.1038/s41419-022-05524-y

**Published:** 2023-01-05

**Authors:** Penghang Lin, Chunlin Lin, Ruofan He, Hui Chen, Zuhong Teng, Hengxin Yao, Songyi Liu, Robert M. Hoffman, Jianxin Ye, Guangwei Zhu

**Affiliations:** 1 Department of Gastrointestinal Surgery 2 Section, Institute of Abdominal Surgery, Key Laboratory of Accurate Diagnosis and Treatment of Cancer, The First Affiliated Hospital of Fujian Medical University, Fuzhou, 350005 China; 2 Key Laboratory of Ministry of Education for Gastrointestinal Cancer, Fujian Medical University, Fuzhou, 350000 China; 3 AntiCancer, Inc., San Diego, CA USA; 4 Department of Surgery, University of California, San Diego, CA USA

**Keywords:** Colon cancer, Biomarkers

## Abstract

Colorectal cancer cannot be completely cured at present, and it is still an important clinical medical problem. TRAF6 is highly expressed in many malignant tumors. However, the role of TRAF6 in colorectal cancer is still controversial, mainly because the specific regulatory mechanism of colorectal cancer is still unclear, and the death mode of colorectal cancer cells has not been elucidated. The recent study found that TRAF6 inhibits necroptosis in colorectal cancer cells via the RIPK1/RIPK3/MLKL signaling pathway. The RIPK1 inhibitor Necrostain-1 inhibits colorectal cancer cell necroptosis via the RIPK1/RIPK3/MLKL signaling pathway. TRAF6 directly interacts with RIPK1 through the polyubiquitination of Lys48-linked RIPK1 and reduces the levels of RIPK1 protein in colorectal cancer cells, leading to necroptosis, thus promoting the proliferation of colorectal cancer cells. The recent study demonstrated that TRAF6 promotes colorectal cell progression by inhibiting the RIPK1/RIPK3/MLKL necroptosis signaling pathway, which may provide a new therapeutic target for colorectal cancer.

## Introduction

Colorectal cancer is a common malignant tumor in the digestive tract, with high morbidity and mortality, and the mortality rate ranks third among malignant tumors [1, 2]. In recent years, gene-targeted drug therapy for refractory patients can effectively improve the prognosis and survival status of colorectal cancer patients [3, 4]. The way of cell death has rapidly become the focus of research on various tumors. Among them, dysregulation of the necroptosis regulatory mechanism is important in promoting tumorigenesis and development. Tumor necrosis factor receptor-associated factor 6 (TRAF6), a member of the tumor necrosis factor receptor-associated factor family, is one of the most widely studied and important members of signal transduction [5–7]. However, its role in colorectal cancer is controversial, especially since the regulatory mechanism of colorectal cancer necroptosis has not been studied, so it is of great significance to study the in-depth regulation of TRAF6 on colorectal cancer necroptosis.

So far, people have made progress in cancer cell death, among which necroptosis is considered one of the important death ways affecting the occurrence and development of cancer [8–12]. Receptor-interacting protein kinase 1 (RIPK1) belongs to the receptor-interacting protein family (RIPs), with an N-terminal kinase domain and a C-terminal death domain, as well as a RIP homotypic interaction modify (RHIM domain); The N-terminal kinase domain is a protein with serine/threonine-protein kinase activity, which plays a key role in regulating cell death and survival [13–16]. The main mechanism is that RIPK1 binds and activates RIP3 through RHIM, which eventually induces mixed lineage kinase domain proteins like protein (MLKL). Threonine 357 and serine 358 residues of MLKL are phosphorylated, oligomerized and localized to the cell membrane [17], causing necrotizing membrane destruction and releasing DAMPs to activate inflammatory and immune responses [18]. The signaling pathway is called RIPK1-RIPK3-MLKL.

Recent studies have shown that TRAF6 has a critical role in depressive behavior in neuronal necroptosis. TRAF6 is involved in macrophages in inhibiting RIPK3-mediated hepatocyte necroptosis after co-culture with primary hepatocytes [19]. Studies have shown that TRAF6 is highly expressed in malignant tumors. It can enhance the invasiveness and metastasis of cancer cells through Ras kinase, and can also affect cell migration, and invasion and inhibit apoptosis by regulating the NFκB-CD24/CXCR4 signaling pathway [20]. Yao et al. found that knocking out the TRAF6 gene could prevent the metastasis of esophageal squamous cell carcinoma cells in vivo or in vitro, studied the mechanism of metastasis, and enhanced that it was the result of ERK phosphorylation caused by the binding of the N-terminal of TRAF6 to Ras. Starczynowski et al. found that TRAF6 was relatively up-regulated at the gene level in both non-small cell lung cancer and small cell lung cancer, and overexpressed TRAF6 triggered Ras-dependent tumorigenesis. In addition, overexpression of TRAF6 can not only inhibit apoptosis of various types of cancer cells but also promote cancer cell proliferation [21, 22]. Sun et al. [6] found that when siRNA was used to silence the TRAF6 gene in human colon cancer cells, the cell proliferation was slowed down, and the expression of CyclinD1 in vitro and in vivo was decreased. It was inferred that TRAF6 may help colon cancer cells to proliferate by participating in cell division. This is consistent with the results of our study. However, Wu et al. [23] found that TRAF6 inhibits colorectal cancer metastasis by regulating selective autophagy CTNNB1/β-catenin degradation, which is controversial. Although there are many studies on the effect of TRAF6 on tumors, the research on the signal regulation of TRAF6 is not thorough enough. The effect of TRAF6 in colorectal cancer is still controversial, and more research investment is needed to affect the way of tumor cell death. Therefore, our team further studied the role of TRAF6 in colorectal cancer, clarified its growth in colorectal tumors, and was the first to discover that it can increase its malignant biological behavior by regulating the way of cell death.

The present study reports the detailed mechanism by which TRAF6 regulates necroptosis in colorectal cancer cells. We found that TRAF6 may degrade RIPK1 through the Lys48(K48) Ub-linked pathway, resulting in a decrease in the levels of RIPK1 protein, and a decrease in the levels of linked downstream target proteins RIPK3 and MLKL-linked proteins. Thus, the necroptosis of colorectal cancer cells is inhibited and the progression of colorectal cancer cells is promoted.

## Materials and methods

### Cell culture and transfection

SW480 and HCT116 human colon cancer cell lines were obtained from the Chinese Academy of Sciences (Shanghai) Cell Bank. Both cell lines were cultured in DMEM (GIBCO) containing 10% fetal bovine serum (FBS), 100 μg/ml streptomycin, and 100 IU of penicillin (GIBCO) in a 5% CO_2_ incubator. MC38 mouse colon cancer cells were obtained from the Chinese Academy of Sciences (Shanghai) Cell Bank and cultured in 1640 (GIBCO) containing 10% FBS, 100 μg/ml streptomycin, and 100 IU of penicillin (GIBCO) in a 5% CO_2_ incubator. The HEK293T cell line was obtained from the Key Laboratory of Gastrointestinal Oncology, Ministry of Education, Fujian Medical University, and was cultured in DMEM containing 10% FBS, 100 μg/ml streptomycin, and 100 IU of penicillin (GIBCO) in a humidified atmosphere of 5% CO_2_. Various gene primers and cloning vectors were synthesized by Shangya (shanghai) Biosynthesis. Small interfering RNA targeting TRAF6 was synthesized by Zimmer Bio (Shanghai). TARF6 knockout was performed using the CRISPR-Cas9 gene knockout system (GeneCopoeia) according to the manufacturer’s instructions to generate stable TRAF6 knockout cell lines. DNA transfection was accomplished in 6-well or 10 cm plates using Lipoect Tamine™ 3000 reagent (Invitrogen, Thermo Fisher Science) according to the manufacturer’s instructions.

### Mice, reagents, and antibodies

Mice with the C57BL/6 genetic background of RIPK3^−/−^ and MLKL^−/−^ were a gift from Dr. Jiahuai Han’s laboratory, School of Life Sciences, Xiamen University. Genotypes were determined by PCR amplification of the shear tails. Mice were housed in a specific pathogen-free facility on a 12-h light/dark cycle. BALA/c nude mice were purchased from Shanghai Laboratory Animal Center, Chinese Academy of Sciences. All experiments were performed by the Chinese Guidelines for the Care and Use of Laboratory Animals and were approved by the Laboratory Animal Management and Ethics Committee of Fujian Medical University. Antibodies specific for HA-tag (5017S), Myc-tag (2276S), Flag-tag (14793S), GAPDH (5174S), TARF6 (8028S), RIPK1 (73271S), RIPK3 (10188S) and MLKL (37705S) were obtained from Cell signaling technology. Specific phosphorylation site antibodies against Phospho-RIP (Ser166) (44590S), Phospho-RIP3 (Thr231/Ser232) (91702S), Phospho-MLKL (Ser345) (37333S) were also purchased from Cell signaling technology. Annexin V-FITC kit (APOAF) and inhibitor Necrostatin-1(Nec-1) (480066) were purchased from Sigma-Aldrich. Cycloheximide (CHX), MG132 and Necrostatin-1s(Nec-1s) were purchased from MedChemExpress. Other reagents not listed were from Sigma-Aldrich and Thermo Fisher Scientific.

### Co-immunoprecipitation, polyubiquitination, and western blot assay

The protein extraction procedure was performed on ice, and cell samples were lysed with IP lysis buffer (Thermo Fisher Scientific) supplemented with protease inhibitor cocktail (Beyotime) for 30 min on ice, followed by centrifugation at 12,000 × *g* for 10 min. The supernatant was collected and the total protein concentration was measured. By using a BCA kit (Thermo Fisher Scientific). For immunoprecipitation, ubiquitinated proteins were immunoprecipitated with HEK293T, HCT116, or SW480 cells transiently overexpressing Flag-TRAF6 or the Flag-TRAF6 fragment and Myc-RIPK1, and equal amounts of lysates were mixed with IgG, anti-antibody DYKDDDDK affinity beads or anti-Myc affinity beads (Pierce) conjugates were incubated overnight at 4 °C. Then, the beads were washed three times with IP Lysis buffer and ready for western blotting. For polyubiquitination experiments, immunoprecipitation of ubiquitinated proteins was performed with HEK293T, HCT116, or SW480 cells transiently overexpressing Flag-TRAF6 or the Flag-TRAF6 fragments and HA-Ub or K48-Ub, using equal amounts of the lysates were added with anti-IgG, anti-Myc affinity beads (Pierce) conjugate and incubated overnight at 4 °C. Then, the beads were washed three times with IP Lysis buffer and ready for western blotting with HA-Ub. Immunoblotting experiments were performed as described above [24].

### Microscopy

For light microscopy capture of HCT116 and SW480 cells, cells were seeded in 6-well plates at 40–60% confluency. The morphology of necroptosis cells was captured as phase-contrast images with a Nikon microscope under different treatment conditions.

For confocal microscopy, HEK293T, HCT116, and SW480 cells were washed with PBS and then fixed in 4% paraformaldehyde. Cells were blocked with blocking buffer (5% BSA and 0.2% Triton X-100) and then incubated with the appropriate primary antibody overnight at 4 °C. After washing with wash buffer PBS, cells were incubated with FITC-conjugated secondary antibodies (Life Technologies) for 1 h at 37 °C in the dark. To indicate nuclei, cells were stained with 4′,6-diamidino-2-phenylindole (DAPI, ab285390) for 5 min. Images were captured under a laser scanning confocal microscope TCS SP8.

For the detection of cell necroptosis, an Annexin V-FITC kit was used for observation. The cells were seeded in 24-well plates at a confluence of 40–60%, and the cells were treated according to the experimental design. The culture medium was removed, and PBS was used to wash cells 2–3 times, add appropriate premix working solution (Hoechst 33342 live cell staining solution is added together to indicate cell nuclei), and incubate at 37 °C for 10–20/min in the dark, in which PI staining-positive necrotic cells are red fluorescence, and nuclei staining is blue fluorescence. The fluorescence of experimental cells in each group was observed under an inverted fluorescence microscope for image capture. Note that the entire process needs to be protected from light.

The ultrastructural changes of necroptosis cells were observed under an electron microscope. The cells of each experimental group were seeded in 10 cm plates at 40–60% confluence, and the cells were treated according to the experimental design, the culture medium was removed, washed 2–3 times with PBS, and fixed with electron microscope fixative. Samples were processed by professional technicians and images were acquired using an electron microscope.

### Immunohistochemical assay

Immunohistochemical experiments were performed by ElivisionTM plus Polymer HP (mouse/rabbit) IHC kit (MXB Biotechnologies). Tissue sections were obtained from formalin-fixed and paraffin-embedded tissue specimens. The detailed method is carried out according to the previous method [25]. The serial sections of colorectal cancer tissue used a 1:500 dilution of rabbit monoclonal TRAF6, RIPK1, RIPK3, and MLKL antibodies. The levels of tissue proteins were assessed using Image-Pro Plus 6.0 software (Media Cybernatics, Inc). Three slices were randomly selected from each group, five fields of the same size were randomly selected from each slice, and their cumulative integrated optical densities were measured.

### Cell proliferation and colony-forming assay

Transiently transfected and stably expressed cells of each group, a total of 1 × 10^3^ HCT116 cells and 2 × 10^3^ SW480 cells, were seeded into 96-well plates, respectively. Each cell has five parallel experimental wells. For the next 5 days, cell proliferation was measured by Cell Counting Kit-8 (Dojindo, Japan). A total of 10 μl of CCK8 solution was added per well and after 2 h incubation in an incubator (Thermo Fisher Scientific), absorbance readings were measured by Gen5 (BioTek) at 450 nm. Each experiment was repeated three times independently.

Transiently transfected and stably expressed cells of each group, a total of 600 HCT116 and SW480 cells, were seeded into six-well plates, respectively. HCT116 and SW480 cells were cultured in an incubator for 9 days or 14 days, respectively. Washed 2–3 times with PBS, fixed with methanol, and stained with 1% crystal violet (Beyotime Biotechnology) to obtain clonal colony-forming units, rinsed with gentle water flow. The number of clonal colony-forming units was calculated by ImageJ (National Institutes of Health). Each experiment was repeated three times independently.

### Mouse experiments

All animal experiments were carried out by the “Guidelines for the Care and Use of Laboratory Animals of the National Institutes of Health”, and animal experiments were carried out in the Animal Experiment Center of Fujian Medical University and approved by the University Ethics Committee. All experimental BALB/c nude mice were female and ~5 weeks old. A subcutaneous tumor model of BALB/c female nude mice with high expression of TRAF6 cells was established, and HCT116 cells 5 × 10^6^ with stable high expression of TRAF6 were inoculated in subcutaneously the nude mice of the experimental group. The nude mice of the control group were injected with the same amount of cells with a mock plasmid. BALB/c female nude mice were subcutaneously injected KO-TRAF6 cells xenograft model, and HCT-116 cells 5 × 10^6^ with KO-TRAF6 by CRISPR/Cas9 gene were subcutaneously inoculated in nude mice of the experimental group. The control group was injected with the same amount of mock control cells. Nec-1 interfered experimental mice were injected subcutaneously with Nec-1 at the recommended dose of 1.65 mg/Kg of Nec-1 every 3 days from the 3rd day, and the control group was injected with the same amount of DMSO for a total of 7 times. All mice used for experiments on the C57BL/6 genetic background were male, ~5 weeks old. To construct a subcutaneous high expression TRAF6 cell transplantation tumor model on the RIPK3^−/−^ or MLKL^−/−^ C57BL/6 genetic background. In total, 8 × 10^6^ MC38 cells stably overexpressing TRAF6 were inoculated subcutaneously into experimental mice, and control mice were injected with equal amounts of cells stably expressing mock plasmids. Knockout mice were all identified by the mouse tail gene.

All mice meeting the required tumor model of weekly age were randomly divided into four or two groups as needed. Mice were palpated through the abdomen and tumors were measured every 3 days. About 3 weeks later, the nude mice were sacrificed. Subcutaneous xenograft were excised, photographed, and weighed and measured immediately. The measurement formula of tumor volume was length × width × height × *π*/6. Transplanted tumors were soaked in formalin for immunohistochemical analysis.

### Statistical analysis

All statistical analyses were performed using GraphPad Prism 9.0 software, such as *t*-test or one-way ANOVA analysis. All experiments were repeated at least three times and (*n* = 3) for each experimental group. Data are presented as mean ± SD. *p* < 0.05 was considered statistically significant.

## Result

### High expression of TRAF6 in colorectal cancer cells can inhibit necroptosis

**Fig. 1:**
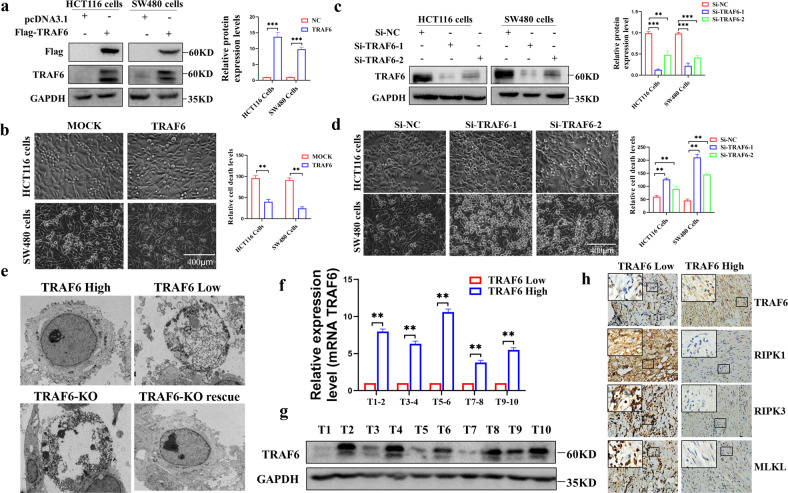
Differential expression of TRAF6 protein affects the death of colorectal cancer cells. **a** In transient transfection experiment with HCT116 and SW480 cells, compared with a mock control plasmid, the levels of TRAF6 protein in colorectal cancer cells in the TRAF6 overexpression group was significantly changed. **b** Overexpression of TRAF6 cell state in HCT116 and SW480 cells was significantly changed under light microscopy (×200). **c** In HCT116 and SW480 cells, transient transfection of knockdown TRAF6 group colorectal cancer cells showed significant alterations in TRAF6 protein expression levels. **d** In HCT116 and SW480 cells, the cellular state of knockdown TRAF6 was altered under light microscopy (×200). **e** Ultrastructural differences of colorectal cancer cells observe electron microscope (×2000) with differentially-expressed TRAF6 protein. **f**, **g** Detecting the TRAF6 mRNA and protein levels in ten clinical tissue samples, they were sorted into the TRAF6 Low and TRAF6 High groups by the median number of TRAF6 protein levels. Random matching was performed to obtain the mRNA (**f**) and protein-level (**g**) results. **h** Serial immunohistochemical sections of tissue specimens from colorectal cancer patients with differential TRAF6 protein levels, images were obtained with an inverted microscope (×400). (*p < 0.05, **p < 0.01, ***p < 0.001, with an unpaired Student’s *t* test (**a**, **b**, **f**) or one-way ANOVA analysis (**c**, **d**)).

Since our previous experiments revealed that the difference in TRAF6 protein levels in HCT116 and SW480 cells could affect the cell death situation. We validated the initial TRAF6 expression levels (Fig. S1a) in some colorectal cancer cell lines, including HCT116 cells and SW480 cell lines. We detected the death of colorectal cancer cells by overexpressing TRAF6 (Fig. 1a) and knocking down TRAF6 (Fig. 1c) protein expression levels and the results showed that high expression of TRAF6 could significantly inhibit the death of colorectal cancer cells (Fig. 1b, d). We showed by electron microscopy that colorectal cancer cells with low expression can show significant necroptosis compared with high expression of TRAF6 cells. KO-TRAF6 colorectal cancer cells showed better improvement in necroptosis, and we showed representative pictures of necroptosis at different stages (Fig. 1e), which demonstrated that TRAF6 protein expression can inhibit cell necrosis. To further investigate this phenomenon, we examined TRAF6 mRNA and protein levels in clinical tissue specimens from ten colorectal cancer patients (Table S1) and divided them into TRAF6 Low and TRAF6 High groups in order of median TRAF6 protein levels. Random matches were performed to show the mRNA (Fig. 1f) and protein levels (Fig. 1g) results. Serial paraffin cuts of tissue specimens from colorectal cancer patients with differential TRAF6 protein levels were subjected to immunohistochemical assays for the RIPK1 classical necroptotic pathway RIPK1-RIPK3-MLKL, and the protein levels of RIPK1, RIPK3, MLKL in human colorectal cancer tissues from the high level TRAF6 group was found to be significantly lower than that of the low expression TRAF6 group (Fig. 1h). We searched for colorectal cancer patient data from the GEO database and plotted TRAF6 expression-related survival curves (Fig. S1b), and the analysis showed that patients with high TRAF6 expression had a poorer survival prognosis. Therefore, we can infer that TRAF6 inhibits the necroptosis of colorectal cancer cells by inhibiting the RIPK1-RIPK3-MLKL signaling pathway.

### There is a direct interaction between TRAF6 and RIPK1 protein

**Fig. 2:**
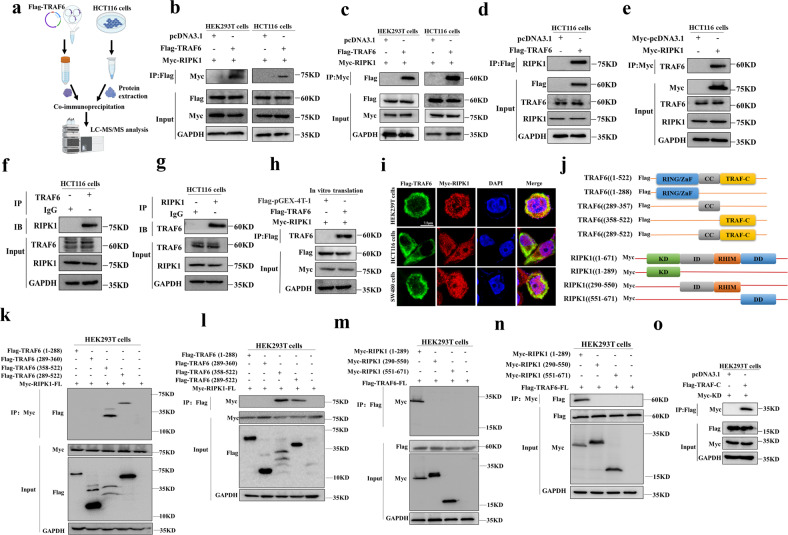
The interaction between TRAF6 and RIPK1 protein. **a** Schematic diagram of extracting and purifying TRAF6 protein and HCT116 cells protein by in vitro transcription and translation system for co-immunoprecipitation, with analysis of the precipitated product with LC-MS/MS. **b**, **c** HEK293T and HCT116 cells were co-transfected with Flag-TRAF6 with Myc-RIPK1, and co-immunoprecipitation between exogenous TRAF6 and RIPK1 protein was detected with anti-Myc antibodies (**b**) and anti-Flag antibodies (**c**). **d**, **e** Anti-Flag (**d**) and anti-Myc (**e**) affinity beads were used to detect co-immunoprecipitation between exogenous TRAF6 and endogenous RIPK1 protein in HCT116 cells co-transfected with Flag-TRAF6 and mock control plasmids. **f**, **g** Co-immunoprecipitation of endogenous TRAF6 and RIPK1 proteins were detected with anti-IgG and anti-TRAF6 (**f**) or anti-RIPK1 (**g**) affinity beads, respectively. **h** Co-immunoprecipitation of Flag-TRAF6 and Myc-RIPK1 proteins was directly obtained in an in vitro transcription and translation system. **i** The protein levels of Flag-TRAF6 and Myc-RIPK1 proteins in HEK293T, HCT116, and SW480 cells were detected by confocal microscopy. **j** Schematic diagram of the TRAF6 and RIPK1 domains, showing the full-length and segmented domains of the TRAF6 and RIPK1 proteins. **k**, **l** The protein domain fragment of Flag-TRAF6 was co-transfected with the full-length Myc-RIPK1 protein in HEK293 cells, and the TRAF6 protein domain fragment was detected with anti-Myc (**k**) and anti-Flag (**l**) affinity beads co-immunoprecipitation with RIPK1 protein. **m**, **n** HEK293T cells were co-transfected with Flag-TRAF6 and Myc-RIPK1 protein domain fragments, and the interaction between TRAF6 and RIPK1 protein domain fragments was detected with anti-Flag (**m**) and anti-Myc (**n**) affinity beads by co-immunoprecipitation. **o** HEK293T cells co-transfected with Flag-TRAF-C and Myc-KD protein domain, anti-Flag affinity beads were used to detect the co-immunoprecipitation between the two fragment domains.

To explore the detailed relationship between TRAF6 and RIPK1 protein, the purified TRAF6 protein, and HCT116 cell total protein was subjected to co-immunoprecipitation, and the precipitated product was analyzed by LC-MS/MS (Fig. 2a). We further determined whether TRAF6 interacts with RIPK1 protein by co-immunoprecipitation with exogenous TRAF6 and RIPK1 proteins (Fig. S1c). Our experimental results indicated a direct interaction between exogenous TRAF6 and RIPK1 proteins (Fig. 2b, c). We then confirmed the interaction between exogenous TRAF6 and endogenous RIPK1 protein (Fig. 2d), as well as the interaction between endogenous TRAF6 and exogenous RIPK1 protein (Fig. 2e). In addition, we also validated the co-immunoprecipitation of endogenous TRAF6 and RIPK1 proteins to confirm the direct interaction between TRAF6 and RIPK1 protein (Fig. 2f, g). And the TRAF6 and RIPK1 proteins were obtained by transcription and translation in vitro, again indicating that TRAF6 interacts with RIPK1 proteins in vitro (Fig. 2h). By confocal microscopy, we could obtain the interaction between TRAF6 and RIPK1 protein in HEK293T, HCT116, and SW480 cells (Fig. 2i).

To further study the molecular basis of the direct interaction between TRAF6 and RIPK1 protein, we checked through NCBI and Genecard, designed a series of segmented functional domains of TRAF6 and RIPK1 protein, and drew their schematic diagrams (Fig. 2j) to further study its mechanism of action. By co-immunoprecipitating each domain of TRAF6 protein with RIPK1 protein in forward and reverse directions (Fig. 2k, l), our data indicated that the TRAF-C domain in TRAF6 protein is a key domain that directly interacts with RIPK1 protein. Similarly, each domain of RIPK1 protein was co-immunoprecipitated with TRAF6 protein (Fig. 2m, n), and the results showed that the KD domain in RIPK1 protein is the key structure that directly interacts with TRAF6 protein. Therefore, we performed co-immunoprecipitation validation of the TRAF-C domain with the KD domain (Fig. 2o), which indicated that TRAF6 is regulated by the direct interaction of the TRAF-C domain with the KD domain protein in RIPK1.

### TRAF6 can reduce the RIPK1 protein levels

**Fig. 3:**
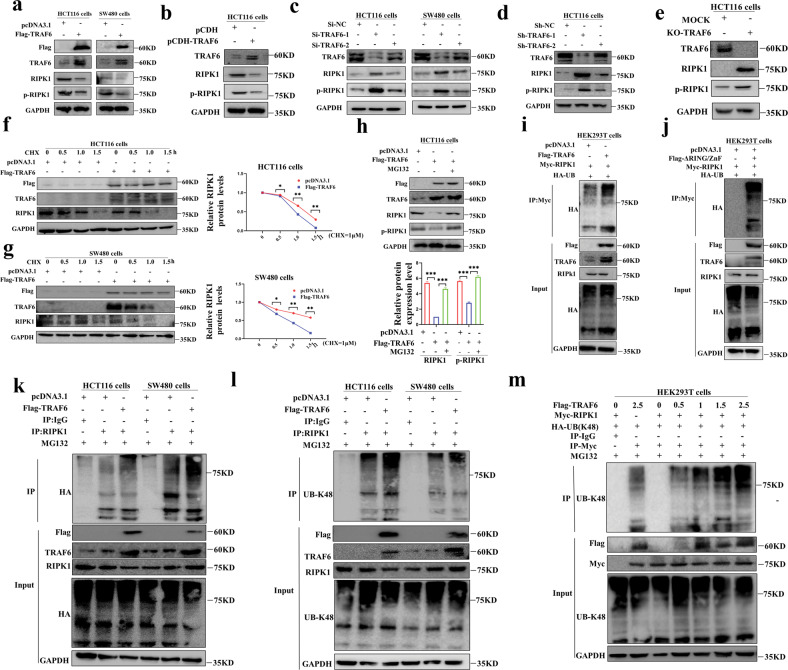
TRAF6 degrades RIPK1 protein through direct K48-linked polyubiquitination. **a** RIPK1 and p-RIPK1 proteins were detected in western blotting from HCT116 and SW480 cells transiently transfected with Flag-TRAF6 or mock control plasmid. **b** HCT116 cells were stably transfected with Flag-TRAF6 or mock control plasmid. Protein levels of RIPK1 and p-RIPK1 were detected in western blotting. **c** HCT116 and SW480 cells were transiently transfected with Si-NC, Si-TRAF6-1, or Si-TRAF6-1 plasmids. Protein levels of RIPK1 and p-RIPK1 were detected in western blotting. **d** HCT116 cells were stably transfected with Sh-NC, Sh-TRAF6-1, or Sh-TRAF6-1 plasmids, and the protein levels of RIPK1 and p-RIPK1 were detected in western blotting. **e** The proteins from HCT116 cells and control cells in which TRAF6 was knocked out by the CRISPR/Cas9 gene were extracted. Protein levels of RIPK1 and p-RIPK1 were detected in western blotting. **f**, **g** HCT116 (**f**) and SW480 (**g**) cells were transiently transfected with Flag-TRAF6 or mock control plasmid and cultured for 24 h, then incubated with CHX (20 μM) for 0, 0.5, 1, and 1.5 h. Western blotting was used to detect the levels of RIPK1 protein. **h** HCT116 cells were transiently transfected with Flag-TRAF6 or mock control plasmid and cultured for 24 h and then incubated with MG132 (10 μM) or the same amount of DMSO for 6 h respectively. Western blotting was used to detect the protein levels of RIPK1 and p-RIPK1. **i** In HEK293T cells co-transfected with Flag-TRAF6, Myc-RIPK1, and HA-UB. Anti-Myc affinity beads were used to detect the co-immunoprecipitation of RIPK1 polyubiquitination. **j** HEK293T cells were co-transfected with Flag-RING/ZnF, Myc-RIPK1, and HA-UB, and anti-Myc affinity beads were used to detect the RIPK1 polyubiquitination. **k** HCT116 and SW480 cells were co-transfected with Flag-TRAF6, Myc-RIPK1, and HA-UB. Anti-Myc affinity beads were used to detect the RIPK1 polyubiquitination. **l** In HCT116 and SW480 cells co-transfected with Flag-TRAF6, Myc-RIPK1, and HA-UB-K48, anti-Myc affinity beads were used to detect the co-immunoprecipitation of RIPK1 polyubiquitination. **m** HEK293T cells with 0, 0.5, 1, 1.5, and 2.5 μg of Flag-TRAF6 plasmid, Myc-RIPK1, and HA-UB plasmids, respectively, and detected for RIPK1 polyubiquitination with anti-Myc affinity beads. (*p < 0.05, **p < 0.01, ***p < 0.001, with an unpaired Student’s *t* test (**f**, **g**) or one-way ANOVA analysis (**h**)).

In the above experiments, we determined that TRAF6 and RIPK1 proteins directly interact with each other through their respective key domains, so what we are more interested in is the regulation of the content and stability of RIPK1 protein by TRAF6. We performed western blotting experiments on colorectal cancer cells transiently and stably transfected with TRAF6 plasmids (Fig. 3a, b), and we found that the levels of RIPK1 and p-RIPK1 proteins in colorectal cancer cells that highly expressed TRAF6 protein were significantly reduced. Similarly, we performed western blotting experiments on colorectal cancer cells transiently transfected with Si-TRAF6 and stably transfected with Sh-TRAF6 plasmids (Fig. 3c, d). The levels of RIPK1 and p-RIPK1 proteins were significantly higher than those in the control group. Second, we constructed the KO-TRAF6 HCT116 cell line with CRISPR-Cas9 system, and the western blotting results showed that the levels of RIPK1 and p-RIPK1 protein were significantly increased in the KO-TRAF6 HCT116 cells (Fig. 3e). In conclusion, we can obtain that TRAF6 can significantly reduce the protein abundance of RIPK1.

To further explore the regulation mechanism of TRAF6 on RIPK1 protein, CHX is cycloheximide, and the mechanism of action is by binding to the 80S ribosomes and then preventing the translocation of the translation process tRNA to inhibit the protein synthesis, which has the effect of inhibiting the de novo protein synthesis in the cell. We blocked de novo protein synthesis in HCT116 and SW480 cells overexpressing TRAF6 with cycloheximide (CHX), and we could obtain that the levels of RIPK1 protein gradually decreased with the prolongation of the drug; And the cells overexpressing TRAF6 protein had a higher overall RIPK1 decrease than the mock control group, and the difference was statistically significant (Fig. 3f, g). We then inhibited the pathway of proteasome-mediated degradation in HCT116 cells by using a proteasome inhibitor (MG132). Surprisingly, it was found that MG132 could prevent the degradation of RIPK1 protein caused by TRAF6 (Fig. 3h), but the level of TRAF6 was not significantly altered (Fig. S2a, b), and the degradation mode of RIPK1 was not altered by the TNFAIP3 (A20) pathway (Fig. S2c, d). So it was boldly inferred that TRAF6 degrades RIPK1 protein-level through the proteasome pathway.

### TRAF6 induces polyubiquitination of RIPK1 through the K48 pathway

Our previous experiments demonstrated that TRAF6 degrades RIPK1 protein by mediating the proteasome pathway. TRAF6 belongs to the TRAF family, and its members have RING/ZnF domains and E3 ligase activity [5–7]. Therefore, we have experimentally shown that TRAF6 promotes the combined polyubiquitination of RIPK1 protein junctions (Fig. 3i) and that the domain E3 ligase RING/ZnF domain in TRAF6 also mediates the combined and polyubiquitination junction to RIPK1 (Fig. 3j). It was then shown that TRAF6 can be significantly linked to RIPK1 by polyubiquitination in HCT116 and SW480 colorectal cancer cells (Fig. 3k). Therefore, we can obtain that TRAF6 can directly degrade RIPK1 protein levels through polyubiquitination.

TRAF6 in HCT116 and SW480 cells compared with the mock control group could be linked to RIPK1 by K48-linked polyubiquitination (Fig. 3l). The degree of K48-linked polyubiquitination on RIPK1 was demonstrated by adding different doses of TRAF6 plasmid. The data showed that with the increase of TRAF6 plasmid content, the degree of K48-linked polyubiquitination on RIPK1 was stronger (Fig. 3m). Therefore, we can easily conclude that the E3 ligase RING/ZnF domain of TRAF6 induces the decrease of RIPK1 protein level by direct interaction to induce K48-linked polyubiquitination on RIPK1.

### TRAF6 inhibits RIPK1-RIPK3-MLKL signaling axis in colorectal cancer cells

**Fig. 4:**
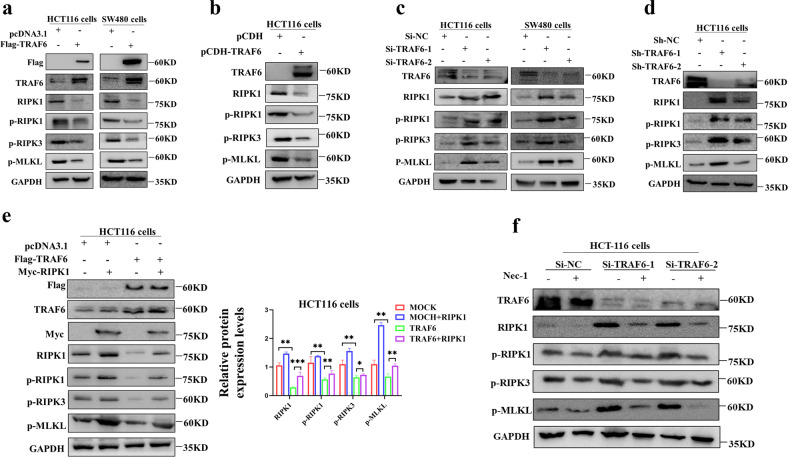
TRAF6 regulates the RIPK1-RIPK3-MLKL signaling axis. **a** An HCT116 and SW480 cells were transiently transfected with Flag-TRAF6 or mock control plasmid. Protein levels of RIPK1, p-RIPK1, p-RIPK3, and p-MLKL were detected in western blotting. **b** HCT116 cells were stably transfected with Flag-TRAF6 or mock control plasmid. Protein levels of RIPK1, p-RIPK1, p-RIPK3, and p-MLKL were detected by western blottng. **c** HCT116 and SW480 cells were transiently transfected with Si-NC, Si-TRAF6-1, or Si-TRAF6-1 plasmids. Western blotting was used to detect the protein levels of RIPK1, p-RIPK1, p-RIPK3, and p-MLKL. **d** HCT116 cells were stably transfected with Sh-NC, Sh-TRAF6-1, or Sh-TRAF6-1 plasmids. Western blotting was used to detect the protein levels of RIPK1, p-RIPK1, p-RIPK3, and p-MLKL. **e** HCT16 cells were co-transfected with Flag-TRAF6 and Myc-RIPK1 plasmids, Protein levels of RIPK1, p-RIPK1, p-RIPK3, and p-MLKL were detected in western blotting. **f** HCT116 cells were transiently transfected with Si-NC, Si-TRAF6-1, or Si-TRAF6-2 plasmids and incubated with Nec-1. Western blotting was used to detect RIPK1, p-RIPK1, p-RIPK3, and p-MLKL protein levels. (*p < 0.05, **p < 0.01, ***p < 0.001, with one-way ANOVA analysis (**e**)).

Transient (Fig. 4a) and stable (Fig. 4b) overexpression of TRAF6 in colorectal cancer cells significantly decreased the protein levels of RIPK1 and p-RIPK1 as well as downstream p-RIPK3 and p-MLKL produce inhibition. Knockdown of TRAF6 in colorectal cancer cells significantly up-regulated the protein levels of RIPK1 and p-RIPK1 and downstream p-RIPK3 and p-MLKL (Fig. 4d). Overexpressing RIPK1 showed that the negative regulation of the RIPK1-RIPK3-MLKL axis by TRAF6 could be rescued (Fig. 4e), and the difference in downstream protein levels was statistically significant. In the knockdown of the TRAF6 plasmid induced by the Nec-1 inhibitor, we interestingly found that the protein level of upstream TRAF6 was unchanged, while the downstream protein levels of p-RIPK3 and p-MLKL were significantly down-regulated (Figs. 4f and S2e). Therefore, these results suggest that TRAF6 negatively regulates the RIPK1-RIPK3-MLKL signaling axis.

### TRAF6 promotes tumor progression by inhibiting necroptosis in colorectal cancer cells

**Fig. 5:**
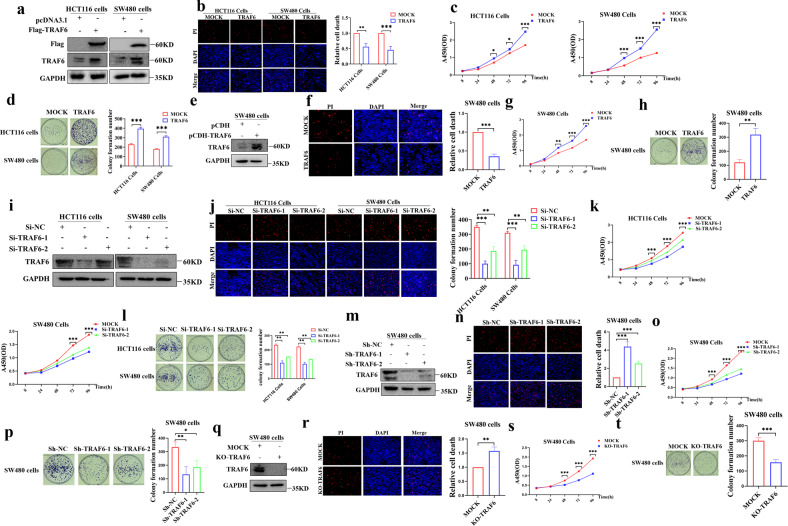
TRAF6 inhibits necroptosis to promote colorectal cancer cell proliferation. **a**–**d** HCT116 and SW480 cells were transiently transfected with Flag-TRAF6 or mock control plasmid. Western blotting was used to detect the levels of TRAF6 protein (**a**). The transfected cells were further examined and analyzed for necroptosis (**b**), as well as cell proliferation (**c**) and clonal colony formation assays (**d**). **e**–**h** SW480 cells were stably transfected with Flag-TRAF6 or mock control plasmid. Western blotting was used to detect the levels of TRAF6 protein (**e**). The transfected cells were analyzed for necroptosis (**f**), as well as cell proliferation (**g**) and clonal colony formation experiments (**h**). **i**–**l** HCT116 and SW480 cells were transiently transfected with Si-NC, Si-TRAF6-1, or Si-TRAF6-1 plasmids. Western blotting was used to detect the levels of TRAF6 protein (**i**). The transfected cells were further tested and analyzed for necroptosis (**j**), as well as cell proliferation (**k**) and clonal colony formation experiments (**l**). **m**–**p** SW480 cells were stably transfected with Sh-NC, Sh-TRAF6-1, or Sh-TRAF6-1 plasmids. Western blotting was used to detect levels of TRAF6 protein (**m**). The transfected cells were further tested and analyzed for necroptosis (**n**), as well as cell proliferation (**o**) and clonal colony formation experiments (**p**). **q**–**t** SW480 cells were transiently transfected with KO-TRAF6 or mock control plasmid. Western blotting was used to detect the levels of TRAF6 protein (**q**). The transfected cells were analyzed for necroptosis (**r**), as well as cell proliferation (**s**) and clonal colony formation experiments (**t**). (*p < 0.05, **p < 0.01, ***p < 0.001, with an unpaired Student’s *t* test (**b**–**d**, **f**–**h**, **r**–**t**) or one-way ANOVA analysis (**j**–**l**, **n**–**p**)).

We overexpressed TRAF6 protein by transient transfection and stable transfection (Fig. 5a, e), which inhibited the necroptosis of HCT116 and SW480 cells (Fig. 5b, f), and promoted cell proliferation and clonal colony formation (Fig. 5c, d, g, h). Conversely, TRAF6 protein was successfully knocked down by transient and stable transfection (Fig. 5i, m). Necroptosis data in HCT116 and SW480 cells showed that low expression of TRAF6 significantly up-regulated necroptosis in cells (Fig. 5j, n), and the difference was statistically significant, which inhibited cell proliferation and clonal colony formation (Fig. 5k, l, o, p). In SW480 cells constructed with KO-TRAF6 by the CRISPR-Cas9 system (Fig. 5q), it was found that the necroptosis of KO-TRAF6 in colorectal cancer cells was significantly higher than that of the mock control group (Fig. 5r) while inhibiting cell proliferation and clonal colony formation (Fig. 5s, t). These experimental results suggest that TRAF6 inhibits necroptosis in colorectal cancer cells by inhibiting the RIPK1-RIPK3-MLKL signaling axis, resulting in cancer cell progression.

### To confirm the inhibition of TRAF6 on necroptosis of colorectal cancer cells

**Fig. 6:**
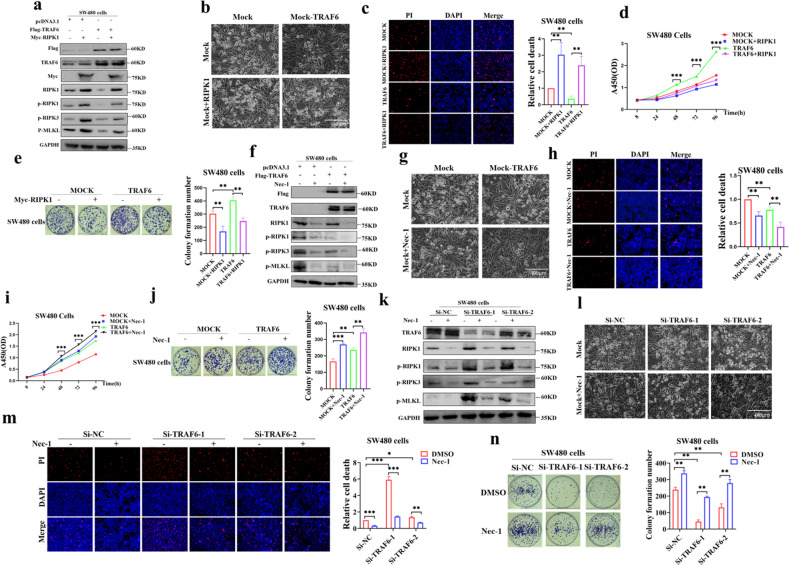
TRAF6 promotes cell proliferation by inhibiting colorectal necroptosis through the RIPK1-RIPK3-MLKL axis. **a**–**e** SW480 cells were co-transfected with Flag-TRAF6 and Myc-RIPK1 plasmids. Protein levels of TRAF6, RIPK1, RIPK3, and MLKL were detected in western blotting (**a**). The images were obtained by light microscopy (**b**). Necroptosis of the transfected cells was detected and analyzed (**c**), as well as cell proliferation (**d**) and clonal colony formation (**e**). **f**–**j** SW480 cells were transfected with Flag-TRAF6 or mock plasmid and incubated with Nec-1. The protein levels of TRAF6, RIPK1, RIPK3, and MLKL were detected in western blotting (**f**). The images were obtained in light microscopy (**g**). Necroptosis of the transfected cells was analyzed (**h**), as well as cell proliferation (**i**) and clonal colony formation (**j**). **k**–**n** SW480 cells were transiently transfected with Si-NC, Si-TRAF6-1, or Si-TRAF6-1 plasmids and incubated with Nec-1. The protein levels of TRAF6, RIPK1, RIPK3, and MLKL were detected in western blotting (**k**). Images were obtained in light microscopy (**l**). Necroptosis of the transfected cells was analyzed (**m**) and clonal colony formation (**n**). (*p < 0.05, **p < 0.01, ***p < 0.001, with one-way ANOVA analysis (**c**–**e**, **h**–**j**, **m**, **n**)).

To confirm whether TRAF6 could induce tumor progression through the inhibition of necroptosis in colorectal cancer cells through the RIPK1-RIPK3-MLKL axis, we co-transfected TRAF6 and RIPK1 into SW480 cells. Interestingly, we found that overexpression of RIPK1 could rescue protein-level inhibition of the RIPK1-RIPK3-MLKL signaling axis by TRAF6 (Fig. 6a), upregulating necroptosis (Figs. 6b, c and S2f), and inhibit cell proliferation (Fig. 6d) and clonal colony formation (Fig. 6e); Nec-1 could further downregulate TRAF6-mediated repression of RIPK1-RIPK3-MLKL signaling axis in protein levels (Fig. 6f), further reducing cell necroptosis (Figs. 6g, h and S2g), which accelerated cell proliferation (Fig. 6i) and clonal colony formation (Fig. 6j); Nec-1 treatment in transient transfection of knockdown TRAF6 plasmid, down-regulated RIPK1-RIPK3-MLKL signaling axis protein levels inhibition (Fig. 6k) and reduced necroptosis (Figs. 6l, m and S2h), and accelerated clonal colony formation (Fig. 6n). We also performed Annexin V^+^-PI^+^ flow cytometry experiments to verify the effects of the protective agent Nec-1s and the necroptosis inducer TSZ on the cells. The results showed that knockdown of TRAF6 in HCT116 and SW480 cells could cause necroptosis (Annexin V^+^-PI^+^) which could be recovered by Nec-1s inhibitor. And necroptosis induced by the necroptosis inducer TSZ in HCT116 and SW480 cells could be inhibited by TRAF6 (Figs. S3a, b and S4a, b). Therefore, we could confirm that TRAF6 in colorectal cancer cells induces tumor progression by inhibiting the RIPK1-RIPK3-MLKL necroptosis signaling axis.

### Ubiquitination domains

**Fig. 7:**
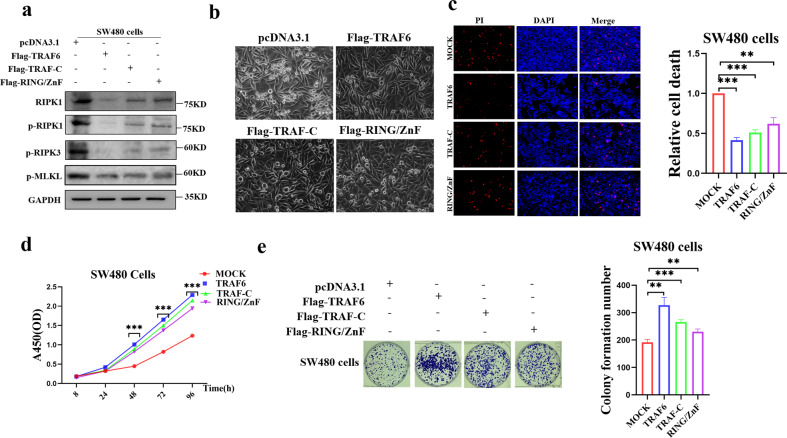
TRAF6 promotes colorectal cancer cell proliferation by inhibiting necroptosis through TRAF-C and RING/ZnF domains. **a**–**e** SW480 cells were co-transfected with Flag-TRAF-C or Flag-RING/ZnF plasmids, and the protein levels of TRAF6, RIPK1, RIPK3, and MLKL were obtained in western blotting (**a**), and images were obtained in light microscopy (**b**). Necroptosis of the transfected cells was analyzed (**c**), as well as cell proliferation (**d**) and clonal colony formation (**e**). (*p < 0.05, **p < 0.01, ***p < 0.001, with one-way ANOVA analysis (**c**–**e**)).

Our previous study found that the TRAF-C domain is the key domain that mediates the direct mutual ubiquitination between TRAF6 and RIPK1, while RING/ZnF is required as an E3 ubiquitin ligase for TRAF6 and is an important functional region of the TRAF6 protein; By transiently transfecting TRAF6, TRAF-C and RING/ZnF plasmids in SW480 cells, respectively, it was found that TRAF-C and RING/ZnF plasmids produced significant inhibition of RIPK1-RIPK3-MLKL axis protein levels compared with mock control (Fig. 7a); The death rate in the TRAF-C and RING/ZnF plasmid transfected cells decreased compared with the mock control group (Figs. 7b and S2i), and necroptosis was significantly inhibited (Fig. 7c), and the cell proliferation was up-regulated (Fig. 7d).) and clonal colony formation (Fig. 7e). In conclusion, we can infer that the inhibitory effect of TRAF6 on the RIPK1-RIPK3-MLKL necroptosis signaling axis is mediated through the TRAF-C and RING/ZnF functional domains.

### TRAF6 in colorectal cancer cells promotes tumorigenesis in a xenograft model by inhibiting the RIPK1-RIPK-MLKL signaling axis

**Fig. 8:**
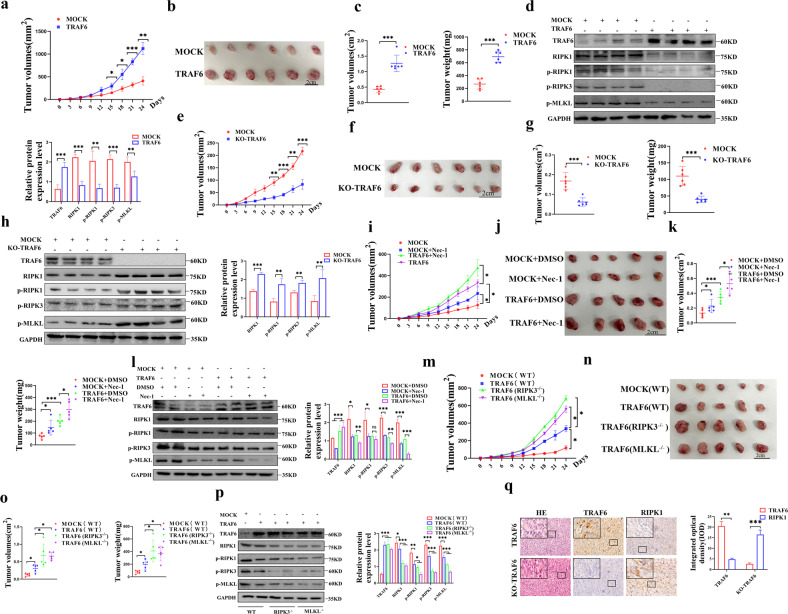
Xenograft tumor model to demonstrate that TRAF6 inhibits RIPK1-RIPK3-MLKL signaling axis in colorectal cancer cells. **a**–**d** A subcutaneous tumor model of BALB/c female nude mice with high protein levels of TRAF6 cells was established. Tumors were measured every 3 days (**a**). The images show the tumors formed by the mice from the overexpression of TRAF6 in HCT116 cells compared to control tumors group (**b**). The volume and weight of the tumor tissue were statistically analyzed (**c**). Protein expression levels of the RIPK1-RIPK3-MLKL signaling axis were detected in western blotting (**d**). **e**–**h** BALB/c female nude mice were subcutaneously transplanted with CRISPR/Cas9 gene knockout TRAF6 cells. Tumors were measured every 3 days (**e**). The images show the tumors formed by the mice from the KO-TRAF6 in HCT116 cells and control cells (**f**). Tumor volume and weight (**g**) were statistically analyzed. Protein expression levels of the RIPK1-RIPK3-MLKL signaling axis were detected in western blotting (**h**). **i**–**l** A subcutaneous tumor model of BALB/c female nude mice with high expression of TRAF6 in HCT116 cells treated with Nec-1, tumors were measured every 3 days (**i**). The images show the tumors formed by mice in each group of MOCK + DMSO, MOCK + Nec-1, TRAF6 + DMSO, and TRAF6 + Nec-1 (**j**). The volume and weight of tumor tissue were statistically analyzed (**k**). Protein expression levels of the RIPK1-RIPK3-MLKL signaling axis were detected in western blotting (**l**). **m**–**p** To construct a subcutaneous high expression TRAF6 cell transplantation tumor model with RIPK3^−/−^ or MLKL^−/−^ C57BL/6 genetic background, and tumor was measured every 3 days (**m**). The images show the tumors (**n**) isolated from mice in each group of MOCK (WT), TRAF6 (WT), TRAF6 (RIPK3^−/−^), and TRAF6 (MLKL^−/−^). The volume and weight (**o**) of tumor tissue were analyzed statistically. Protein expression levels of the RIPK1-RIPK3-MLKL signaling axis were detected in western blotting (**p**). **q** HE staining and immunohistochemical staining was performed on serial paraffin sections of xenografts of TRAF6-overexpressing TRAF6 cell xenografts and CRISPR/Cas9 knockout TRAF6 xenografts, showing the tissue structure of xenografts, as well as the protein expression of TRAF6 and RIPK1 level. Pictures were captured with an inverted microscope (×400). integrated optical density (IOD) detection was performed on the immunohistochemical results, and the bars represent integrated optical density (IOD) ± standard deviation (SD). (*p < 0.05, **p < 0.01, ***p < 0.001, with an unpaired Student’s *t* test (**a**–**h**, **q**) or one-way ANOVA analysis (**i**–**p**)).

The results showed that compared with the mock control group, the overexpression of TRAF6 in HCT116 cells significantly promoted the growth of xenograft tumors (Fig. 8a, b). In addition, statistical analysis of the resected xenografts showed that the TRAF6-overexpressing group significantly increased the volume and weight of the xenografts (Fig. 8c). The protein levels of RIPK1, p-RIPK1, p-RIPK3, and p-MLKL were significantly reduced, which further indicated that TRAF6 inhibits the RIPK1-RIPK3-MLKL signaling axis (Fig. 8d). On the contrary, the results showed that compared with the mock control group, the KO-TRAF6 group significantly inhibited the growth of xenograft tumors (Fig. 8e, f). Statistical analysis of the resected xenografts showed that the KO-TRAF6 in the HCT116 cells group significantly suppressed the volume and weight of the xenografts (Fig. 8g). The protein levels of RIPK1, p-RIPK1, p-RIPK3, and p-MLKL were significantly increased (Fig. 8h).

Nec-1 treatment significantly promoted the growth of xenograft tumors (Fig. 8i, j) with increased volume and weight (Fig. 8k). The protein levels of RIPK1, p-RIPK1, p-RIPK3, and p-MLKL were decreased in the Nec-1 treatment group (Fig. 8l), so we obtained that the inhibitory effect of TRAF6 on RIPK1 could be further enhanced by Nec-1.

To further verify the regulatory relationship between TRAF6 and the RIPK1-RIPK3-MLKL signaling axis, we performed a subcutaneous transplantation tumor model of high TRAF6 expressing cells through RIPK3^−/−^ and MLKL^−/−^ mice of C57BL/6 genetic background (Fig. S5a). Compared with the WT group, we found that the TRAF6(RIPK3^−/−^) and TRAF6(MLKL^−/−^) significantly promoted the growth of xenograft tumors (Fig. 8m, n) with increased the volume and weight (Fig. 8o). There was a significant decrease in p-RIPK3 and p-MLKL in the TRAF6(RIPK3^−/−^) and TRAF6(MLKL^−/−^) compared with the TRAF6 (WT) (Fig. 8p). The results indicated that the inhibitory effect of TRAF6 on the RIPK1-RIPK3-MLKL signaling axis could be further inhibited by the lack of RIPK3 or MLKL protein levels. Finally, we confirmed the negative correlation between differentially-expressed TRAF6 and RIPK1 through immunohistochemical experiments on xenograft tumors, and the results were statistically significant (Fig. 8q). We then performed subcutaneous MOCK(TRAF6), MOCK(WT), MOCK(RIPK3^−/−^) and MOCK (MLKL^−/−^) group of cell transplantation tumor models through RIPK3^−/−^ and MLKL^−/−^ mice of C57BL/6 genetic background. We obtained results indicating that the TRAF6-induced inhibition could be compensated by reducing RIPK3 or MLKL protein levels (Fig. S5b–g). In conclusion, we systematically confirmed the inhibitory effect of TRAF6 on the RIPK1-RIPK3-MLKL signaling axis in vivo and promoted the growth of colorectal cancer cells in a xenograft tumor model.

**Fig. 9:**
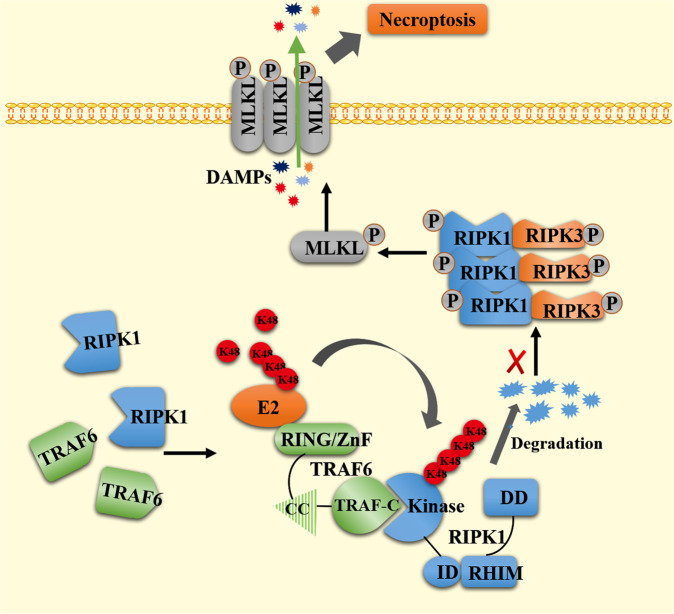
Schematic diagram of TRAF6-regulated necroptosis signaling axis. Schematic diagram demonstrates the TRAF-C domain of TRAF6 interacts directly with the KD domain of RIPK1. The K48-linked polyubiquitination of RIPK1 caused a decrease in the protein levels of RIPK1, which affects changes in the RIPK-RIPK3-MLKL necroptosis signaling axis, ultimately leading to the development of colorectal cancer cells.

In conclusion, we demonstrate for the first time that TRAF6 reduces necroptosis in colorectal cancer cells by directly degrading RIPK1 protein levels and inhibiting the RIPK1-RIPK3-MLKL signaling axis (Fig. 9), resulting in the promotion of tumor progression. These results suggest that promoting necroptosis of cancer cells by downregulating TRAF6 may be used in a new direction for the treatment of colorectal cancer.

## Discussion

Colorectal cancer is a common malignant tumor of the digestive tract, and the therapeutic effect is still a challenge internationally. Although chemotherapy is currently the main method for refractory colorectal cancer, with the addition of targeted therapy and immunotherapy, the therapeutic effect has been improved by leaps and bounds [26]. Therefore, finding a key and effective target site is still a hot topic in the treatment of colorectal cancer. In this study, we found that TRAF6 enhances the progression of colorectal cancer cells by inhibiting RIPK1-mediated necroptosis. Our experiments have confirmed that TRAF6 protein interacts directly with the KD domain of RIPK1 protein through its own TRAF-C domain in vitro and in vivo. The C-terminal TRAF-C domain of TRAF6 is an important signal-binding protein that promotes the oligomerization of proteins, has recognition sites for ligands and receptors [27], and can interact with a variety of kinases. The KD domain of RIPK1 is an N-terminal kinase domain that plays an important role in the signaling cascade of inflammatory responses and necroptosis [28]. Further studies found that RING/ZnF has an E3 ubiquitin ligase activity domain to induce K48-linked polyubiquitination of RIPK1. The RING/ZnF domain is an important activation domain of TRAF6 and also acts as a ubiquitin ligase E3, which can bind to E2 ubiquitin-conjugating enzymes to mediate polyubiquitination ligation [29–31]. Ultimately, it leads to the degradation of RIPK1 protein, down-regulates the downstream necroptosis pathway proteins, and promotes cancer cell progression by inhibiting necroptosis. This is consistent with the conclusion that Nugues et al. in acute leukemia [32] and Koo et al. in breast cancer [8] found that necroptosis can inhibit the proliferation of tumor cells. Therefore, we identified TRAF6 as a novel target molecule against necroptosis in colorectal cancer cells.

Our study confirmed that TRAF6 promotes tumor progression by inhibiting necroptosis mediated by the RIPK1-RIPK3-MLKL signaling pathway. Necroptosis is the formation of Complex IIb composed of RIPK1, RIPK3, FADD, and pro-caspase-8. Degradation of RIPK1 affects the formation of Complex IIb [17, 33–36]. A decrease in the protein levels of p-RIPK1, p-RIPK3, and p-MLKL mediated by TRAF6 protein will inhibit the formation of necrosome and the oligomerization of phosphorylated MLKL in necroptosis. Nec-1, a small molecule inhibitor, is a variant inhibitor of RIPK1 that inhibits RIPK1 activity by acting on the T-loop structure of RIPK1 [37–39], which then inhibits the formation of the RIPK1-RIPK3 complex and blocks downstream necrosis, and it stabilizes the specific inactive conformation of the kinase structural domain [40]. At the same time, it was found that the inhibition of TRAF6 expression can be overcome by Nec-1. Although it has been reported that Nec-1 has some off-target effects in vitro and in vivo, which weakens the experimental efficiency to some extent [13, 41], Nec-1 is still a good inhibitor of necroptosis in vivo, which can significantly inhibit the necroptosis in vitro and in vivo, and is of great significance to the experiment [14, 42–45].

In conclusion, we demonstrated that TRAF6 promotes cancer cell progression by inhibiting necroptosis via RIPK1 in colorectal cancer cells. TRAF6 acts on RIPK1 through K48-linked polyubiquitination, resulting in the inhibition of the RIPK1-RIPK3-MLKL necroptosis signaling pathway. This study is a novel signaling potential target for colorectal cancer treatment and may provide a new therapeutic target from a new perspective of colorectal cancer cell death, which has important clinical significance.

## Supplementary information

Supplement material

aj-checklist

Uncropped blot images

The patients agreement

## Data Availability

The data used and/or analyzed in this study can be obtained from the corresponding author on reasonable request.
