# Supplementary material for: TRAF6 regulates the abundance of RIPK1 and inhibits the RIPK1/RIPK3/MLKL necroptosis signaling pathway and affects the progression of colorectal cancer
Source: Cell Death Dis. 2023 Jan 5;14(1):6. doi: 10.1038/s41419-022-05524-y (PMC9816173; doi:10.1038/s41419-022-05524-y)
Supplement: Supplementary file 1 — Supplement material [file 41419_2022_5524_MOESM1_ESM.docx]

**TRAF6 regulates the abundance of RIPK1 and inhibits the RIPK1/RIPK3/MLKL necroptosis signaling pathway and affects the progression of** **colorectal cancer**

Penghang Lin^1*^, Chunlin Lin^1*^, Ruofan He^1,2^, Hui Chen^1,2^, Zuhong Teng^1,2^, Hengxin Yao^1,2^, Songyi Liu^1,2^, RobertM.Hoffman^3,4^, Jianxin Ye^1#^, Guangwei Zhu1^1#^,

1. Department of Gastrointestinal Surgery 2 Section, Institute of Abdominal Surgery, Key Laboratory of accurate diagnosis and treatment of cancer, The First Hospital Affiliated to Fujian Medical University, Fuzhou 350005, China;2. Key Laboratory of Ministry of Education for Gastrointestinal Cancer, Fujian Medical University, Fuzhou 350000, China;3. AntiCancer, Inc., San Diego, CA, U.S.A.;4. Department of Surgery, University of California, San Diego, CA, U.S.A.

#Corresponding author: Jian-Xin Ye, Department of Gastrointestinal Surgery 2

Section, Institute of Abdominal Surgery, Key Laboratory of Accurate Diagnosis and Treatment of Cancer, The First Hospital Affiliated to Fujian Medical University, 20th, Chazhong Road, Fuzhou, Fujian 350005, China. Tel: +86-138-0955-3280; E-mail: [yejianxinfuyi@126.com](mailto:yejianxinfuyi@126.com)

Guangwei Zhu, Department of Gastrointestinal Surgery 2 Section, Institute of Abdominal Surgery, Key Laboratory of Accurate Diagnosis and Treatment of Cancer, The First Hospital Affiliated to Fujian Medical University, 20th, Chazhong Road, Fuzhou, Fujian 350005, China. Tel: +86-180-6047-3703; E-mail: zgwzsy@126.com

*These authors contributed equally to this study.

**SUPPLEMENTAL METHODS**

**Flow cytometry assay**

Necrotic apoptotic cells detection Necrotic cells were quantified using the FITC Annexin V apoptosis assay kit (Gibco). Collected cells were washed with PBS buffer, resuspended in binding buffer, and incubated with Annexin V-FITC and PI for room temperature for 15 min in the dark. Stained cells were detected by flow cytometry within 1h. Cells were incubated with the TSZ (Beyotime) necrosis inducer kit or the Nec-1s (MedChemExpress) necrosis inhibitor kit for 12 h at 37°C.


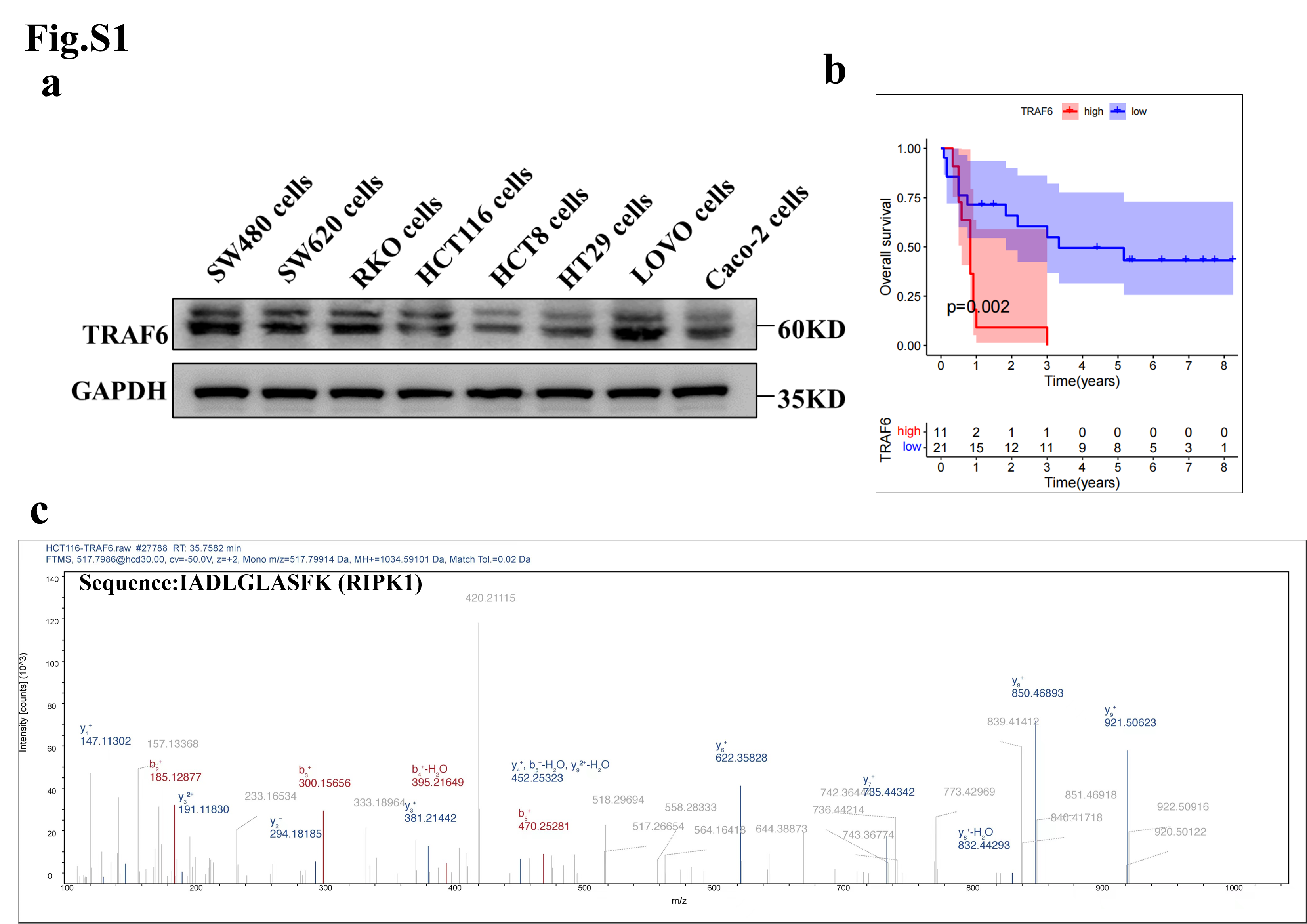


**Supplementary Fig.1** a Expression levels of TRAF6 protein in different colorectal cancer cell lines. b Dataset GSE16125 of colorectal cancer patients through the GEO public data platform to perform survival analysis of high and low expression levels of TRAF6 protein. c The purified TRAF6 protein was extracted for immunoprecipitation with HCT116 cells by in vitro transcription and translation systems, and the precipitated products were analyzed by LC-M S/MS results.


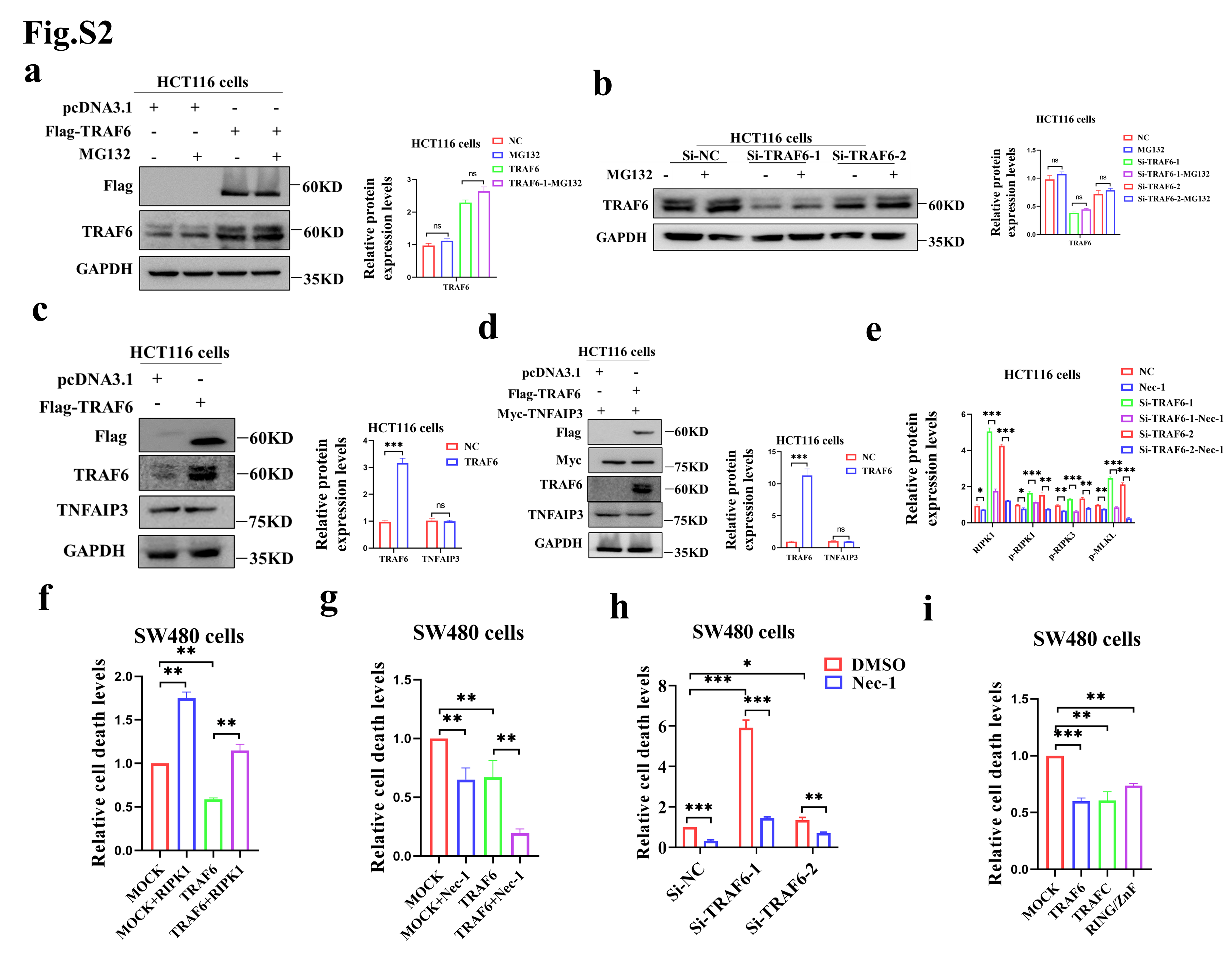


**Supplementary Fig.2** a The cells were transiently transfected into HCT116 cells with a Flag-TRAF6 or a mock control plasmid for 24h, then coincubated with MG132 (10uM) or equal amounts of DMSO for 6h, and TRAF6 protein expression was measured by Western blot. b HCT116 cells were transiently transfected with Si-NC, Si-TRAF6-1 or Si-TRAF6-2 plasmids for 24h, then coincubated with MG132 (10uM) or equal amounts of DMSO for 6h, and TRAF6 protein expression was measured by Western blot. c HCT116 cells were transiently transfected with Flag-TRAF6 or a mock control plasmid for 48h, and TRAF6 and TNFAIP3 protein expression was measured by Western blot. d HCT116 cells were transiently cotransfected with Flag-TRAF6 and Myc-TNFAIP3 plasmids for 48h, and TRAF6 and TNFAIP3 protein expression was determined by Western blot. e HCT116 cells were transiently transfected with Si-NC, Si-TRAF6-1 or Si-TRAF6-2 plasmids and co-incubated with Nec-1. The levels of RIPK1, p-RIPK1, RIPK3, p-RIPK3, MLKL and p-MLKL proteins were detected by Western blot and statistically analyzed. f SW480 cells were co-transfected with Flag-TRAF6 and Myc-RIPK1 plasmid for light microscopic (X200) cell status changes, statistical analysis of the dead cells, and the results were presented in a bar chart. g SW480 cells were transfected with Flag-TRAF6 or mock plasmid and incubated with Nec-1 to observe light microscopic (X200) cell status changes. Statistical analysis of the dead cells was presented in a bar chart. h SW480 cells were transiently transfected with Si-NC, Si-TRAF6-1 or Si-TRAF6-2 plasmids and incubated with Nec-1 to observe changes in light microscopy (X200) cell status, statistical analysis of dead cells, and the results are presented in a bar graph. i Flag-TRAF6, Flag-TRAFC and Flag-RING/ZnF plasmids were used in transfected SW480 cells. The changes in cell status were observed under light microscopy (X200), and the statistical analysis of the dead cells was performed, and the results were presented as bar graphs. (*P<0.05, **P<0.01, ***P<0.001, with an unpaired Student’s t-test(a-d, f, h) or one-way ANOVA analysis(e, g, i).)


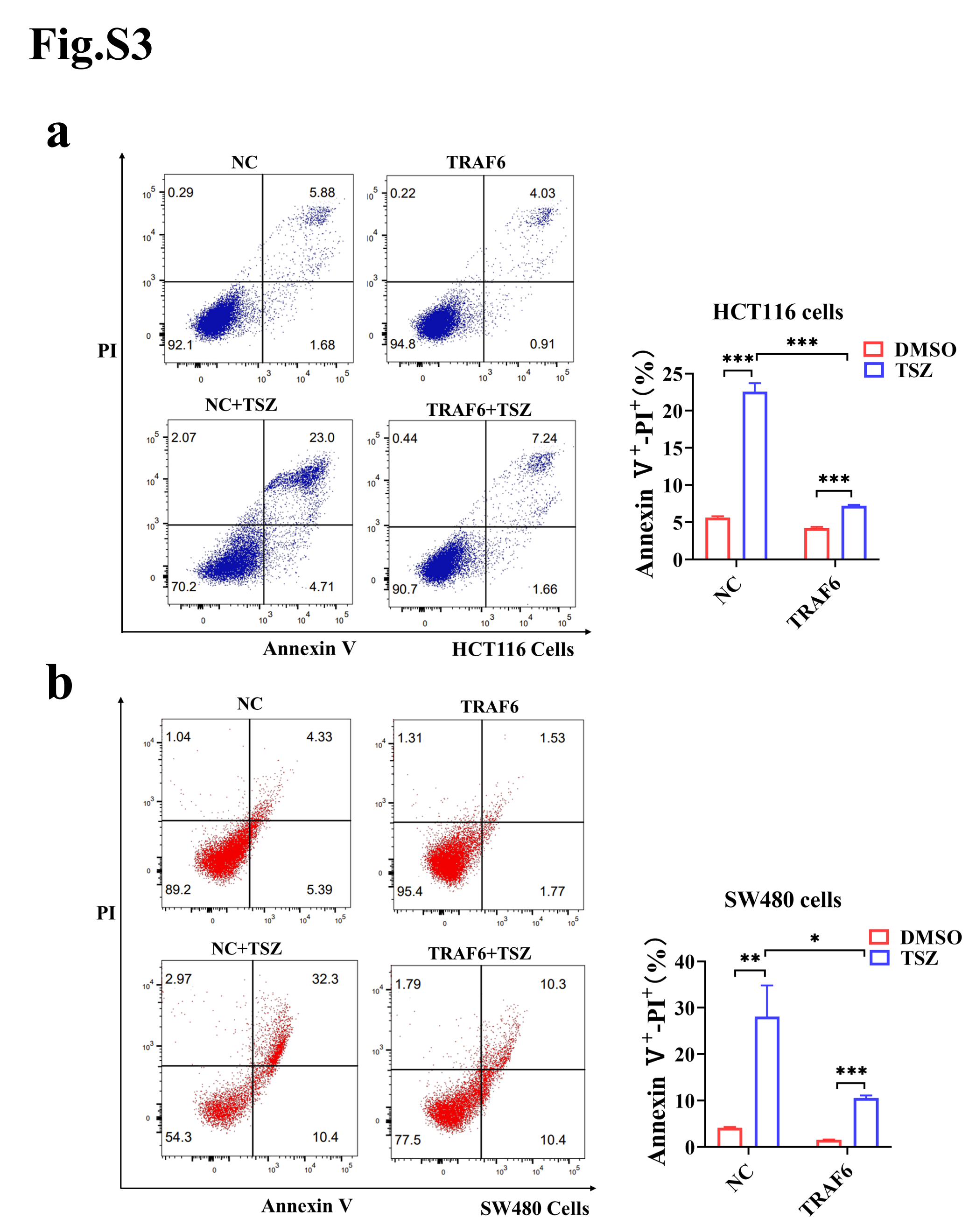


**Supplementary Fig.3** a, b The TRAF6 plasmid was transiently transfected in HCT116 and SW480 cells and was incubated 24h after being co-incubated with TSZ using the FITC Annexin V apoptosis assay kit and incubated with Annexin V-FITC and PI at room temperature for 15 min in the dark. Stained cells were detected by flow cytometry within 1h and statistical analyzed of Annexin V-FITC + and PI + results, which were presented in a bar graph. (*P<0.05, **P<0.01, ***P<0.001, with an unpaired Student’s t-test(a, b))


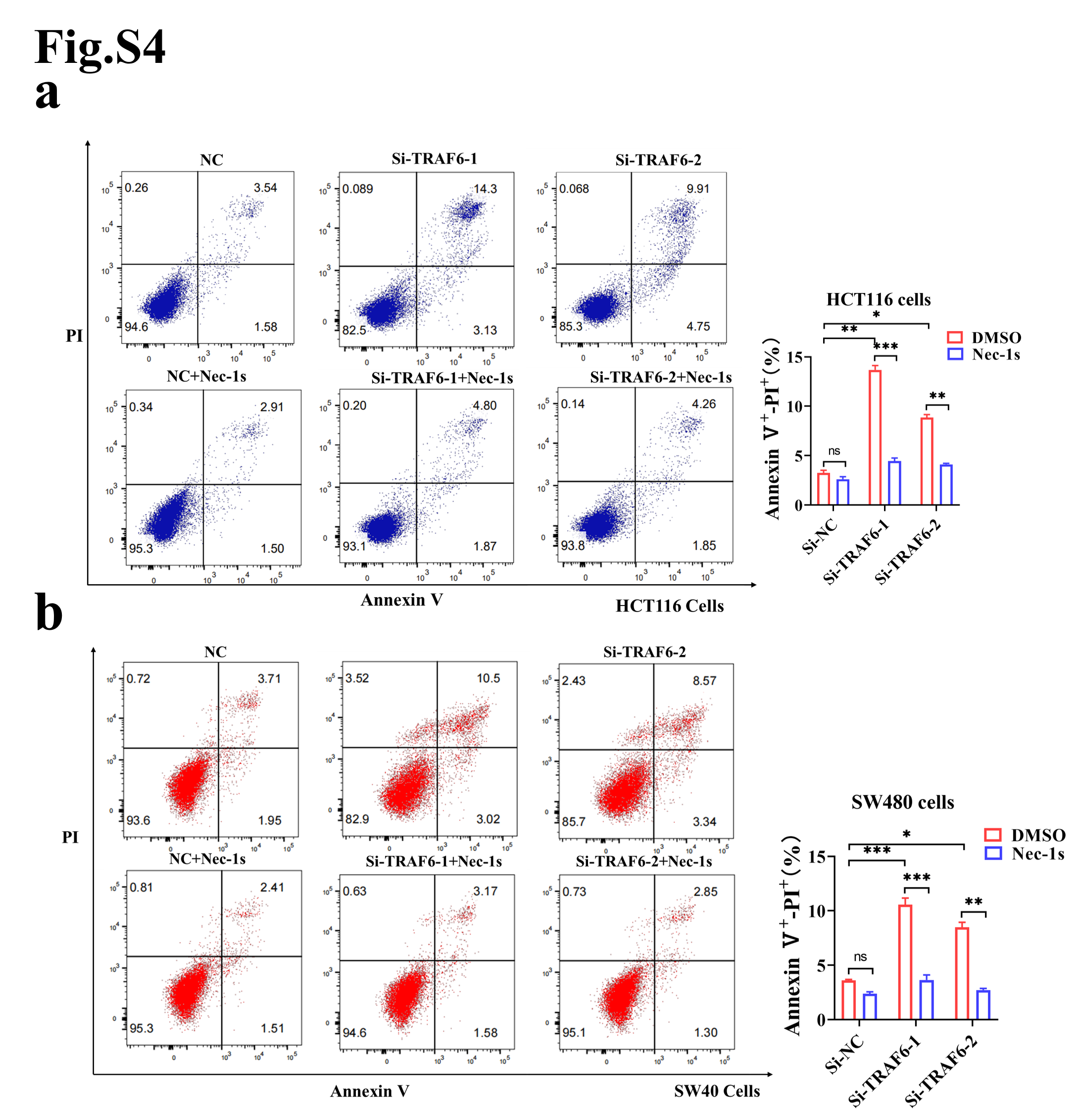


**Supplementary Fig.4** a, b HCT116 and SW480 cells were transiently transfected with Si-NC, Si-TRAF6-1 or Si-TRAF6-2 plasmids and co-incubated with Nec-1s. The FITC Annexin V apoptosis detection kits were used and incubated with Annexin V-FITC and PI at room temperature in the dark for 15 min, and the stained cells were detected by flow cytometry within 1h. The results of Annexin V-FITC + and PI + were also statistically analyzed, and the results are presented in bar graphs. (*P<0.05, **P<0.01, ***P<0.001, with an unpaired Student’s t-test(a, b))


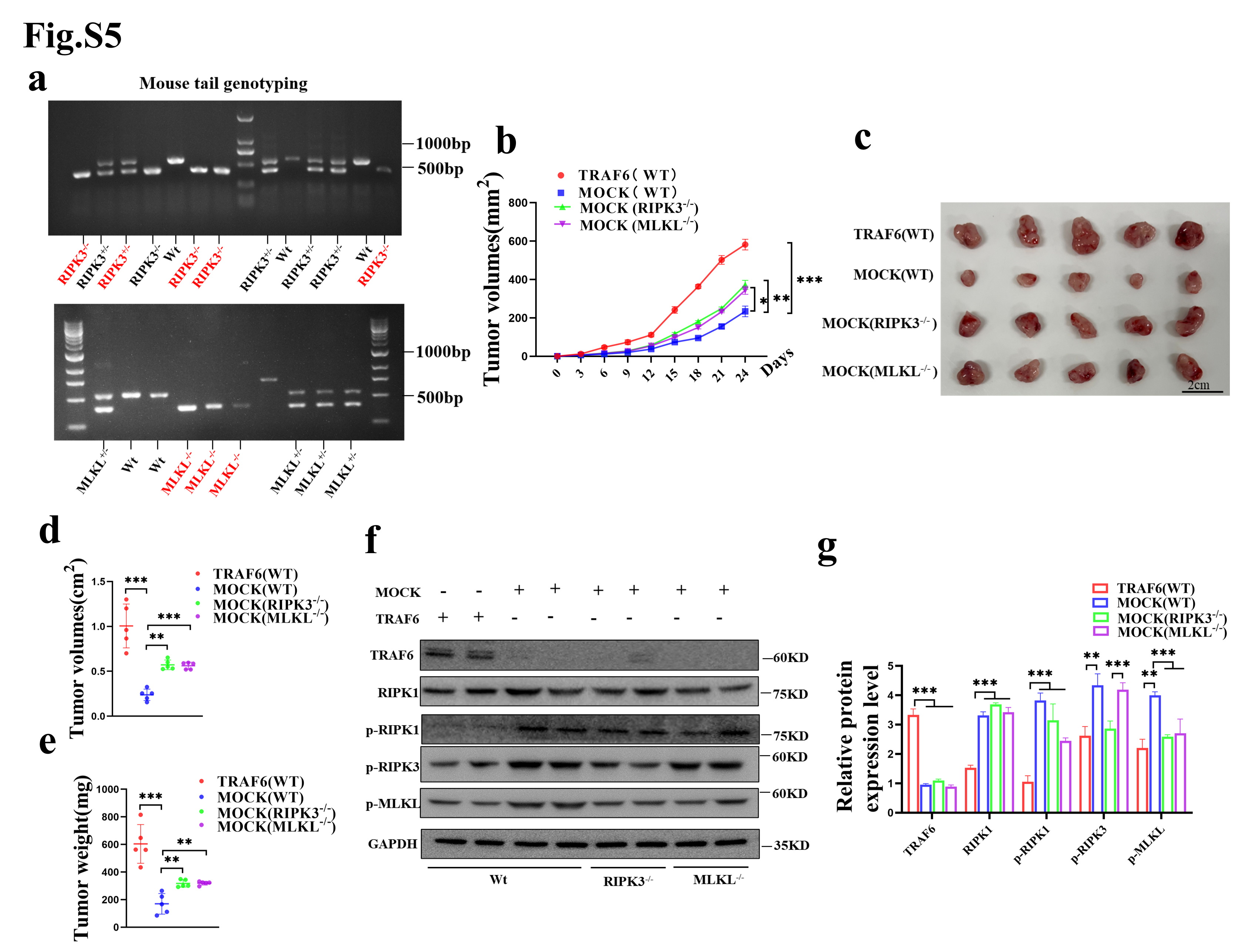


**Supplementary Fig.5** a Screening of RIPK3^−/−^ and MLKL^−/−^ knockout C57BL/6 mice with PCR. b-g To establish a subcutaneous xenograft model of RIPK3^−/−^ and MLKL^−/−^ cells with high expression of TRAF6 in MC38 cells, and tumor (b) was measured every 3 days. The images show the tumors (c) isolated from mice in each group of MOCK (WT), TRAF6 (WT), TRAF6 (RIPK3^−/−^), and TRAF6 (MLKL^−/−^). The volume and weight (d, e) of tumor tissue were analyzed statistically. Protein expression levels of the RIPK1-RIPK-MLKL signaling axis was detected in western blotting (f, g). (*P<0.05, **P<0.01, ***P<0.001 , with one-way ANOVA analysis(b-g))

| Table S1. Clinical data of 10 patients with colorectal cancer | | | | |
| --- | --- | --- | --- | --- |
| Number | Gender | Age(years) | Histological type | Degree of differentiation |
| T1 | Male | 75 | Adenocarcinoma | Moderate |
| T2 | Male | 63 | Adenocarcinoma | Moderate |
| T3 | Female | 74 | Adenocarcinoma | High |
| T4 | Male | 59 | Adenocarcinoma | Moderate |
| T5 | Female | 87 | Adenocarcinoma | High |
| T6 | Male | 55 | Adenocarcinoma | Moderate |
| T7 | Male | 66 | Adenocarcinoma | Moderate |
| T8 | Male | 67 | Adenocarcinoma | Low |
| T9 | Female | 63 | Adenocarcinoma | Moderate |
| T10 | Male | 61 | Adenocarcinoma | Moderate |
| Number | T stage | N stage | M stage | Pathologic diagnosis |
| T1 | T4 | N0 | M0 | Rectal malignant tumor |
| T2 | T3 | N0 | M1 | Rectal malignant tumor |
| T3 | T1 | N0 | M0 | Rectal malignant tumor |
| T4 | T3 | N1 | M1 | Rectal malignant tumor |
| T5 | T4 | N2 | M0 | Rectal malignant tumor |
| T6 | T4 | N2 | M0 | Malignant tumor of ascending colon |
| T7 | T4 | N1 | M0 | Malignant tumor of sigmoid colon |
| T8 | T3 | N1 | M0 | Rectal malignant tumor |
| T9 | T3 | N0 | M0 | Malignant tumor of sigmoid colon |
| T10 | T4 | N2 | M0 | Malignant tumor of ascending colon |
