# Supplementary material for: TRAF6 regulates the abundance of RIPK1 and inhibits the RIPK1/RIPK3/MLKL necroptosis signaling pathway and affects the progression of colorectal cancer
Source: Cell Death Dis. 2023 Jan 5;14(1):6. doi: 10.1038/s41419-022-05524-y (PMC9816173; doi:10.1038/s41419-022-05524-y)

Fig. 1b

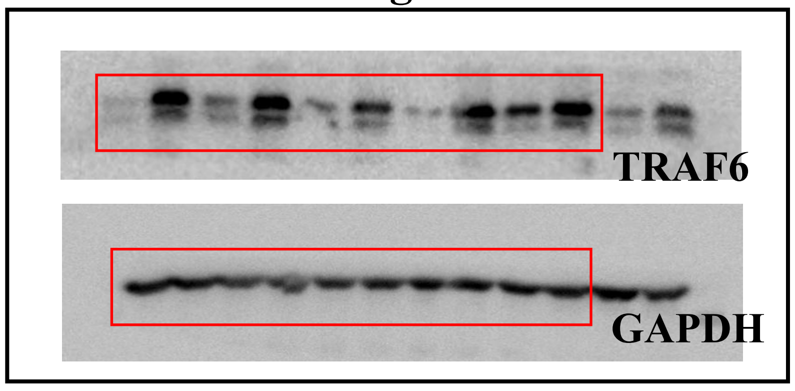

Fig. 1e

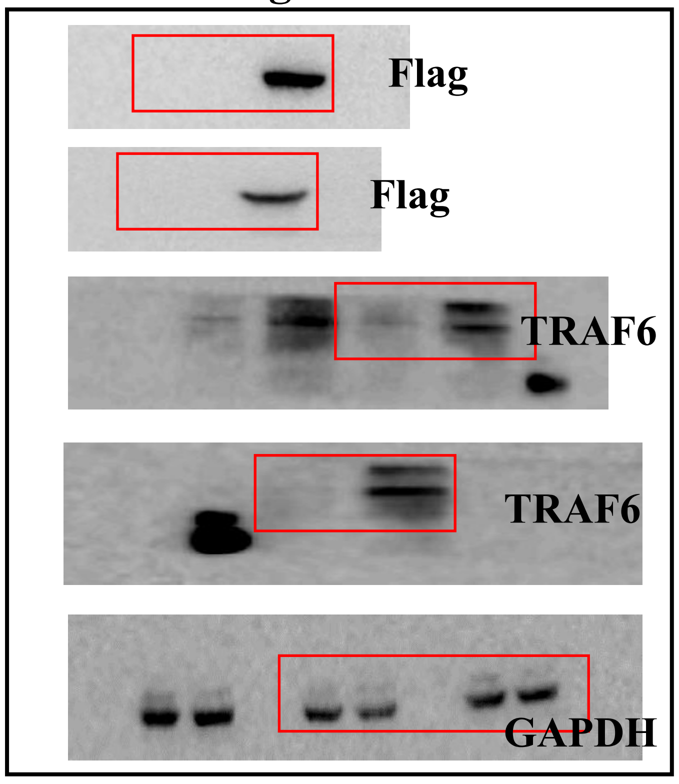

Fig. 1f

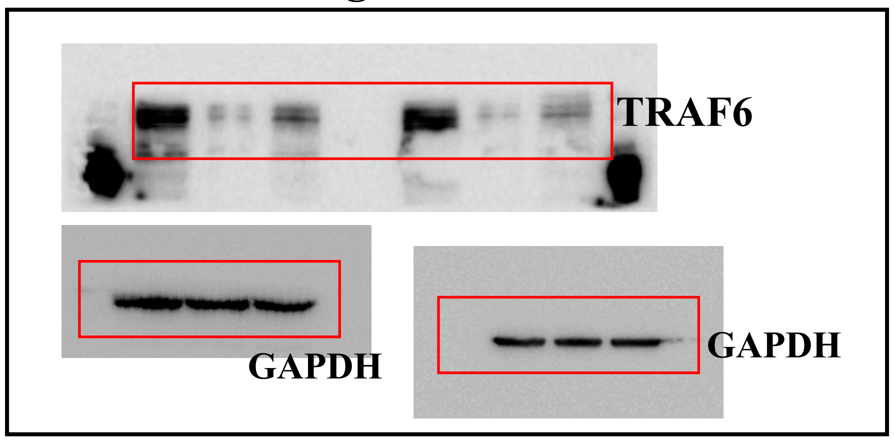

Fig. 2b

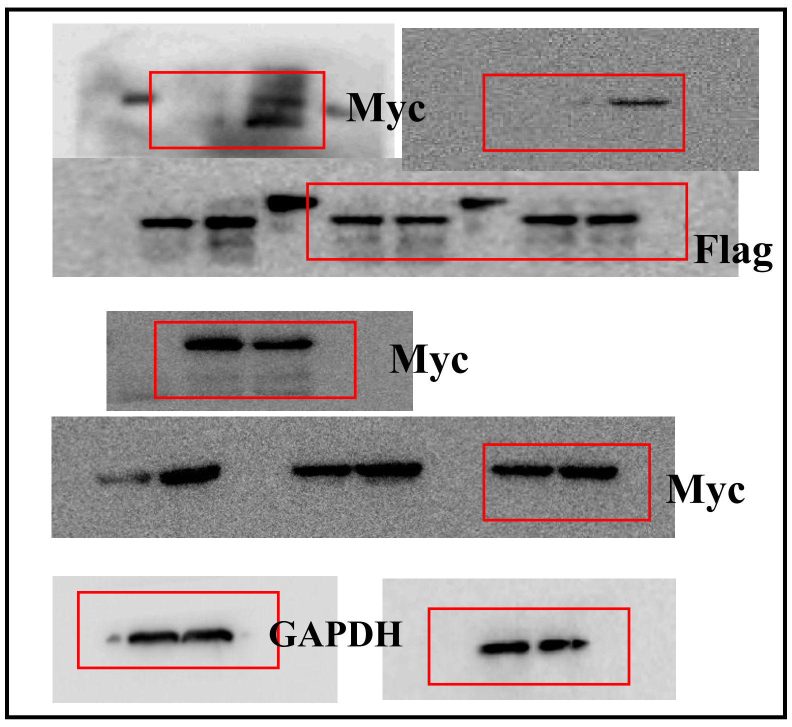

Fig. 2c

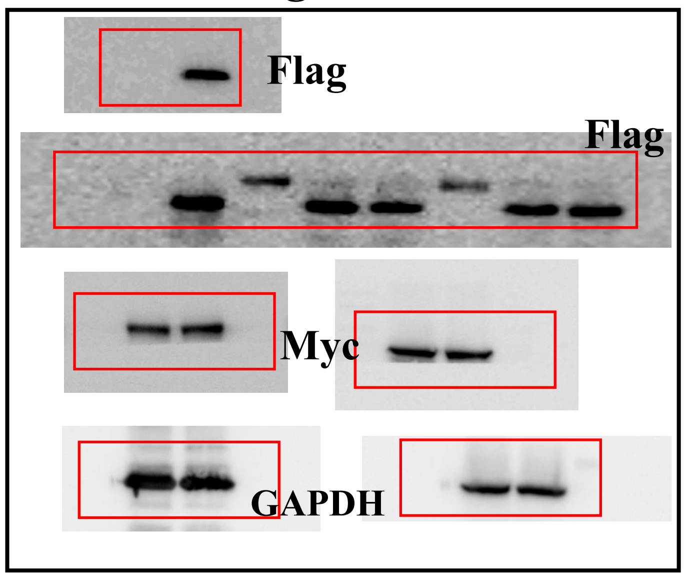

Fig. 2d/e

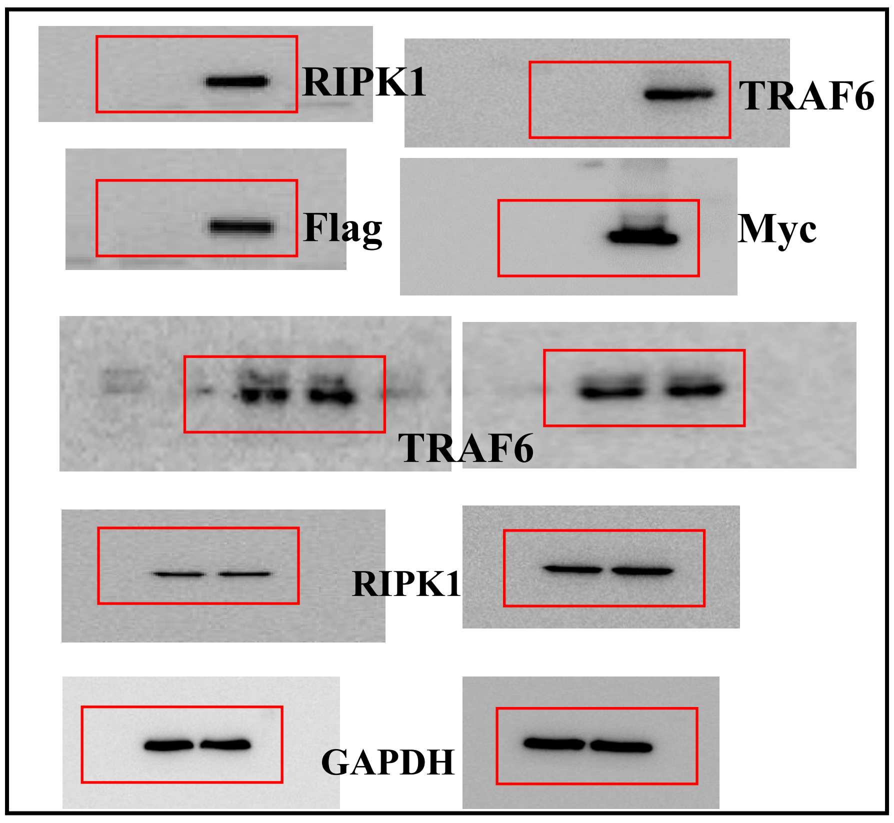

Fig. 2f/g

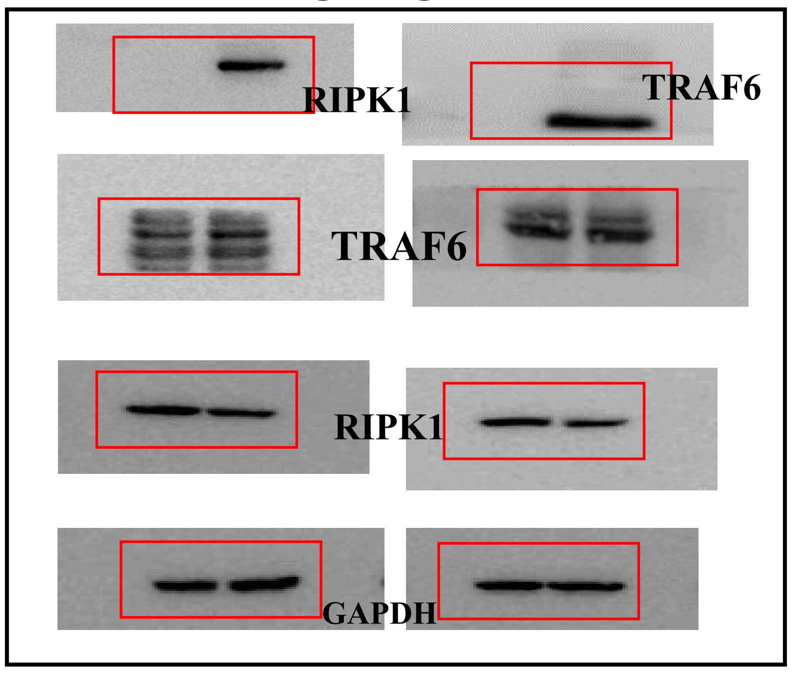

Fig. 2h

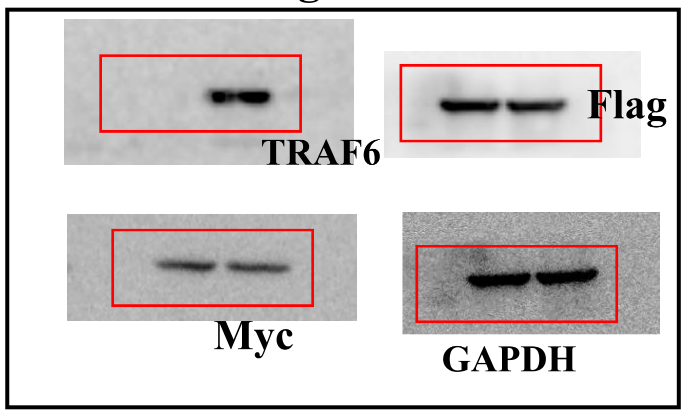

Fig. 2k

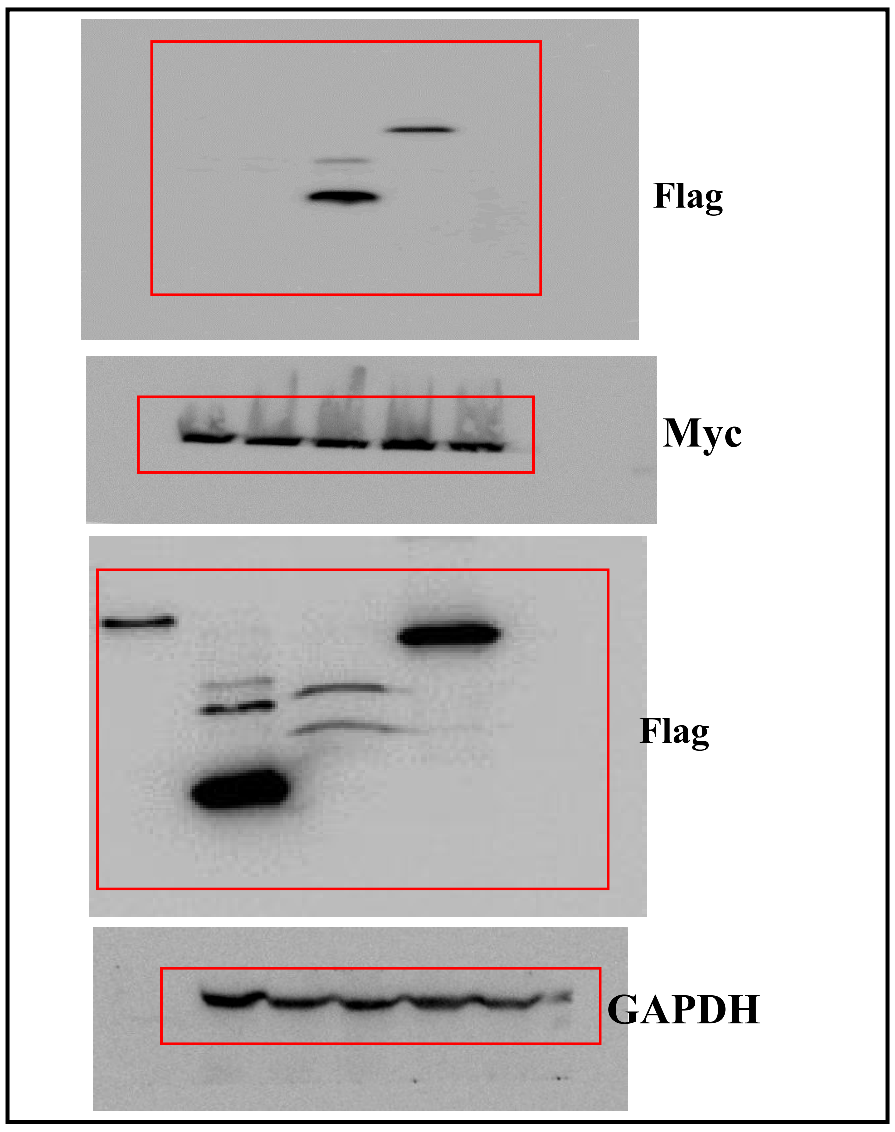

Fig. 2l

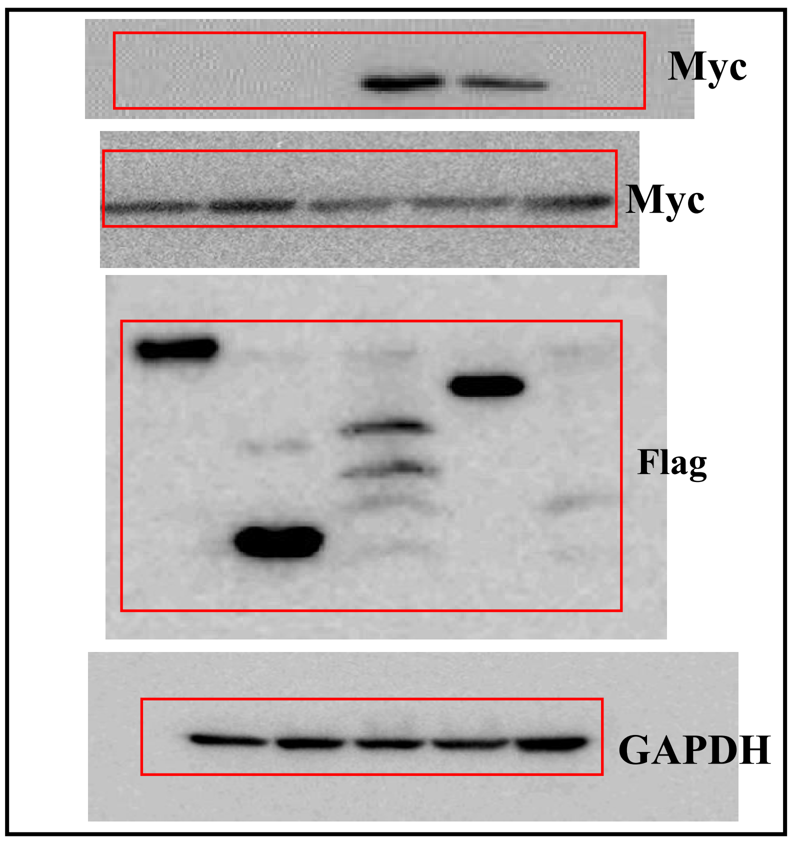

Fig. 2m

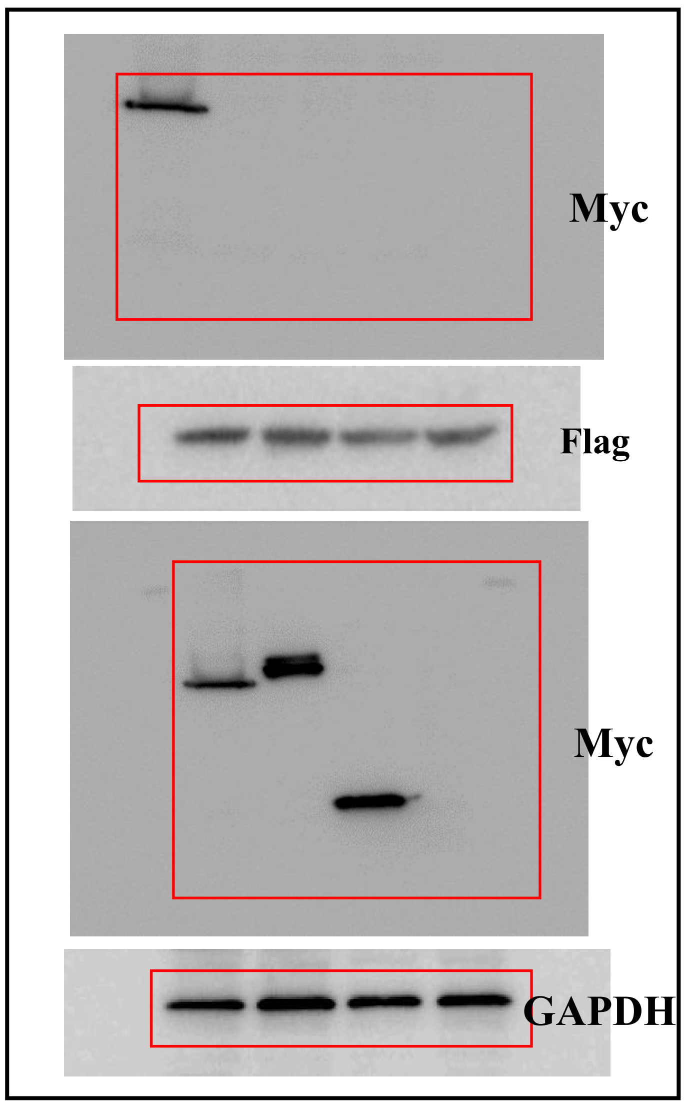

Fig. 2n

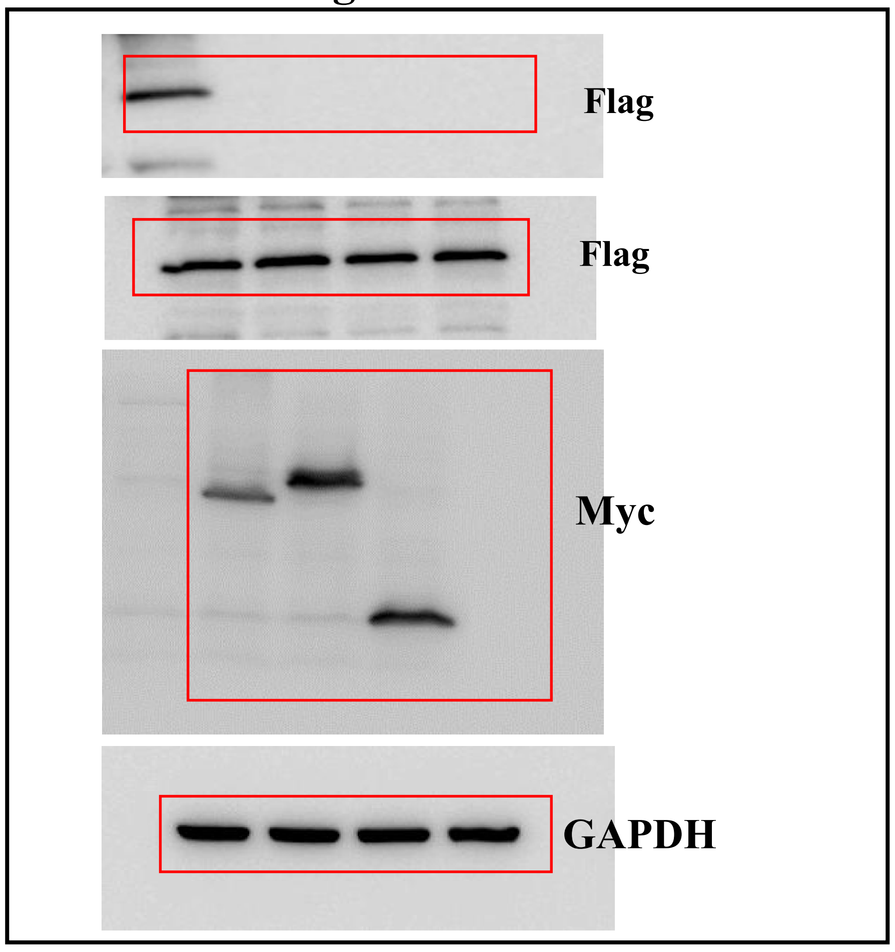

Fig. 2o

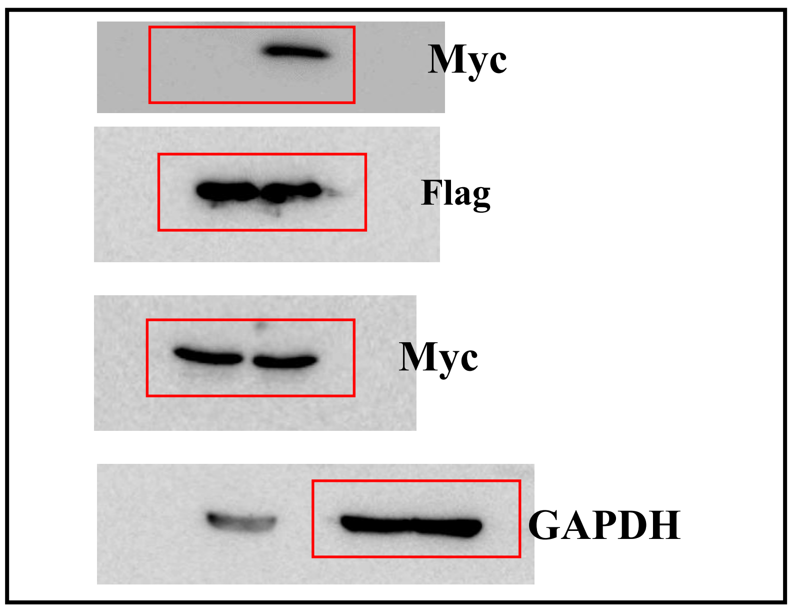

Uncropped blot images(page 2 of 6)

Fig. 3a/b

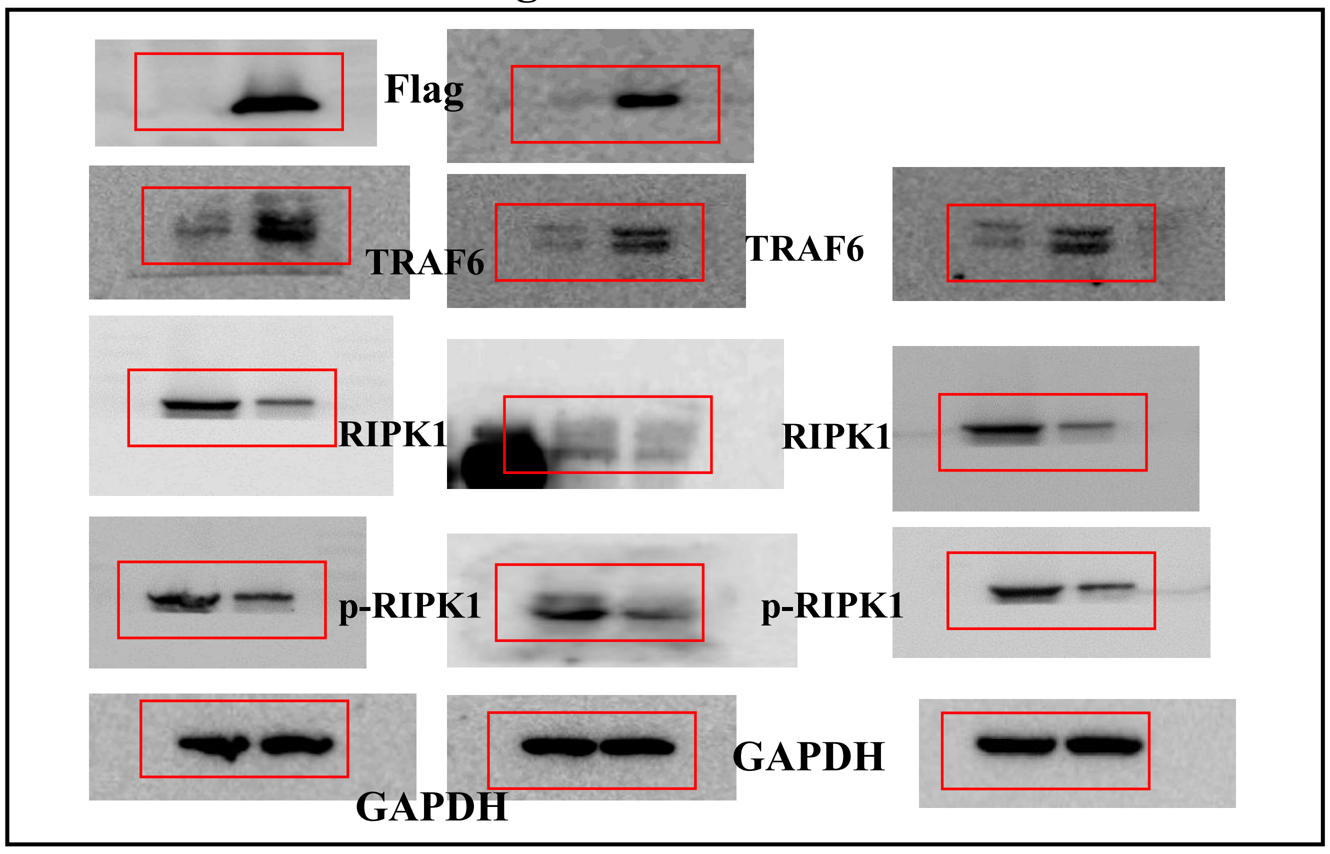

Fig. 3c

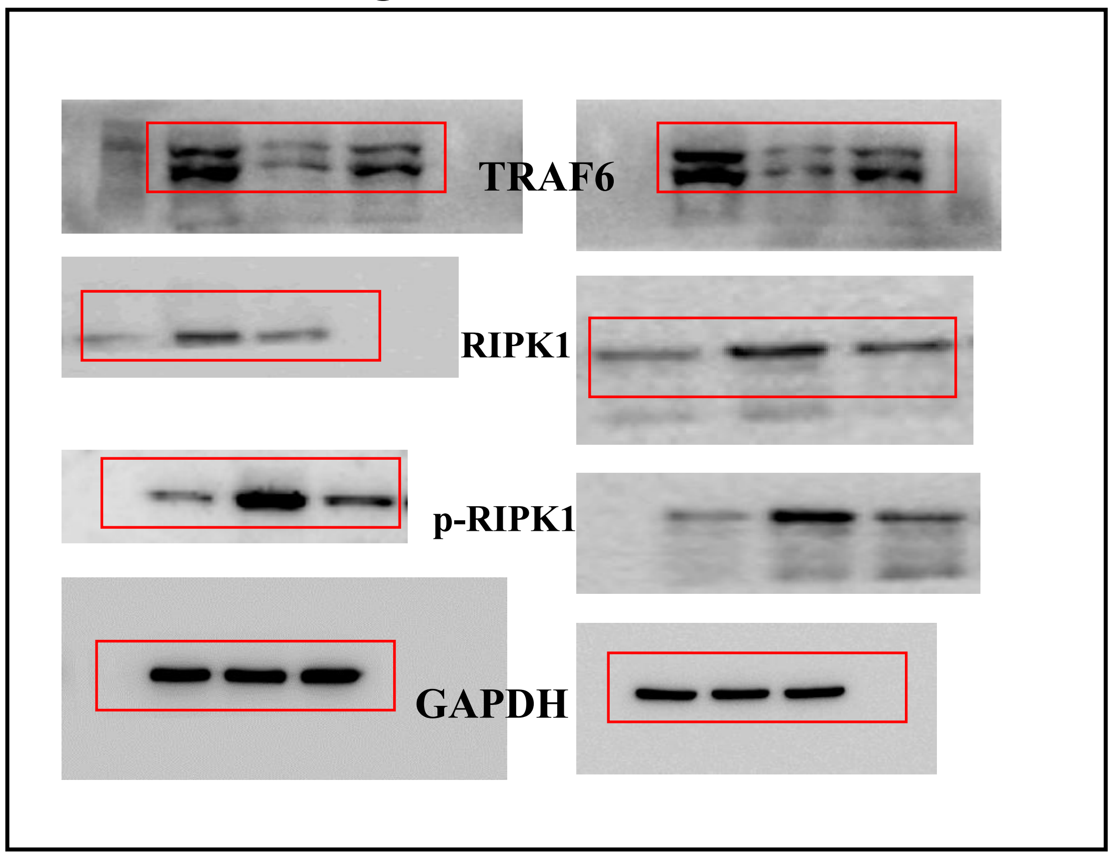

Fig. 3d

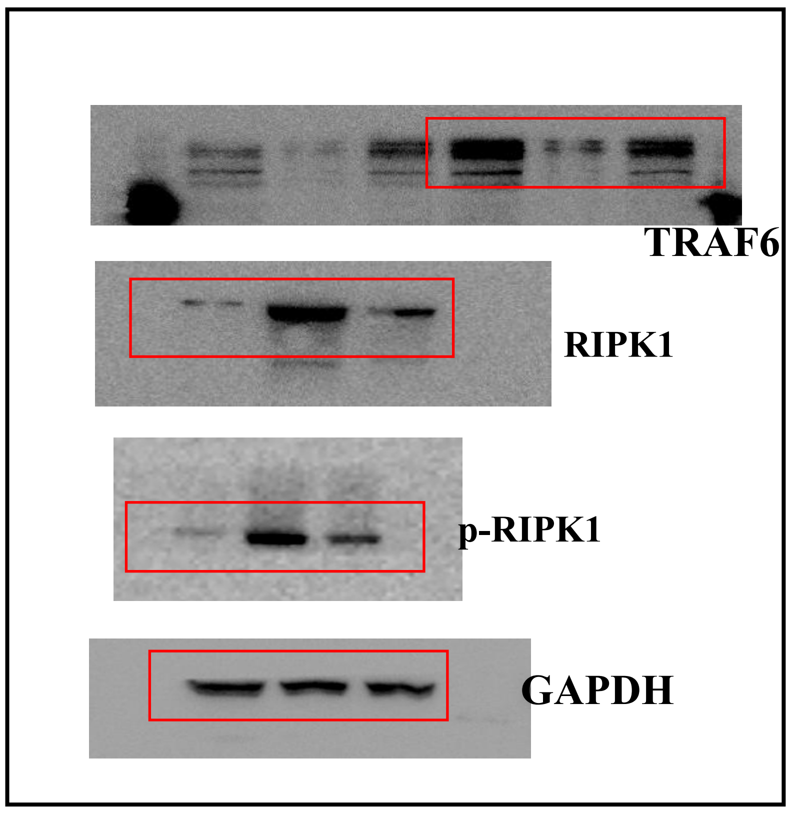

Fig. 3e

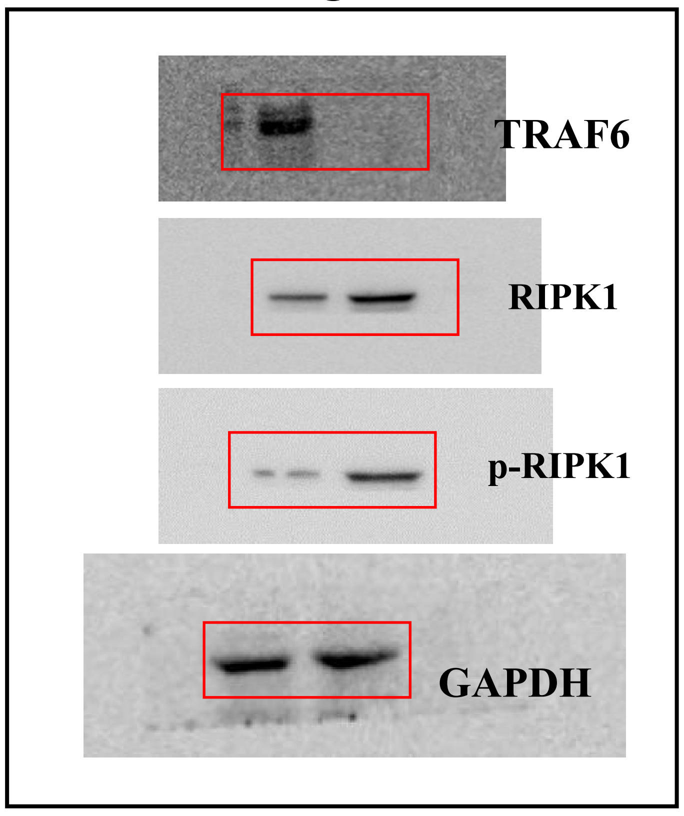

Fig. 3f

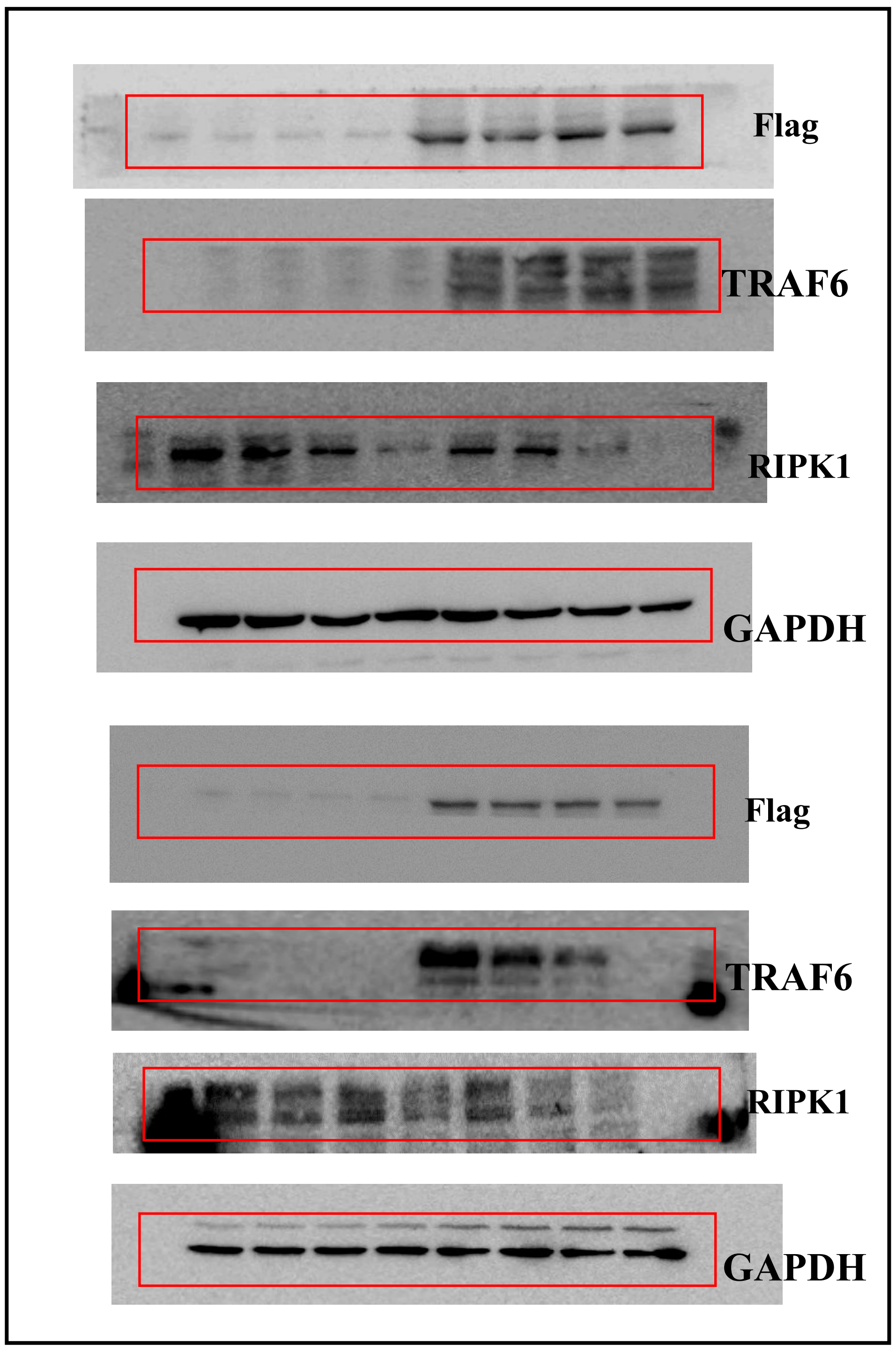

Fig. 3h

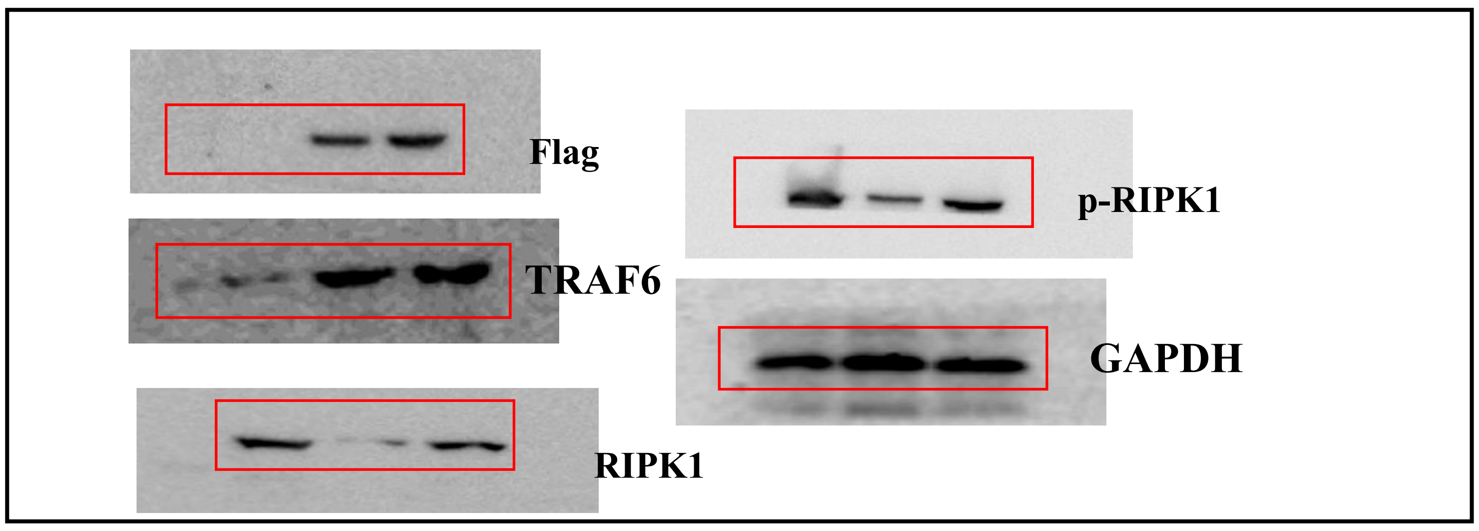

Fig. 3i

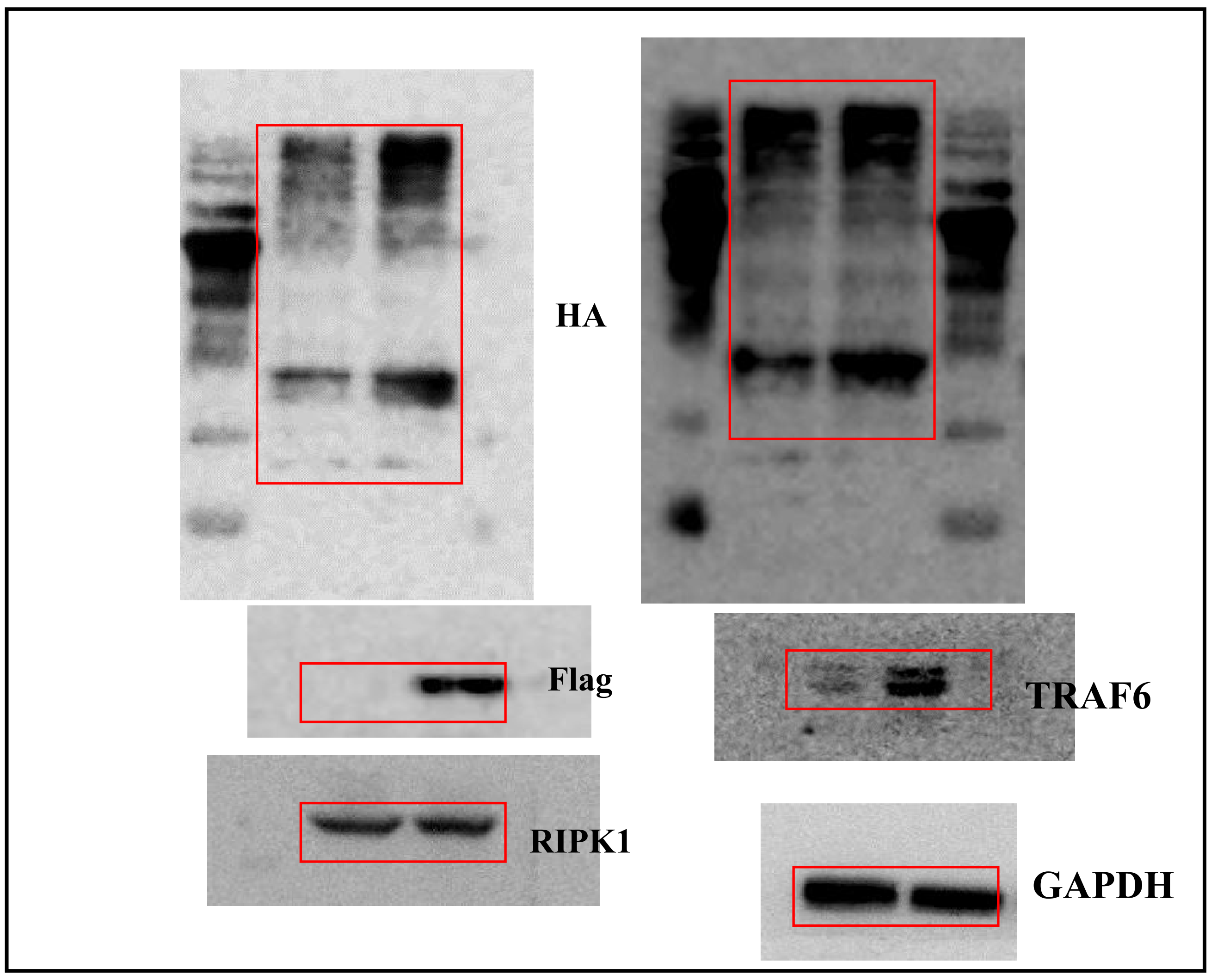

Fig. 3j

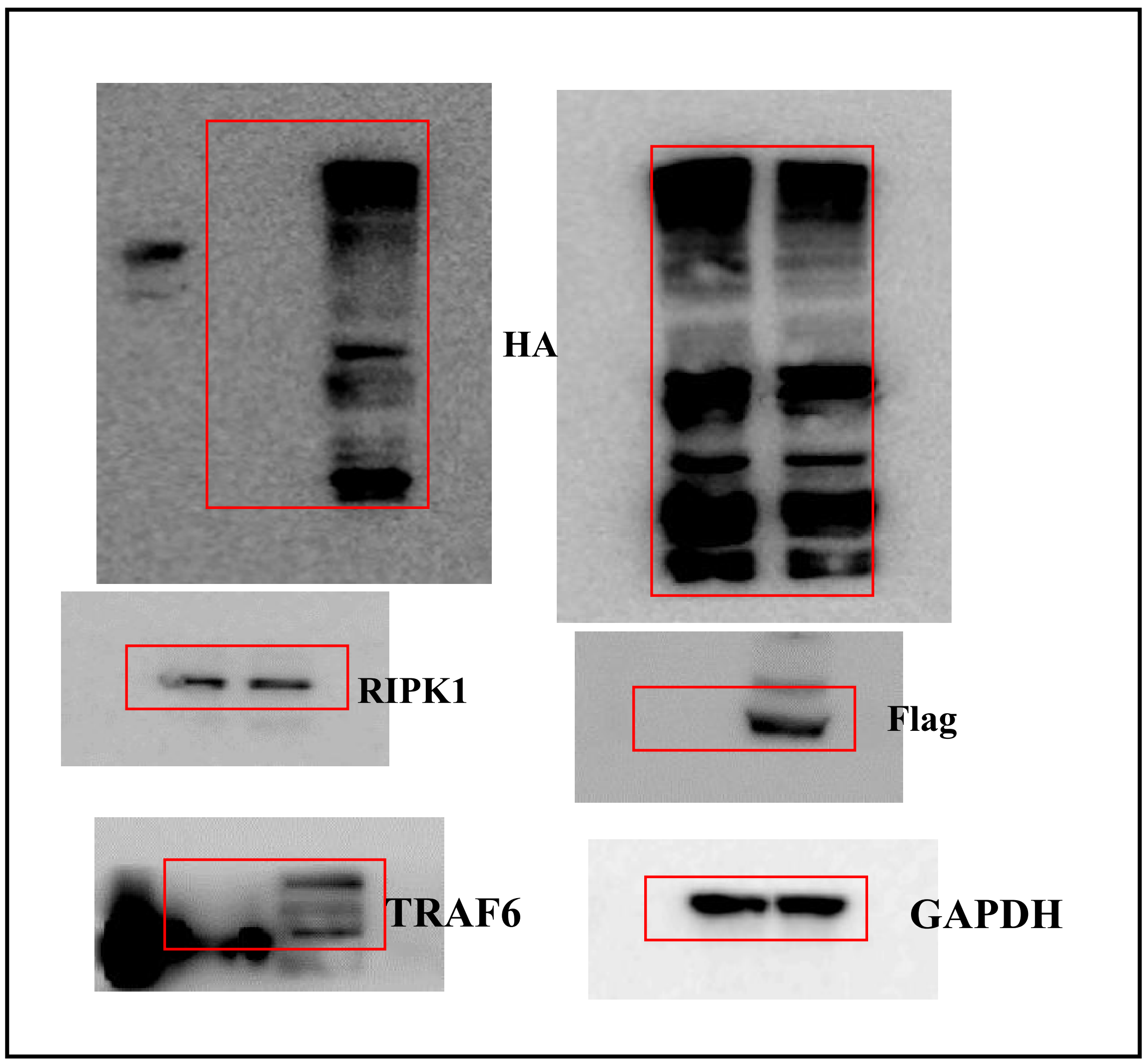

Uncropped blot images(page 3 of 6)

Fig. 3l/m/n

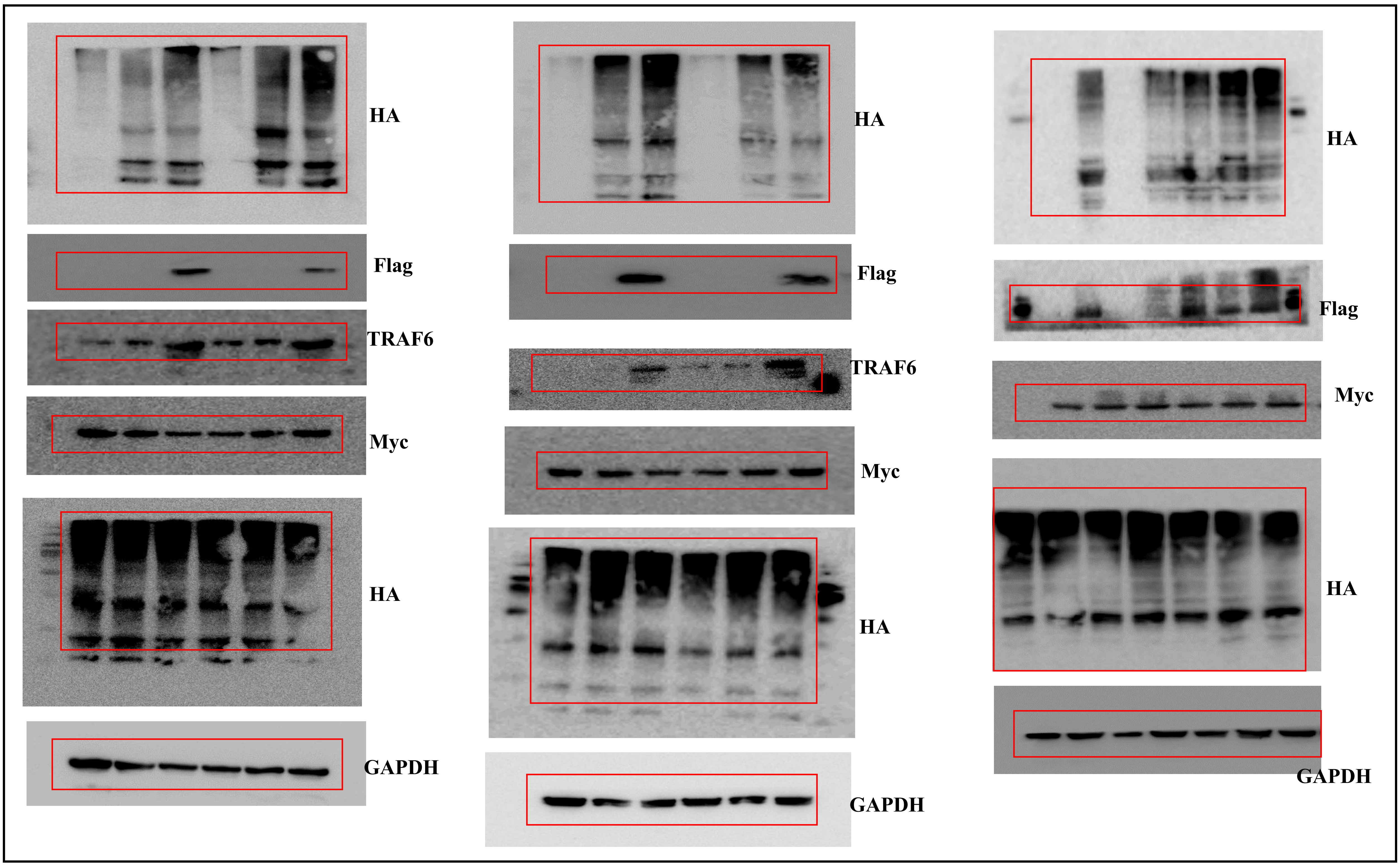

Fig. 4a

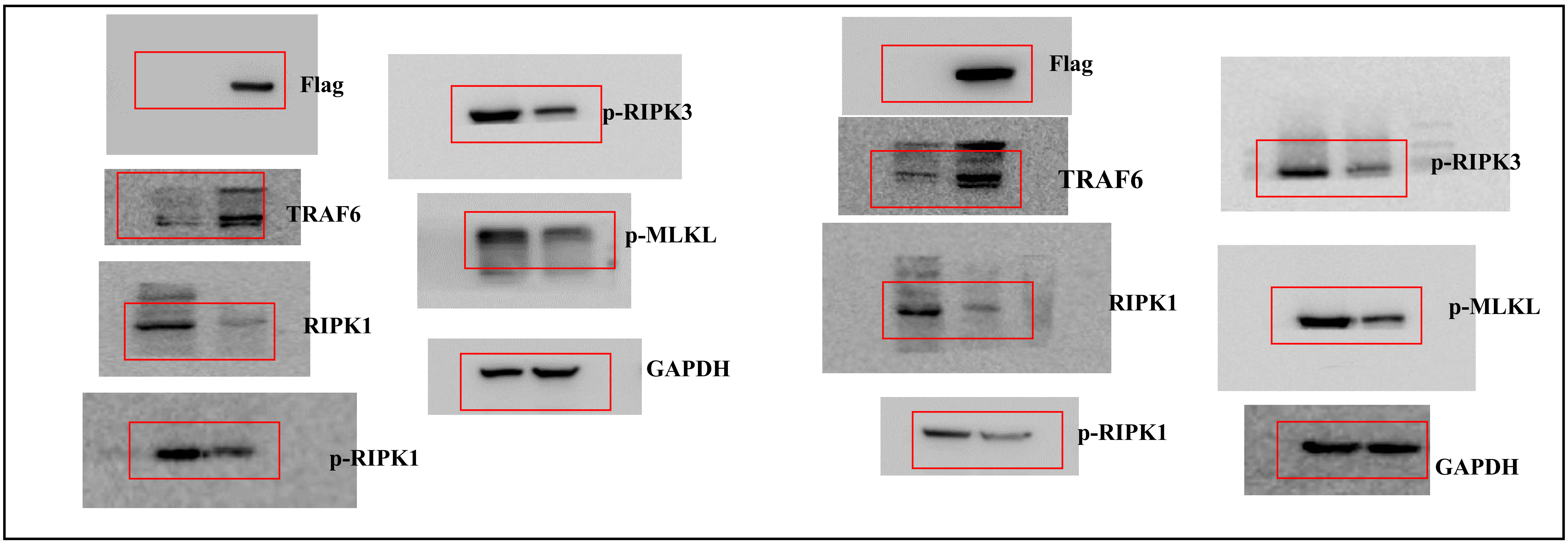

Fig. 4b

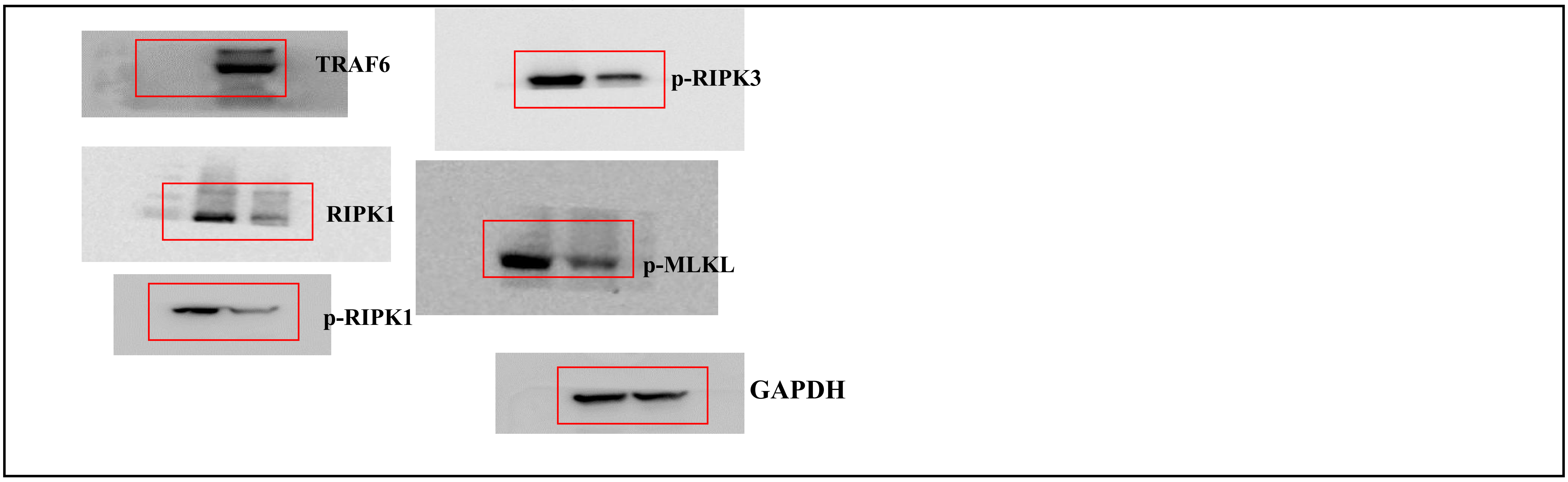

Fig. 4c

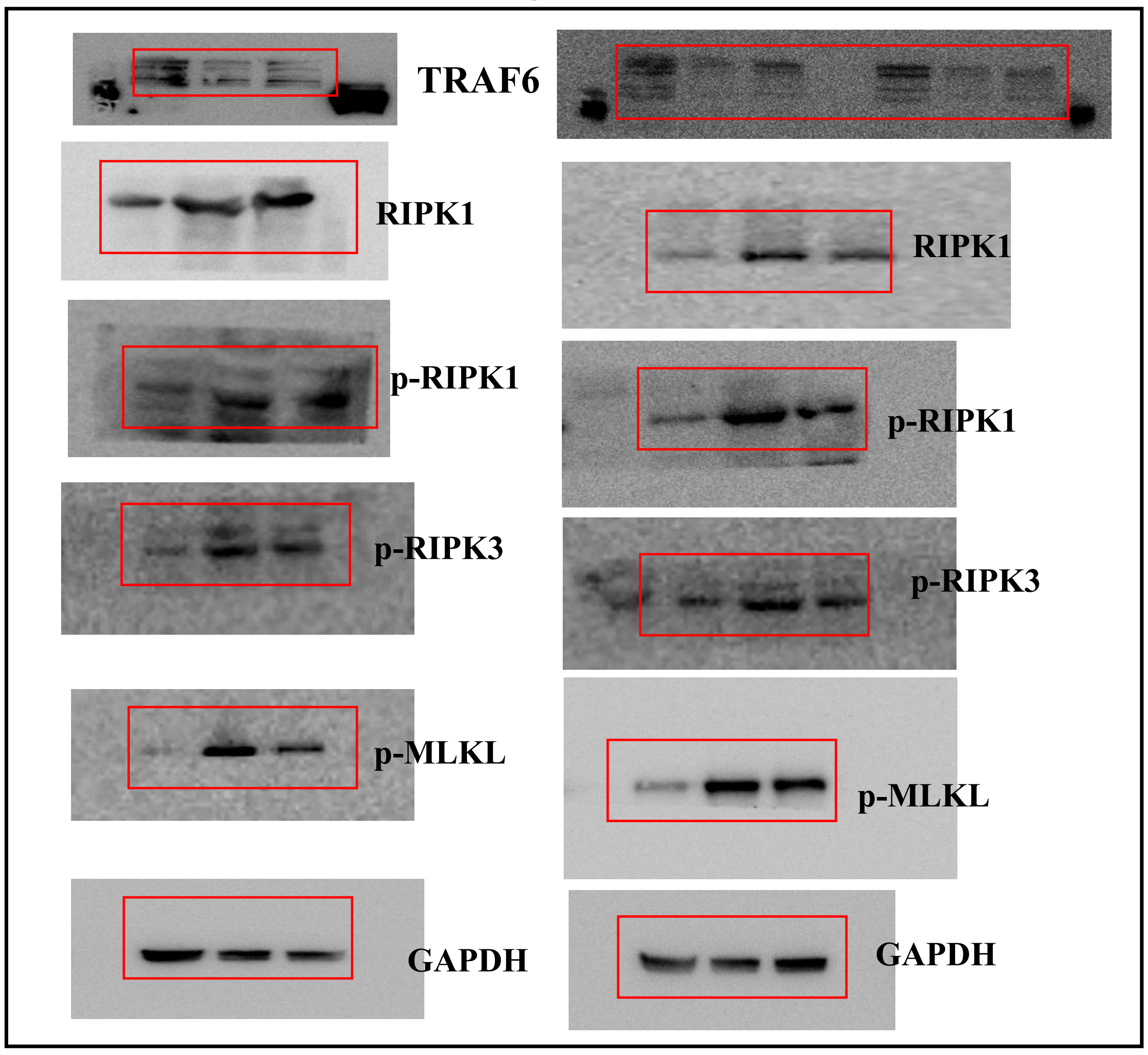

Fig. 4d

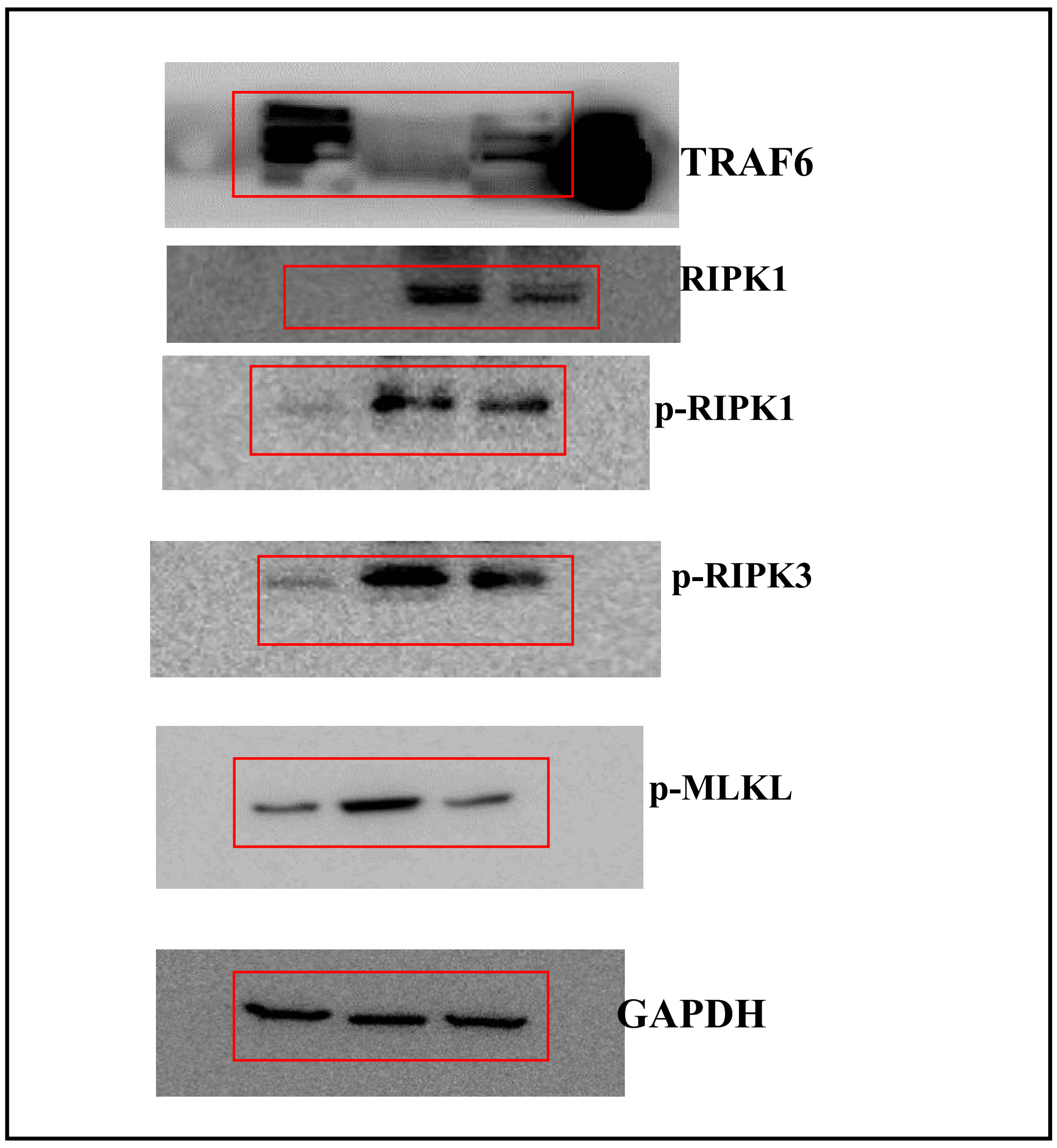

Fig. 4e/6a

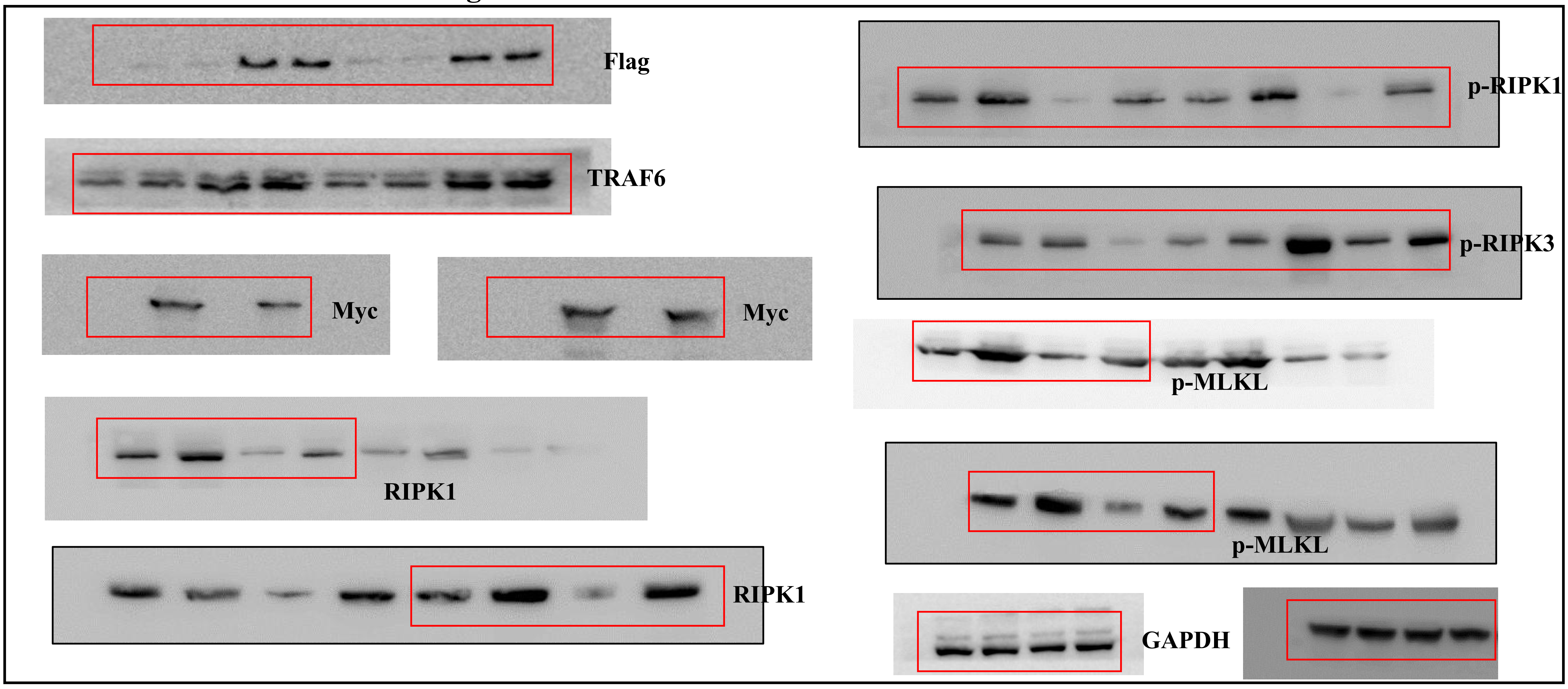

Fig. 4f

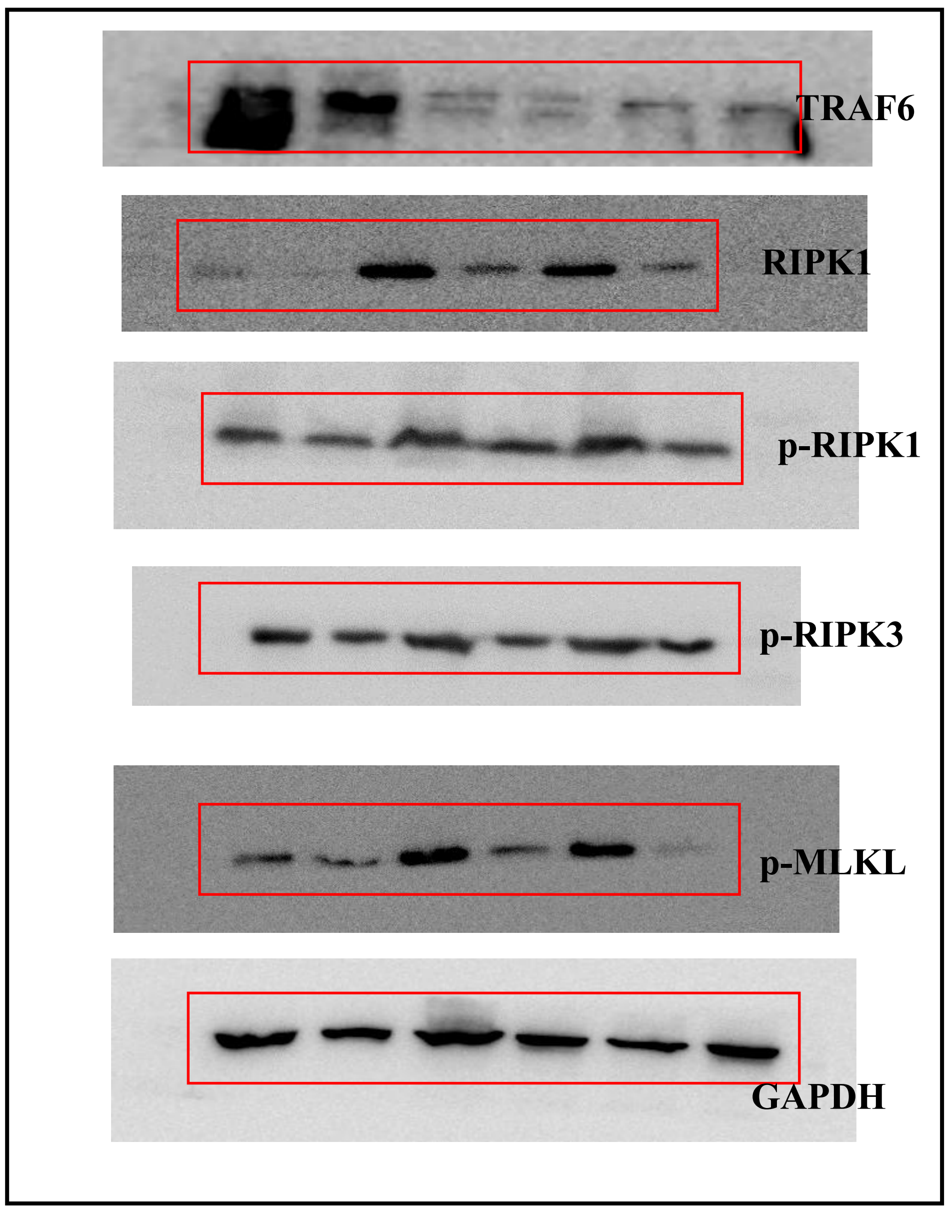

Fig. 5a

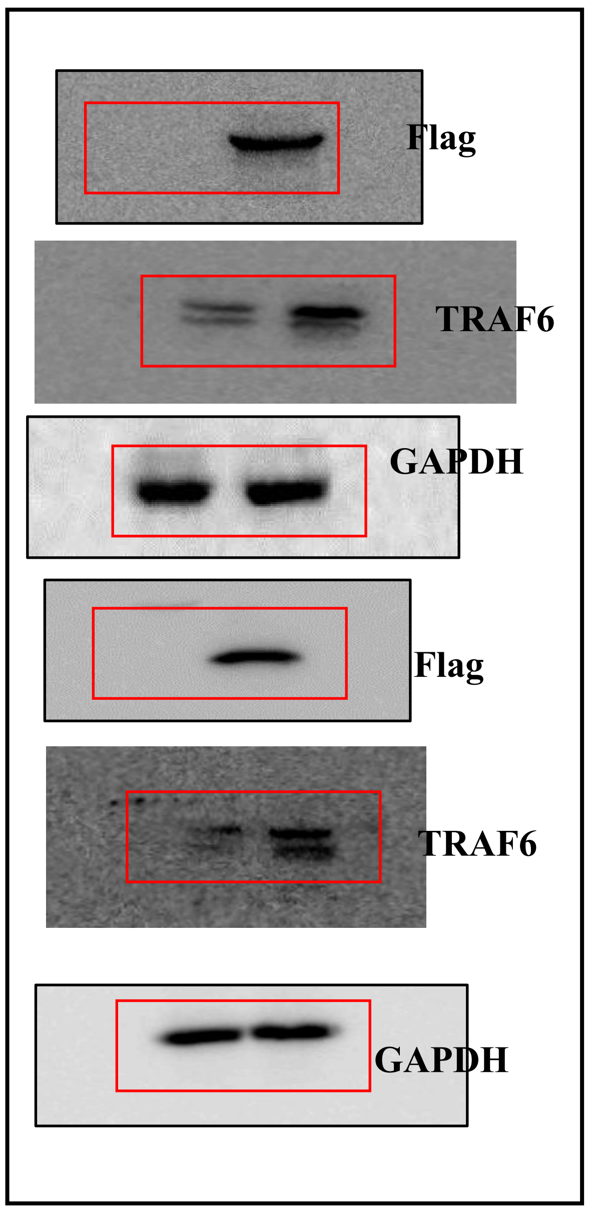

Fig. 5e

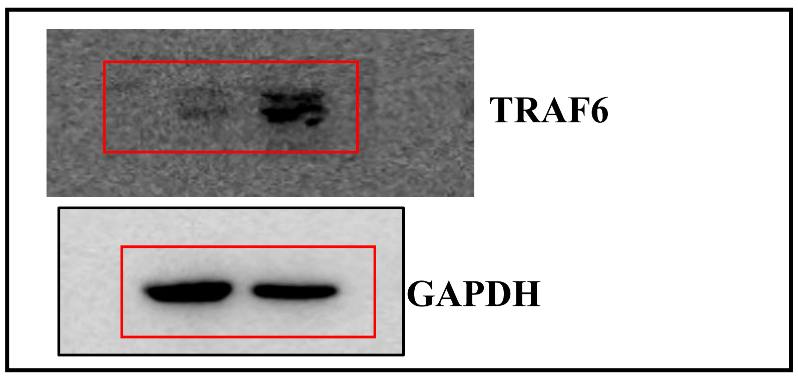

Fig. 5i

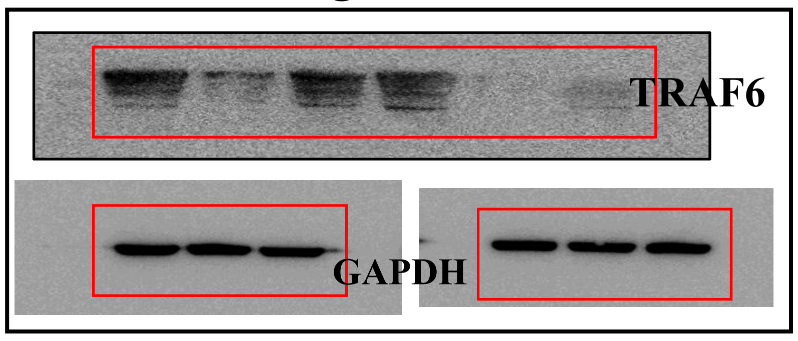

Fig. 5q

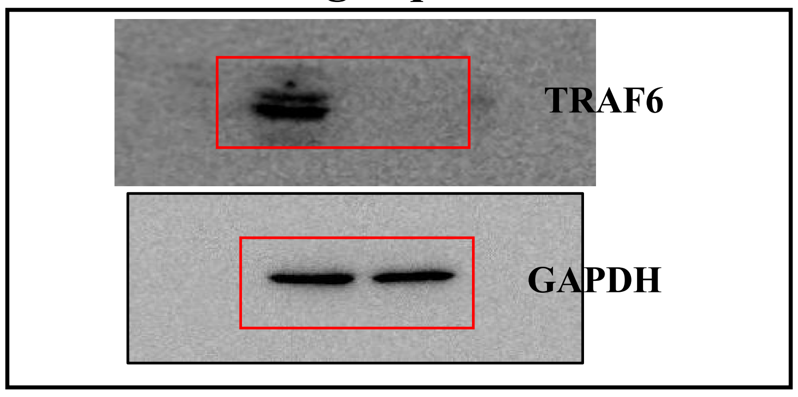

Fig. 5m

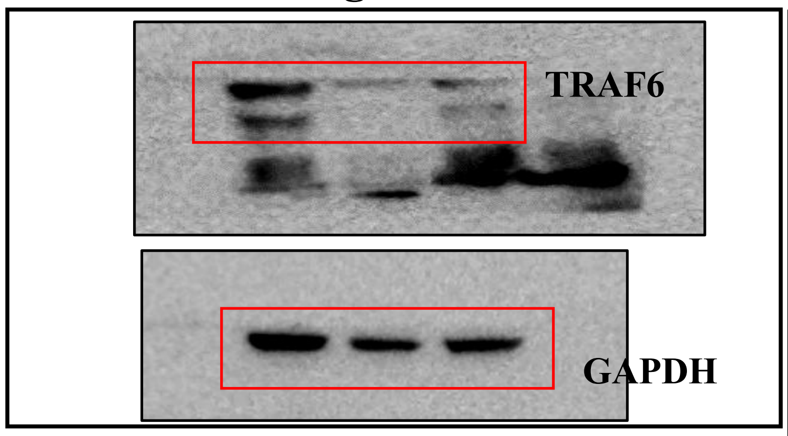

Fig. 6f

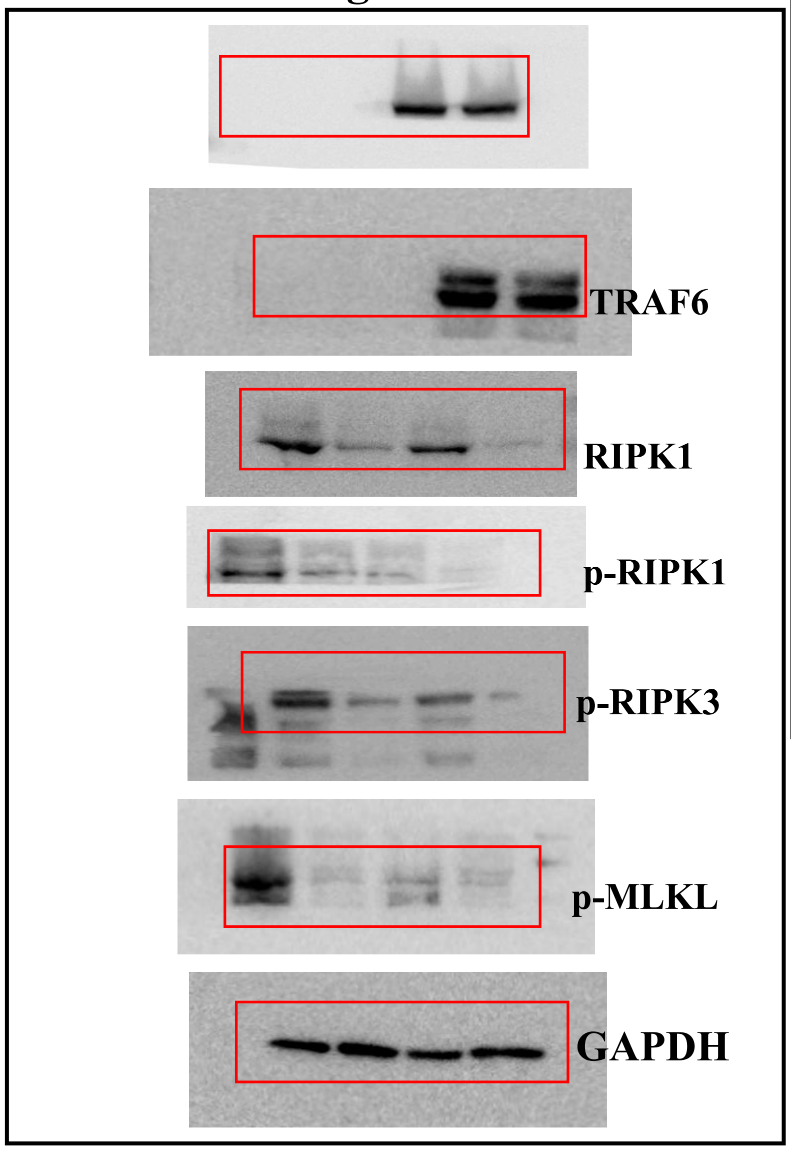

Fig. 6k

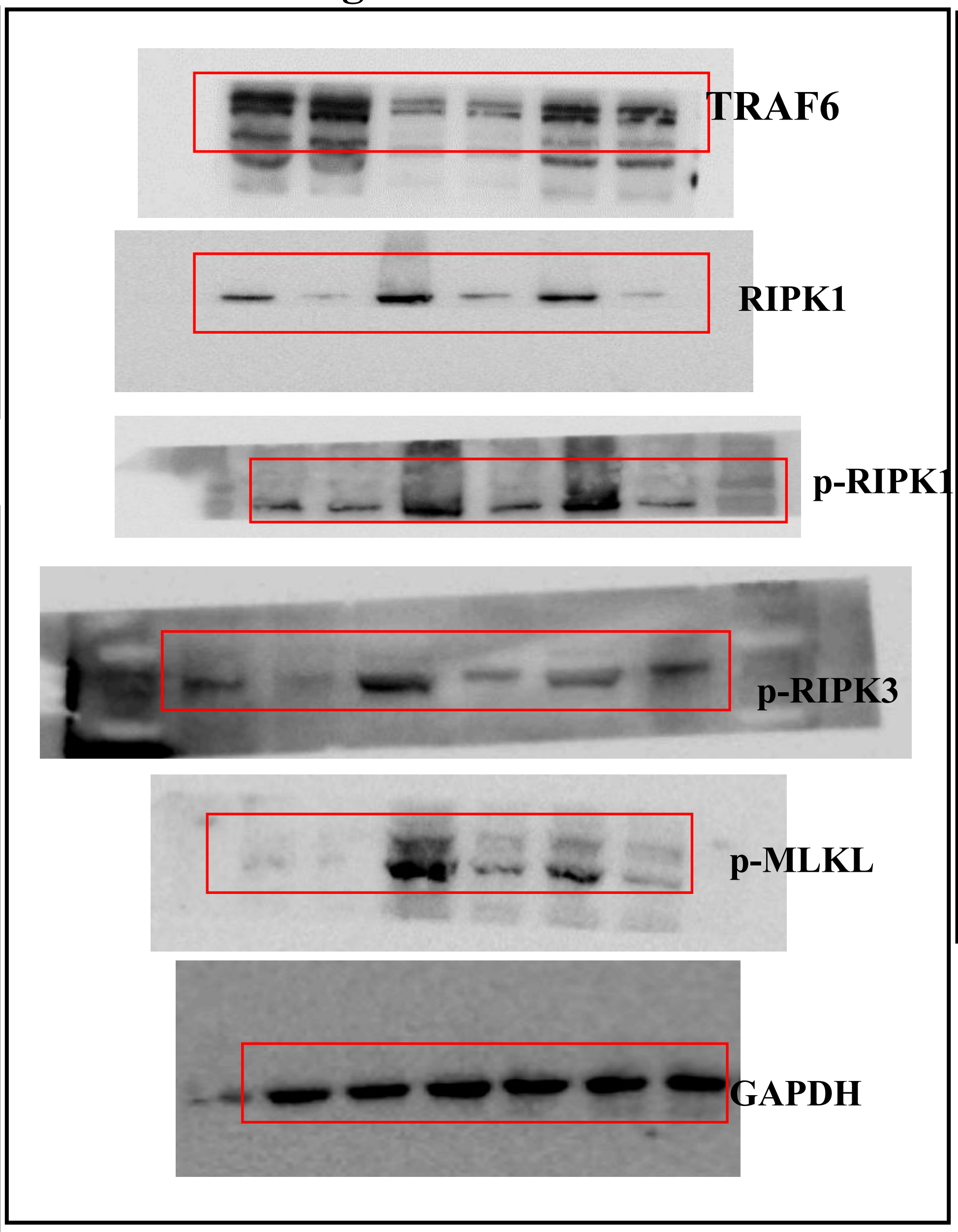

Fig. 7a

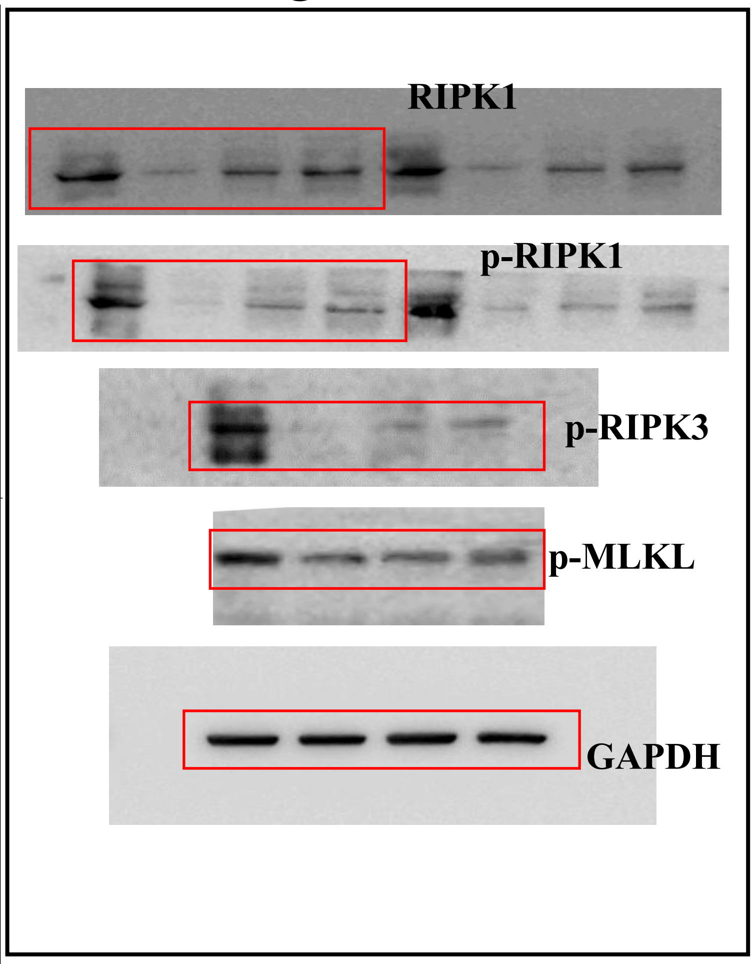

Fig. 8

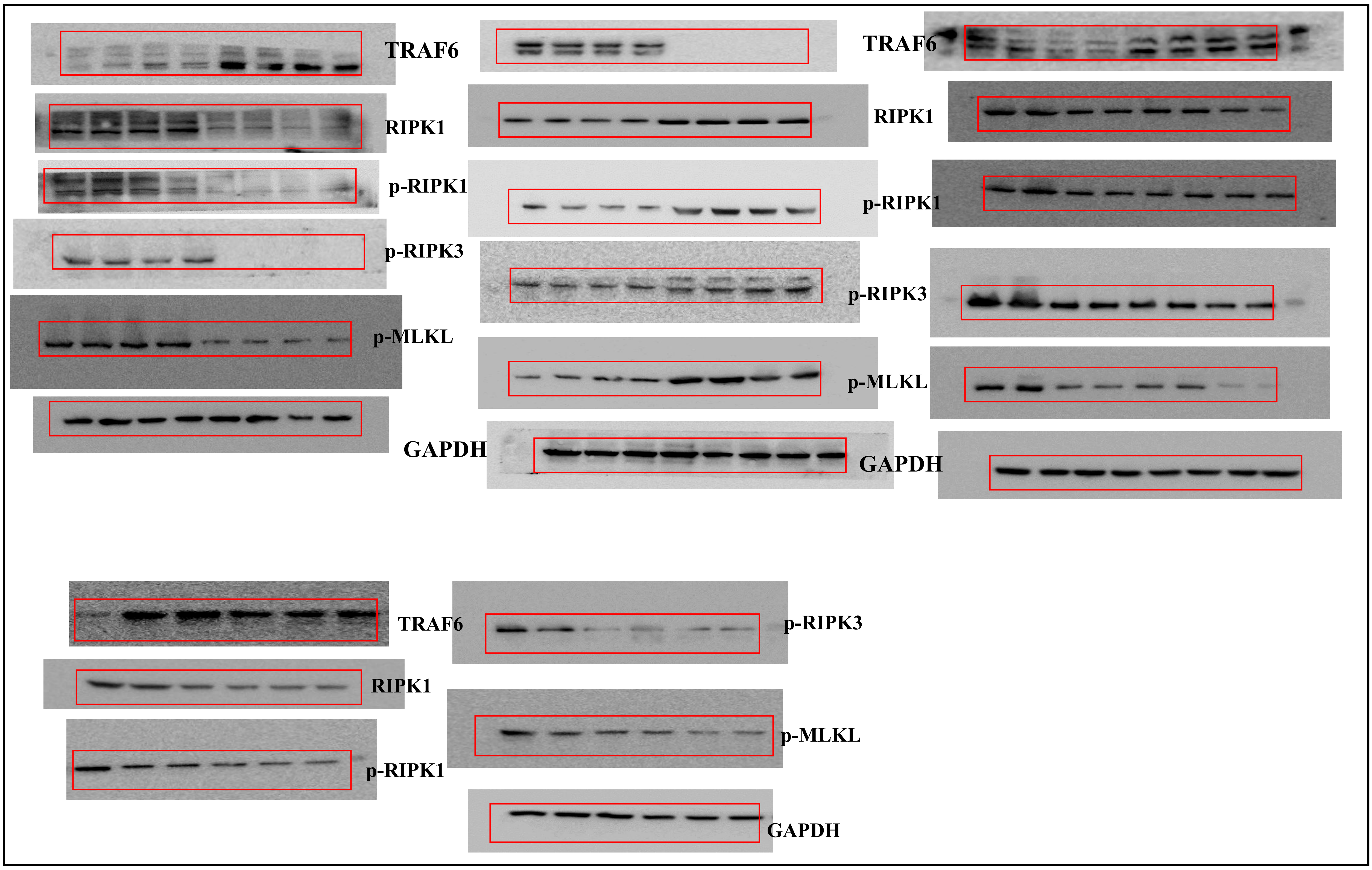

Uncropped blot images(page 6 of 6)

Fig. S1b

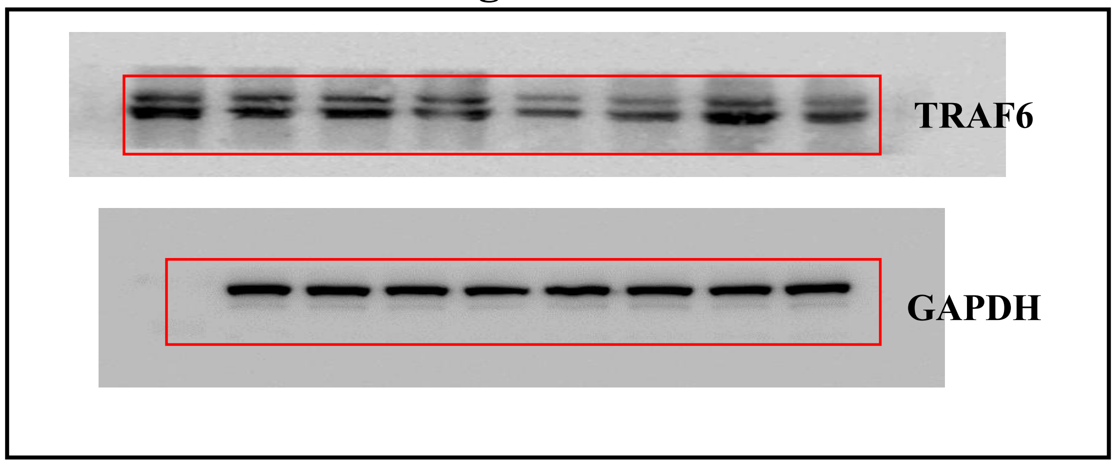

Fig. S4a/b

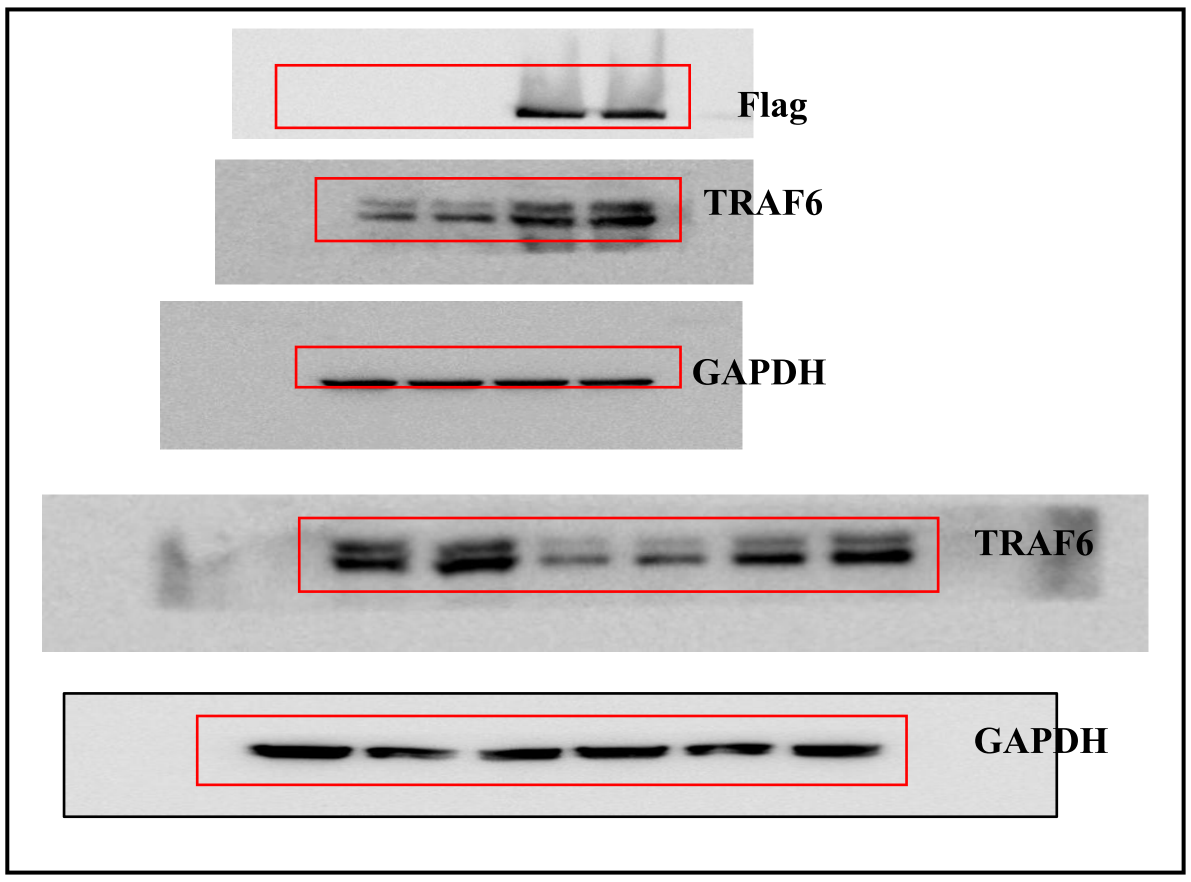

Fig. S4c/d

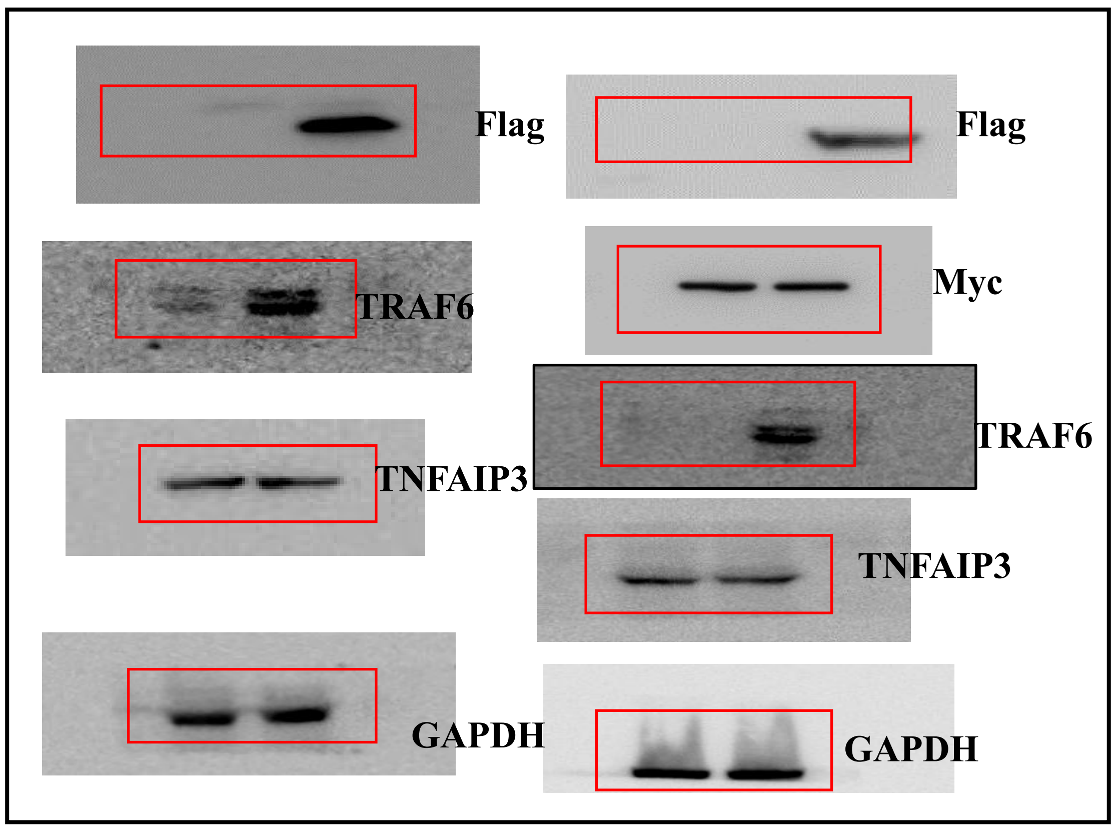

Fig. S5f

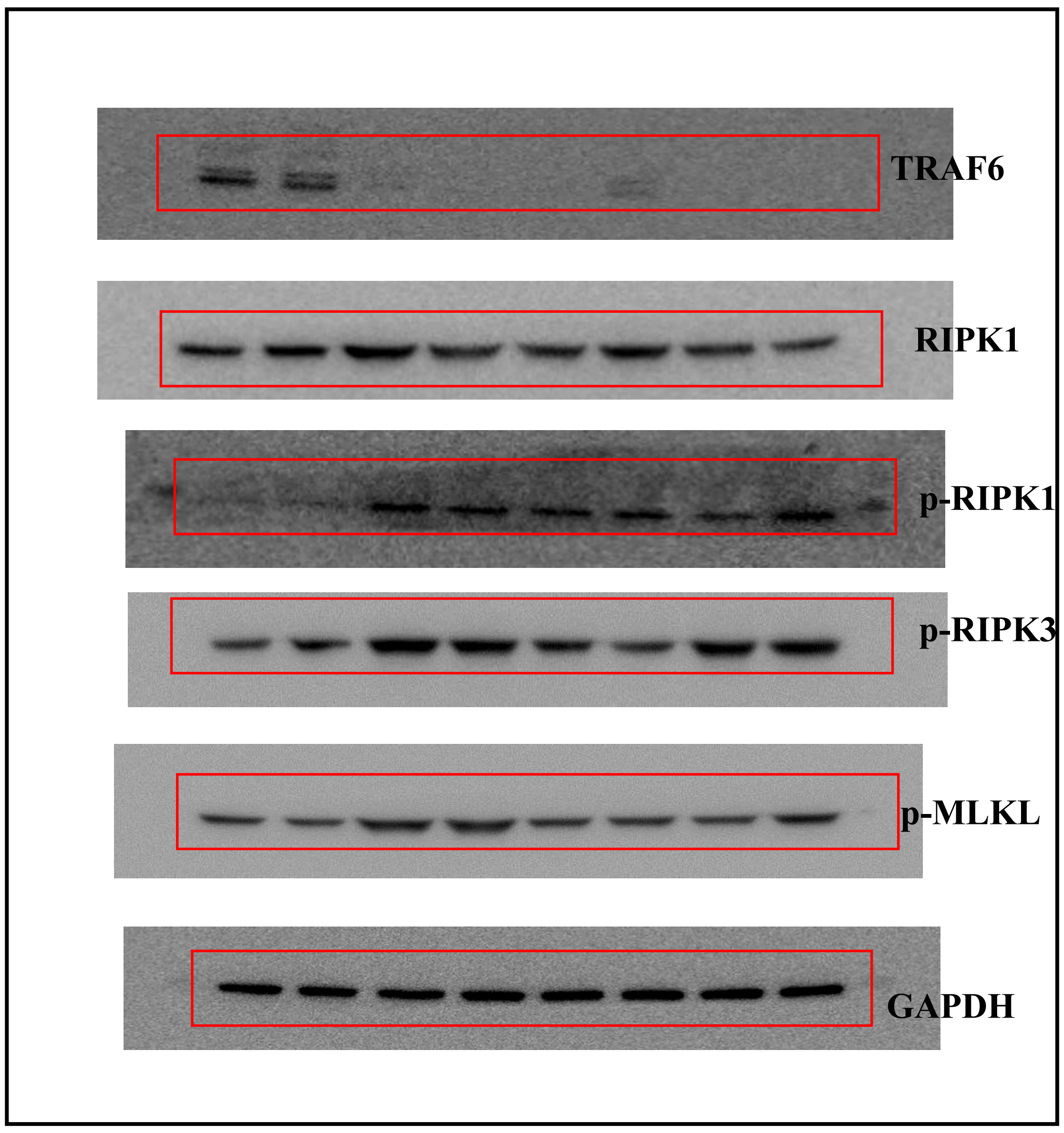

Supplement: Supplementary file 3 — Uncropped blot images [file 41419_2022_5524_MOESM3_ESM.pdf]
