# Supplementary material for: TRAF6 regulates the abundance of RIPK1 and inhibits the RIPK1/RIPK3/MLKL necroptosis signaling pathway and affects the progression of colorectal cancer
Source: Cell Death Dis. 2023 Jan 5;14(1):6. doi: 10.1038/s41419-022-05524-y (PMC9816173; doi:10.1038/s41419-022-05524-y)
Supplement: Supplementary file 4 — The patients agreement [file 41419_2022_5524_MOESM4_ESM.pdf]

Patient T1

临床研究知情同意书

尊敬的受试者：

您将被邀请参加一项由福建医科大学附属第一医院朱广伟（项目负责人）（PI 电话号码：18060473703）主持的研究，研究项目名称：TRAF6 调控 CREB/RIPK1-RIPK3-MLKL 信号通路抑制程序性坏死影响结直肠癌进展的机制研究。您参加本项研究是自愿的，本知情同意书提供给您一些信息以帮助您决定是否参加此项临床研究。本研究已通过福建医科大学附属第一医院伦理委员会审查同意，伦理委员会办公室电话 0591-87981029 。如果您同意加入此项研究，请您仔细阅读，如有任何疑问请向负责该项研究的研究者提出。

一、研究目的：

我们课题组在前面研究过程中发现，TRAF6 能够通过调控 NF-κB 和 AP-1 信号通路促进结直肠癌的进展。研究表明 TRAF6 能够调控肿瘤进展中的多个信号通路，处于肿瘤信号调控的核心位置，是肿瘤进展的关键限速节点。但是，TRAF6 能否程序性坏死通路影响结直肠癌的坏死，迄今未知。因此，本项目首先探讨 TRAF6 对结直肠癌坏死的影响；随后，进一步研究 TRAF6 是否通过 RIPK1-RIPK3-MLKL 信号通路调控结直肠癌的坏死；最后更加深入的研究 TRAF6 调控 RIPK1-RIPK3-MLKL 信号通路的确切机制。研究结果将进一步的揭示结直肠癌发展的机制，为抗结直肠癌复发转移理论研究和靶向药物设计提供新的思路。

二、研究过程和方法：

如果您同意参与这项研究，我们将对每位受试者进行编号，建立研究档案。在研究的过程中我们需要采集您手术后的组织标本，由专业人员在您手术后当日，选取手术后标本的癌组织和癌旁组织，大小各约 0.8cm*0.8cm。您的所有采集样本仅用于"TRAF6 调控 CREB/RIPK1-RIPK3-MLKL 信号通路抑制程序性坏死影响结直肠癌进展的机制研究"该实验的研究。如果您同意参与这项研究，我们将对每位受试者进行编号，建立研究档案。

三、研究可能的受益：

参与本研究将为结直肠癌疾病的研究提供有益的信息。

四、研究风险与不适：

本研究您无需承担任何费用及压力，也不会给您造成损伤。本研究将收集您的临床病理信息和剩余结直肠癌手术的组织标本，不会对您产生常规诊疗之外的风险与不适。您的血样品收集由专业人员严格按照无菌要求操作，采集过程可能出现一些并发症，包括局部血肿、疼痛、过敏以及少数人晕血晕针等。

五、隐私问题：

如果您决定参加本项研究，您参加试验及在试验中的个人资料均属保密。对于您来说，所有的信息将是保密的。您的标本将以研究编号数字而非您的姓名加以标识。可以识别您身份的信息将不会透露给研究小组以外的成员，除非获得您的许可。所有的研究成员和研究申办方都被要求对您的身份保密。您的档案将保存在福建医科大学附属第一医院 16 区胃肠外科档案柜中，仅供研究人员查阅。为确保研究按照规定进行，必要时，政府管理部门或伦理审查委员会的成员按规定可以在研究单位查阅您的个人资料。这项研究结果发表时，将不会披露您个人的任何资料。

六、费用：

参与本研究无常规诊疗之外费用。

七、补偿：

参与该研究无干预，没有风险和补偿。

八、自由退出：

作为受试者，您可随时了解与本研究有关的信息资料和研究进展，自愿决定（继续）参加还是不（继续）参加。参加后，无论是否发生伤害，您可以选择在任何时候通知研究者要求退出研究，您的数据将不纳入研究结果，您的任何医疗待遇与权益不会因此而受到影响。在试验过程中，若会对您造成严重的伤害，研究者也将会中止研究的进行。

但在参加研究期间，请您提供有关自身病史和当前身体状况的真实情况；告诉研究医生自己在本次研究期间所出现的任何不适；不得服用受限制的药物、食物等；告诉研究医生自己在最近是否曾参与其他研究，或目前正参与其他研究。如果因为您没有遵守研究计划，或者发生了与研究相关的损伤或者有任何其它原因，研究医师可以终止您继续参与本项研究。

九、联系方式：

如果您有与本研究有关的问题，或您在研究过程中发生了任何不适与损伤，或有关于本项研究参加者权益方面的问题，您可以与朱广伟医生联系，电话 18060473703。

十、知情同意签字：

我已经阅读了本知情同意书，并且我的医生 （签字）已经将此次临床试验的目的、内容、风险和受益情况向我作了详细的解释说明，对我询问的所有问题也给予了解答，我对此项临床研究已经了解，我自愿参加本项研究。

受试者签名：[illegible]
联系手机号：13025809697
日期：2018年 5 月 17 日

研究者签字：朱广伟
联系手机号：18060473703
日期：2018 年 5 月 17 日

（如果受试者不识字时尚需见证人签名，如果受试者无行为能力时则需代理人同意）

法定代理人/ 见证人签名：

与受试者的关系：

日期：______年_______月_______日

Patient T2

临床研究知情同意书

尊敬的受试者：

您将被邀请参加一项由福建医科大学附属第一医院朱广伟（项目负责人）（PI 电话号码：18060473703）主持的研究，研究项目名称：TRAF6 调控 CREB/RIPK1-RIPK3-MLKL 信号通路抑制程序性坏死影响结直肠癌进展的机制研究。您参加本项研究是自愿的，本知情同意书提供给您一些信息以帮助您决定是否参加此项临床研究。本研究已通过福建医科大学附属第一医院伦理委员会审查同意，伦理委员会办公室电话 0591-87981029 。如果您同意加入此项研究，请您仔细阅读，如有任何疑问请向负责该项研究的研究者提出。

一、研究目的：

我们课题组在前面研究过程中发现，TRAF6 能够通过调控 NF-κB 和 AP-1 信号通路促进结直肠癌的进展。研究表明 TRAF6 能够调控肿瘤进展中的多个信号通路，处于肿瘤信号调控的核心位置，是肿瘤进展的关键限速节点。但是，TRAF6 能否程序性坏死通路影响结直肠癌的坏死，迄今未知。因此，本项目首先探讨 TRAF6 对结直肠癌坏死的影响；随后，进一步研究 TRAF6 是否通过 RIPK1-RIPK3-MLKL 信号通路调控结直肠癌的坏死；最后更加深入的研究 TRAF6 调控 RIPK1-RIPK3-MLKL 信号通路的确切机制。研究结果将进一步的揭示结直肠癌发展的机制，为抗结直肠癌复发转移理论研究和靶向药物设计提供新的思路。

二、研究过程和方法：

如果您同意参与这项研究，我们将对每位受试者进行编号，建立研究档案。在研究的过程中我们需要采集您手术后的组织标本，由专业人员在您手术后当日，选取手术后标本的癌组织和癌旁组织，大小各约 0.8cm*0.8cm。您的所有采集样本仅用于“TRAF6 调控 CREB/RIPK1-RIPK3-MLKL 信号通路抑制程序性坏死影响结直肠癌进展的机制研究”该实验的研究。如果您同意参与这项研究，我们将对每位受试者进行编号，建立研究档案。

三、研究可能的受益：

参与本研究将为结直肠癌疾病的研究提供有益的信息。

四、研究风险与不适：

本研究您无需承担任何费用及压力，也不会给您造成损伤。本研究将收集您的临床病理信息和剩余结直肠癌手术的组织标本，不会对您产生常规诊疗之外的风险与不适。您的血样品收集由专业人员严格按照无菌要求操作，采集过程可能出现一些并发症，包括局部血肿、疼痛、过敏以及少数人晕血晕针等。

五、隐私问题：

如果您决定参加本项研究，您参加试验及在试验中的个人资料均属保密。对于您来说，所有的信息将是保密的。您的标本将以研究编号数字而非您的姓名加以标识。可以识别您身份的信息将不会透露给研究小组以外的成员，除非获得您的许可。所有的研究成员和研究申办方都被要求对您的身份保密。您的档案将保存在福建医科大学附属第一医院 16 区胃肠外科档案柜中，仅供研究人员查阅。为确保研究按照规定进行，必要时，政府管理部门或伦理审查委员会的成员按规定可以在研究单位查阅您的个人资料。这项研究结果发表时，将不会披露您个人的任何资料。

六、费用：

参与本研究无常规诊疗之外费用。

七、补偿：

参与该研究无干预，没有风险和补偿。

八、自由退出：

作为受试者，您可随时了解与本研究有关的信息资料和研究进展，自愿决定（继续）参加还是不（继续）参加。参加后，无论是否发生伤害，您可以选择在任何时候通知研究者要求退出研究，您的数据将不纳入研究结果，您的任何医疗待遇与权益不会因此而受到影响。在试验过程中，若会对您造成严重的伤害，研究者也将会中止研究的进行。

但在参加研究期间，请您提供有关自身病史和当前身体状况的真实情况；告诉研究医生自己在本次研究期间所出现的任何不适；不得服用受限制的药物、食物等；告诉研究医生自己在最近是否曾参与其他研究，或目前正参与其他研究。如果因为您没有遵守研究计划，或者发生了与研究相关的损伤或者有任何其它原因，研究医师可以终止您继续参与本项研究。

九、联系方式：

如果您有与本研究有关的问题，或您在研究过程中发生了任何不适与损伤，或有关于本项研究参加者权益方面的问题，您可以与朱广伟医生联系，电话 18060473703。

十、知情同意签字：

我已经阅读了本知情同意书，并且我的医生 （签字）已经将此次临床试验的目的、内容、风险和受益情况向我作了详细的解释说明，对我询问的所有问题也给予了解答，我对此项临床研究已经了解，我自愿参加本项研究。

受试者签名：林炯贵
联系手机号：15059492656
日期：2018年 5 月 12 日

研究者签字：朱广伟
联系手机号：18060473703
日期：2018年 5 月 12 日

（如果受试者不识字时尚需见证人签名，如果受试者无行为能力时则需代理人同意）

法定代理人/ 见证人签名：

与受试者的关系：

日期：______年_______月_______日

Patient T3

临床研究知情同意书

尊敬的受试者：

您将被邀请参加一项由福建医科大学附属第一医院朱广伟（项目负责人）（PI 电话号码：18060473703）主持的研究，研究项目名称：TRAF6 调控 CREB/RIPK1-RIPK3-MLKL 信号通路抑制程序性坏死影响结直肠癌进展的机制研究。您参加本项研究是自愿的，本知情同意书提供给您一些信息以帮助您决定是否参加此项临床研究。本研究已通过福建医科大学附属第一医院伦理委员会审查同意，伦理委员会办公室电话 0591-87981029 。如果您同意加入此项研究，请您仔细阅读，如有任何疑问请向负责该项研究的研究者提出。

一、研究目的：

我们课题组在前面研究过程中发现，TRAF6 能够通过调控 NF-κB 和 AP-1 信号通路促进结直肠癌的进展。研究表明 TRAF6 能够调控肿瘤进展中的多个信号通路，处于肿瘤信号调控的核心位置，是肿瘤进展的关键限速节点。但是，TRAF6 能否程序性坏死通路影响结直肠癌的坏死，迄今未知。因此，本项目首先探讨 TRAF6 对结直肠癌坏死的影响；随后，进一步研究 TRAF6 是否通过 RIPK1-RIPK3-MLKL 信号通路调控结直肠癌的坏死；最后更加深入的研究 TRAF6 调控 RIPK1-RIPK3-MLKL 信号通路的确切机制。研究结果将进一步的揭示结直肠癌发展的机制，为抗结直肠癌复发转移理论研究和靶向药物设计提供新的思路。

二、研究过程和方法：

如果您同意参与这项研究，我们将对每位受试者进行编号，建立研究档案。在研究的过程中我们需要采集您手术后的组织标本，由专业人员在您手术后当日，选取手术后标本的癌组织和癌旁组织，大小各约 0.8cm*0.8cm。您的所有采集样本仅用于"TRAF6 调控 CREB/RIPK1-RIPK3-MLKL 信号通路抑制程序性坏死影响结直肠癌进展的机制研究"该实验的研究。如果您同意参与这项研究，我们将对每位受试者进行编号，建立研究档案。

三、研究可能的受益：

参与本研究将为结直肠癌疾病的研究提供有益的信息。

四、研究风险与不适：

本研究您无需承担任何费用及压力，也不会给您造成损伤。本研究将收集您的临床病理信息和剩余结直肠癌手术的组织标本，不会对您产生常规诊疗之外的风险与不适。您的血样品收集由专业人员严格按照无菌要求操作，采集过程可能出现一些并发症，包括局部血肿、疼痛、过敏以及少数人晕血晕针等。

五、隐私问题：

如果您决定参加本项研究，您参加试验及在试验中的个人资料均属保密。对于您来说，所有的信息将是保密的。您的标本将以研究编号数字而非您的姓名加以标识。可以识别您身份的信息将不会透露给研究小组以外的成员，除非获得您的许可。所有的研究成员和研究申办方都被要求对您的身份保密。您的档案将保存在福建医科大学附属第一医院 16 区胃肠外科档案柜中，仅供研究人员查阅。为确保研究按照规定进行，必要时，政府管理部门或伦理审查委员会的成员按规定可以在研究单位查阅您的个人资料。这项研究结果发表时，将不会披露您个人的任何资料。

六、费用：

参与本研究无常规诊疗之外费用。

七、补偿：

版本号：1.0 版本日期：2018 年 3 月 8 日

参与该研究无干预，没有风险和补偿。

八、自由退出：

作为受试者，您可随时了解与本研究有关的信息资料和研究进展，自愿决定（继续）参加还是不（继续）参加。参加后，无论是否发生伤害，您可以选择在任何时候通知研究者要求退出研究，您的数据将不纳入研究结果，您的任何医疗待遇与权益不会因此而受到影响。在试验过程中，若会对您造成严重的伤害，研究者也将会中止研究的进行。

但在参加研究期间，请您提供有关自身病史和当前身体状况的真实情况；告诉研究医生自己在本次研究期间所出现的任何不适；不得服用受限制的药物、食物等；告诉研究医生自己在最近是否曾参与其他研究，或目前正参与其他研究。如果因为您没有遵守研究计划，或者发生了与研究相关的损伤或者有任何其它原因，研究医师可以终止您继续参与本项研究。

九、联系方式：

如果您有与本研究有关的问题，或您在研究过程中发生了任何不适与损伤，或有关于本项研究参加者权益方面的问题，您可以与朱广伟医生联系，电话 18060473703。

十、知情同意签字：

我已经阅读了本知情同意书，并且我的医生 （签字）已经将此次临床试验的目的、内容、风险和受益情况向我作了详细的解释说明，对我询问的所有问题也给予了解答，我对此项临床研究已经了解，我自愿参加本项研究。

受试者签名：[illegible]
联系手机号：[illegible]6966
日期：2018 年 5 月 12 日

研究者签字：朱广伟
联系手机号：18060473703
日期：2018 年 5 月 12 日

（如果受试者不识字时尚需见证人签名，如果受试者无行为能力时则需代理人同意）

法定代理人/ 见证人签名：

与受试者的关系：

日期：_____ 年 ______ 月 ______ 日

Patient T4

临床研究知情同意书

尊敬的受试者：

您将被邀请参加一项由福建医科大学附属第一医院朱广伟（项目负责人）（PI 电话号码：18060473703）主持的研究，研究项目名称：TRAF6 调控 CREB/RIPK1-RIPK3-MLKL 信号通路抑制程序性坏死影响结直肠癌进展的机制研究。您参加本项研究是自愿的，本知情同意书提供给您一些信息以帮助您决定是否参加此项临床研究。本研究已通过福建医科大学附属第一医院伦理委员会审查同意，伦理委员会办公室电话 0591-87981029 。如果您同意加入此项研究，请您仔细阅读，如有任何疑问请向负责该项研究的研究者提出。

一、研究目的：

我们课题组在前面研究过程中发现，TRAF6 能够通过调控 NF-κB 和 AP-1 信号通路促进结直肠癌的进展。研究表明 TRAF6 能够调控肿瘤进展中的多个信号通路，处于肿瘤信号调控的核心位置，是肿瘤进展的关键限速节点。但是，TRAF6 能否程序性坏死通路影响结直肠癌的坏死，迄今未知。因此，本项目首先探讨 TRAF6 对结直肠癌坏死的影响；随后，进一步研究 TRAF6 是否通过 RIPK1-RIPK3-MLKL 信号通路调控结直肠癌的坏死；最后更加深入的研究 TRAF6 调控 RIPK1-RIPK3-MLKL 信号通路的确切机制。研究结果将进一步的揭示结直肠癌发展的机制，为抗结直肠癌复发转移理论研究和靶向药物设计提供新的思路。

二、研究过程和方法：

如果您同意参与这项研究，我们将对每位受试者进行编号，建立研究档案。在研究的过程中我们需要采集您手术后的组织标本，由专业人员在您手术后当日，选取手术后标本的癌组织和癌旁组织，大小各约 0.8cm*0.8cm。您的所有采集样本仅用于"TRAF6 调控 CREB/RIPK1-RIPK3-MLKL 信号通路抑制程序性坏死影响结直肠癌进展的机制研究"该实验的研究。如果您同意参与这项研究，我们将对每位受试者进行编号，建立研究档案。

三、研究可能的受益：

参与本研究将为结直肠癌疾病的研究提供有益的信息。

四、研究风险与不适：

本研究您无需承担任何费用及压力，也不会给您造成损伤。本研究将收集您的临床病理信息和剩余结直肠癌手术的组织标本，不会对您产生常规诊疗之外的风险与不适。您的血样品收集由专业人员严格按照无菌要求操作，采集过程可能出现一些并发症，包括局部血肿、疼痛、过敏以及少数人晕血晕针等。

五、隐私问题：

如果您决定参加本项研究，您参加试验及在试验中的个人资料均属保密。对于您来说，所有的信息将是保密的。您的标本将以研究编号数字而非您的姓名加以标识。可以识别您身份的信息将不会透露给研究小组以外的成员，除非获得您的许可。所有的研究成员和研究申办方都被要求对您的身份保密。您的档案将保存在福建医科大学附属第一医院 16 区胃肠外科档案柜中，仅供研究人员查阅。为确保研究按照规定进行，必要时，政府管理部门或伦理审查委员会的成员按规定可以在研究单位查阅您的个人资料。这项研究结果发表时，将不会披露您个人的任何资料。

六、费用：

参与本研究无常规诊疗之外费用。

七、补偿：

参与该研究无干预，没有风险和补偿。

八、自由退出：

作为受试者，您可随时了解与本研究有关的信息资料和研究进展，自愿决定（继续）参加还是不（继续）参加。参加后，无论是否发生伤害，您可以选择在任何时候通知研究者要求退出研究，您的数据将不纳入研究结果，您的任何医疗待遇与权益不会因此而受到影响。在试验过程中，若会对您造成严重的伤害，研究者也将会中止研究的进行。

但在参加研究期间，请您提供有关自身病史和当前身体状况的真实情况；告诉研究医生自己在本次研究期间所出现的任何不适；不得服用受限制的药物、食物等；告诉研究医生自己在最近是否曾参与其他研究，或目前正参与其他研究。如果因为您没有遵守研究计划，或者发生了与研究相关的损伤或者有任何其它原因，研究医师可以终止您继续参与本项研究。

九、联系方式：

如果您有与本研究有关的问题，或您在研究过程中发生了任何不适与损伤，或有关于本项研究参加者权益方面的问题，您可以与朱广伟医生联系，电话 18060473703。

十、知情同意签字：

我已经阅读了本知情同意书，并且我的医生 （签字）已经将此次临床试验的目的、内容、风险和受益情况向我作了详细的解释说明，对我询问的所有问题也给予了解答，我对此项临床研究已经了解，我自愿参加本项研究。

受试者签名：唐龙光
联系手机号：15980121159
日期：2018 年 5 月 11 日

研究者签字：朱广伟
联系手机号：18060473703
日期：2018 年 5 月 11 日

（如果受试者不识字时尚需见证人签名，如果受试者无行为能力时则需代理人同意）

法定代理人/ 见证人签名：

与受试者的关系：

日期：_____年_____月_____日

Patient T5

临床研究知情同意书

尊敬的受试者：

您将被邀请参加一项由福建医科大学附属第一医院朱广伟（项目负责人）（PI 电话号码：18060473703）主持的研究，研究项目名称：TRAF6 调控 CREB/RIPK1-RIPK3-MLKL 信号通路抑制程序性坏死影响结直肠癌进展的机制研究。您参加本项研究是自愿的，本知情同意书提供给您一些信息以帮助您决定是否参加此项临床研究。本研究已通过福建医科大学附属第一医院伦理委员会审查同意，伦理委员会办公室电话 0591-87981029 。如果您同意加入此项研究，请您仔细阅读，如有任何疑问请向负责该项研究的研究者提出。

一、研究目的：

我们课题组在前面研究过程中发现，TRAF6 能够通过调控 NF-κB 和 AP-1 信号通路促进结直肠癌的进展。研究表明 TRAF6 能够调控肿瘤进展中的多个信号通路，处于肿瘤信号调控的核心位置，是肿瘤进展的关键限速节点。但是，TRAF6 能否程序性坏死通路影响结直肠癌的坏死，迄今未知。因此，本项目首先探讨 TRAF6 对结直肠癌坏死的影响；随后，进一步研究 TRAF6 是否通过 RIPK1-RIPK3-MLKL 信号通路调控结直肠癌的坏死；最后更加深入的研究 TRAF6 调控 RIPK1-RIPK3-MLKL 信号通路的确切机制。研究结果将进一步的揭示结直肠癌发展的机制，为抗结直肠癌复发转移理论研究和靶向药物设计提供新的思路。

二、研究过程和方法：

如果您同意参与这项研究，我们将对每位受试者进行编号，建立研究档案。在研究的过程中我们需要采集您手术后的组织标本，由专业人员在您手术后当日，选取手术后标本的癌组织和癌旁组织，大小各约 0.8cm*0.8cm。您的所有采集样本仅用于"TRAF6 调控 CREB/RIPK1-RIPK3-MLKL 信号通路抑制程序性坏死影响结直肠癌进展的机制研究"该实验的研究。如果您同意参与这项研究，我们将对每位受试者进行编号，建立研究档案。

三、研究可能的受益：

参与本研究将为结直肠癌疾病的研究提供有益的信息。

四、研究风险与不适：

本研究您无需承担任何费用及压力，也不会给您造成损伤。本研究将收集您的临床病理信息和剩余结直肠癌手术的组织标本，不会对您产生常规诊疗之外的风险与不适。您的血样品收集由专业人员严格按照无菌要求操作，采集过程可能出现一些并发症，包括局部血肿、疼痛、过敏以及少数人晕血晕针等。

五、隐私问题：

如果您决定参加本项研究，您参加试验及在试验中的个人资料均属保密。对于您来说，所有的信息将是保密的。您的标本将以研究编号数字而非您的姓名加以标识。可以识别您身份的信息将不会透露给研究小组以外的成员，除非获得您的许可。所有的研究成员和研究申办方都被要求对您的身份保密。您的档案将保存在福建医科大学附属第一医院 16 区胃肠外科档案柜中，仅供研究人员查阅。为确保研究按照规定进行，必要时，政府管理部门或伦理审查委员会的成员按规定可以在研究单位查阅您的个人资料。这项研究结果发表时，将不会披露您个人的任何资料。

六、费用：

参与本研究无常规诊疗之外费用。

七、补偿：

参与该研究无干预，没有风险和补偿。

八、自由退出：

作为受试者，您可随时了解与本研究有关的信息资料和研究进展，自愿决定（继续）参加还是不（继续）参加。参加后，无论是否发生伤害，您可以选择在任何时候通知研究者要求退出研究，您的数据将不纳入研究结果，您的任何医疗待遇与权益不会因此而受到影响。在试验过程中，若会对您造成严重的伤害，研究者也将会中止研究的进行。

但在参加研究期间，请您提供有关自身病史和当前身体状况的真实情况；告诉研究医生自己在本次研究期间所出现的任何不适；不得服用受限制的药物、食物等；告诉研究医生自己在最近是否曾参与其他研究，或目前正参与其他研究。如果因为您没有遵守研究计划，或者发生了与研究相关的损伤或者有任何其它原因，研究医师可以终止您继续参与本项研究。

九、联系方式：

如果您有与本研究有关的问题，或您在研究过程中发生了任何不适与损伤，或有关于本项研究参加者权益方面的问题，您可以与朱广伟医生联系，电话 18060473703。

十、知情同意签字：

我已经阅读了本知情同意书，并且我的医生 （签字）已经将此次临床试验的目的、内容、风险和受益情况向我作了详细的解释说明，对我询问的所有问题也给予了解答，我对此项临床研究已经了解，我自愿参加本项研究。

受试者签名：邹雅红　　　　研究者签字：朱广伟

联系手机号：13805012196　　　　联系手机号：18060473703

日期：2018 年 5 月 14 日　　　　日期：2018 年 5 月 14 日

（如果受试者不识字时尚需见证人签名，如果受试者无行为能力时则需代理人同意）

法定代理人/ 见证人签名：

与受试者的关系：

日期：______年______月______日

Patient T6

临床研究知情同意书

尊敬的受试者：

您将被邀请参加一项由福建医科大学附属第一医院朱广伟（项目负责人）（PI 电话号码：18060473703）主持的研究，研究项目名称：TRAF6 调控 CREB/RIPK1-RIPK3-MLKL 信号通路抑制程序性坏死影响结直肠癌进展的机制研究。您参加本项研究是自愿的，本知情同意书提供给您一些信息以帮助您决定是否参加此项临床研究。本研究已通过福建医科大学附属第一医院伦理委员会审查同意，伦理委员会办公室电话 0591-87981029 。如果您同意加入此项研究，请您仔细阅读，如有任何疑问请向负责该项研究的研究者提出。

一、研究目的：

我们课题组在前面研究过程中发现，TRAF6 能够通过调控 NF-κB 和 AP-1 信号通路促进结直肠癌的进展。研究表明 TRAF6 能够调控肿瘤进展中的多个信号通路，处于肿瘤信号调控的核心位置，是肿瘤进展的关键限速节点。但是，TRAF6 能否程序性坏死通路影响结直肠癌的坏死，迄今未知。因此，本项目首先探讨 TRAF6 对结直肠癌坏死的影响；随后，进一步研究 TRAF6 是否通过 RIPK1-RIPK3-MLKL 信号通路调控结直肠癌的坏死；最后更加深入的研究 TRAF6 调控 RIPK1-RIPK3-MLKL 信号通路的确切机制。研究结果将进一步的揭示结直肠癌发展的机制，为抗结直肠癌复发转移理论研究和靶向药物设计提供新的思路。

二、研究过程和方法：

如果您同意参与这项研究，我们将对每位受试者进行编号，建立研究档案。在研究的过程中我们需要采集您手术后的组织标本，由专业人员在您手术后当日，选取手术后标本的癌组织和癌旁组织，大小各约 0.8cm*0.8cm。您的所有采集样本仅用于"TRAF6 调控 CREB/RIPK1-RIPK3-MLKL 信号通路抑制程序性坏死影响结直肠癌进展的机制研究"该实验的研究。如果您同意参与这项研究，我们将对每位受试者进行编号，建立研究档案。

三、研究可能的受益：

参与本研究将为结直肠癌疾病的研究提供有益的信息。

四、研究风险与不适：

本研究您无需承担任何费用及压力，也不会给您造成损伤。本研究将收集您的临床病理信息和剩余结直肠癌手术的组织标本，不会对您产生常规诊疗之外的风险与不适。您的血样品收集由专业人员严格按照无菌要求操作，采集过程可能出现一些并发症，包括局部血肿、疼痛、过敏以及少数人晕血晕针等。

五、隐私问题：

如果您决定参加本项研究，您参加试验及在试验中的个人资料均属保密。对于您来说，所有的信息将是保密的。您的标本将以研究编号数字而非您的姓名加以标识。可以识别您身份的信息将不会透露给研究小组以外的成员，除非获得您的许可。所有的研究成员和研究申办方都被要求对您的身份保密。您的档案将保存在福建医科大学附属第一医院 16 区胃肠外科档案柜中，仅供研究人员查阅。为确保研究按照规定进行，必要时，政府管理部门或伦理审查委员会的成员按规定可以在研究单位查阅您的个人资料。这项研究结果发表时，将不会披露您个人的任何资料。

六、费用：

参与本研究无常规诊疗之外费用。

七、补偿：

参与该研究无干预，没有风险和补偿。

八、自由退出：

作为受试者，您可随时了解与本研究有关的信息资料和研究进展，自愿决定（继续）参加还是不（继续）参加。参加后，无论是否发生伤害，您可以选择在任何时候通知研究者要求退出研究，您的数据将不纳入研究结果，您的任何医疗待遇与权益不会因此而受到影响。在试验过程中，若会对您造成严重的伤害，研究者也将会中止研究的进行。

但在参加研究期间，请您提供有关自身病史和当前身体状况的真实情况；告诉研究医生自己在本次研究期间所出现的任何不适；不得服用受限制的药物、食物等；告诉研究医生自己在最近是否曾参与其他研究，或目前正参与其他研究。如果因为您没有遵守研究计划，或者发生了与研究相关的损伤或者有任何其它原因，研究医师可以终止您继续参与本项研究。

九、联系方式：

如果您有与本研究有关的问题，或您在研究过程中发生了任何不适与损伤，或有关于本项研究参加者权益方面的问题，您可以与朱广伟医生联系，电话 18060473703。

十、知情同意签字：

我已经阅读了本知情同意书，并且我的医生 （签字）已经将此次临床试验的目的、内容、风险和受益情况向我作了详细的解释说明，对我询问的所有问题也给予了解答，我对此项临床研究已经了解，我自愿参加本项研究。

受试者签名：林其和
联系手机号：13960773498
日期：2018 年 5 月 16 日

研究者签字：朱广伟
联系手机号：18060473703
日期：2018 年 5 月 16 日

（如果受试者不识字时尚需见证人签名，如果受试者无行为能力时则需代理人同意）

法定代理人/ 见证人签名：

与受试者的关系：

日期：_____年_____月_____日

Patient T7

临床研究知情同意书

尊敬的受试者：

您将被邀请参加一项由福建医科大学附属第一医院朱广伟（项目负责人）（PI 电话号码：18060473703）主持的研究，研究项目名称：TRAF6 调控 CREB/RIPK1-RIPK3-MLKL 信号通路抑制程序性坏死影响结直肠癌进展的机制研究。您参加本项研究是自愿的，本知情同意书提供给您一些信息以帮助您决定是否参加此项临床研究。本研究已通过福建医科大学附属第一医院伦理委员会审查同意，伦理委员会办公室电话 0591-87981029 。如果您同意加入此项研究，请您仔细阅读，如有任何疑问请向负责该项研究的研究者提出。

一、研究目的：

我们课题组在前面研究过程中发现，TRAF6 能够通过调控 NF-κB 和 AP-1 信号通路促进结直肠癌的进展。研究表明 TRAF6 能够调控肿瘤进展中的多个信号通路，处于肿瘤信号调控的核心位置，是肿瘤进展的关键限速节点。但是，TRAF6 能否程序性坏死通路影响结直肠癌的坏死，迄今未知。因此，本项目首先探讨 TRAF6 对结直肠癌坏死的影响；随后，进一步研究 TRAF6 是否通过 RIPK1-RIPK3-MLKL 信号通路调控结直肠癌的坏死；最后更加深入的研究 TRAF6 调控 RIPK1-RIPK3-MLKL 信号通路的确切机制。研究结果将进一步的揭示结直肠癌发展的机制，为抗结直肠癌复发转移理论研究和靶向药物设计提供新的思路。

二、研究过程和方法：

如果您同意参与这项研究，我们将对每位受试者进行编号，建立研究档案。在研究的过程中我们需要采集您手术后的组织标本，由专业人员在您手术后当日，选取手术后标本的癌组织和癌旁组织，大小各约 0.8cm*0.8cm。您的所有采集样本仅用于“TRAF6 调控 CREB/RIPK1-RIPK3-MLKL 信号通路抑制程序性坏死影响结直肠癌进展的机制研究”该实验的研究。如果您同意参与这项研究，我们将对每位受试者进行编号，建立研究档案。

三、研究可能的受益：

参与本研究将为结直肠癌疾病的研究提供有益的信息。

四、研究风险与不适：

本研究您无需承担任何费用及压力，也不会给您造成损伤。本研究将收集您的临床病理信息和剩余结直肠癌手术的组织标本，不会对您产生常规诊疗之外的风险与不适。您的血样品收集由专业人员严格按照无菌要求操作，采集过程可能出现一些并发症，包括局部血肿、疼痛、过敏以及少数人晕血晕针等。

五、隐私问题：

如果您决定参加本项研究，您参加试验及在试验中的个人资料均属保密。对于您来说，所有的信息将是保密的。您的标本将以研究编号数字而非您的姓名加以标识。可以识别您身份的信息将不会透露给研究小组以外的成员，除非获得您的许可。所有的研究成员和研究申办方都被要求对您的身份保密。您的档案将保存在福建医科大学附属第一医院 16 区胃肠外科档案柜中，仅供研究人员查阅。为确保研究按照规定进行，必要时，政府管理部门或伦理审查委员会的成员按规定可以在研究单位查阅您的个人资料。这项研究结果发表时，将不会披露您个人的任何资料。

六、费用：

参与本研究无常规诊疗之外费用。

七、补偿：

参与该研究无干预，没有风险和补偿。

八、自由退出：

作为受试者，您可随时了解与本研究有关的信息资料和研究进展，自愿决定（继续）参加还是不（继续）参加。参加后，无论是否发生伤害，您可以选择在任何时候通知研究者要求退出研究，您的数据将不纳入研究结果，您的任何医疗待遇与权益不会因此而受到影响。在试验过程中，若会对您造成严重的伤害，研究者也将会中止研究的进行。

但在参加研究期间，请您提供有关自身病史和当前身体状况的真实情况；告诉研究医生自己在本次研究期间所出现的任何不适；不得服用受限制的药物、食物等；告诉研究医生自己在最近是否曾参与其他研究，或目前正参与其他研究。如果因为您没有遵守研究计划，或者发生了与研究相关的损伤或者有任何其它原因，研究医师可以终止您继续参与本项研究。

九、联系方式：

如果您有与本研究有关的问题，或您在研究过程中发生了任何不适与损伤，或有关于本项研究参加者权益方面的问题，您可以与朱广伟医生联系，电话 18060473703。

十、知情同意签字：

我已经阅读了本知情同意书，并且我的医生 （签字）已经将此次临床试验的目的、内容、风险和受益情况向我作了详细的解释说明，对我询问的所有问题也给予了解答，我对此项临床研究已经了解，我自愿参加本项研究。

受试者签名：施能炎
联系手机号：15980033667
日期：2018年 5 月 15 日

研究者签字：朱广伟
联系手机号：18060473703
日期：2018年5月15日

（如果受试者不识字时尚需见证人签名，如果受试者无行为能力时则需代理人同意）

法定代理人/ 见证人签名：

与受试者的关系：

日期：_____年_____月_____日

Patient T8

临床研究知情同意书

尊敬的受试者：

您将被邀请参加一项由福建医科大学附属第一医院朱广伟（项目负责人）(PI 电话号码：18060473703）主持的研究，研究项目名称：TRAF6 调控 CREB/RIPK1-RIPK3-MLKL 信号通路抑制程序性坏死影响结直肠癌进展的机制研究。您参加本项研究是自愿的，本知情同意书提供给您一些信息以帮助您决定是否参加此项临床研究。本研究已通过福建医科大学附属第一医院伦理委员会审查同意，伦理委员会办公室电话 0591-87981029 。如果您同意加入此项研究，请您仔细阅读，如有任何疑问请向负责该项研究的研究者提出。

一、研究目的：

我们课题组在前面研究过程中发现，TRAF6 能够通过调控 NF-κB 和 AP-1 信号通路促进结直肠癌的进展。研究表明 TRAF6 能够调控肿瘤进展中的多个信号通路，处于肿瘤信号调控的核心位置，是肿瘤进展的关键限速节点。但是，TRAF6 能否程序性坏死通路影响结直肠癌的坏死，迄今未知。因此，本项目首先探讨 TRAF6 对结直肠癌坏死的影响；随后，进一步研究 TRAF6 是否通过 RIPK1-RIPK3-MLKL 信号通路调控结直肠癌的坏死；最后更加深入的研究 TRAF6 调控 RIPK1-RIPK3-MLKL 信号通路的确切机制。研究结果将进一步的揭示结直肠癌发展的机制，为抗结直肠癌复发转移理论研究和靶向药物设计提供新的思路。

二、研究过程和方法：

如果您同意参与这项研究，我们将对每位受试者进行编号，建立研究档案。在研究的过程中我们需要采集您手术后的组织标本，由专业人员在您手术后当日，选取手术后标本的癌组织和癌旁组织，大小各约 0.8cm*0.8cm。您的所有采集样本仅用于"TRAF6 调控 CREB/RIPK1-RIPK3-MLKL 信号通路抑制程序性坏死影响结直肠癌进展的机制研究"该实验的研究。如果您同意参与这项研究，我们将对每位受试者进行编号，建立研究档案。

三、研究可能的受益：

参与本研究将为结直肠癌疾病的研究提供有益的信息。

四、研究风险与不适：

本研究您无需承担任何费用及压力，也不会给您造成损伤。本研究将收集您的临床病理信息和剩余结直肠癌手术的组织标本，不会对您产生常规诊疗之外的风险与不适。您的血样品收集由专业人员严格按照无菌要求操作，采集过程可能出现一些并发症，包括局部血肿、疼痛、过敏以及少数人晕血晕针等。

五、隐私问题：

如果您决定参加本项研究，您参加试验及在试验中的个人资料均属保密。对于您来说，所有的信息将是保密的。您的标本将以研究编号数字而非您的姓名加以标识。可以识别您身份的信息将不会透露给研究小组以外的成员，除非获得您的许可。所有的研究成员和研究申办方都被要求对您的身份保密。您的档案将保存在福建医科大学附属第一医院 16 区胃肠外科档案柜中，仅供研究人员查阅。为确保研究按照规定进行，必要时，政府管理部门或伦理审查委员会的成员按规定可以在研究单位查阅您的个人资料。这项研究结果发表时，将不会披露您个人的任何资料。

六、费用：

参与本研究无常规诊疗之外费用。

七、补偿：

参与该研究无干预，没有风险和补偿。

八、自由退出：

作为受试者，您可随时了解与本研究有关的信息资料和研究进展，自愿决定（继续）参加还是不（继续）参加。参加后，无论是否发生伤害，您可以选择在任何时候通知研究者要求退出研究，您的数据将不纳入研究结果，您的任何医疗待遇与权益不会因此而受到影响。在试验过程中，若会对您造成严重的伤害，研究者也将会中止研究的进行。

但在参加研究期间，请您提供有关自身病史和当前身体状况的真实情况；告诉研究医生自己在本次研究期间所出现的任何不适；不得服用受限制的药物、食物等；告诉研究医生自己在最近是否曾参与其他研究，或目前正参与其他研究。如果因为您没有遵守研究计划，或者发生了与研究相关的损伤或者有任何其它原因，研究医师可以终止您继续参与本项研究。

九、联系方式：

如果您有与本研究有关的问题，或您在研究过程中发生了任何不适与损伤，或有关于本项研究参加者权益方面的问题，您可以与朱广伟医生联系，电话 18060473703。

十、知情同意签字：

我已经阅读了本知情同意书，并且我的医生 （签字）已经将此次临床试验的目的、内容、风险和受益情况向我作了详细的解释说明，对我询问的所有问题也给予了解答，我对此项临床研究已经了解，我自愿参加本项研究。

受试者签名：黄文超
联系手机号：13505921653
日期：2018 年 5 月 16 日

研究者签字：朱广伟
联系手机号：18060473703
日期：2018 年 5 月 16 日

（如果受试者不识字时尚需见证人签名，如果受试者无行为能力时则需代理人同意）

法定代理人/ 见证人签名：

与受试者的关系：

日期：_____年_______月_______日

Patient T9

临床研究知情同意书

尊敬的受试者：

您将被邀请参加一项由福建医科大学附属第一医院朱广伟（项目负责人）（PI 电话号码：18060473703）主持的研究，研究项目名称：TRAF6 调控 CREB/RIPK1-RIPK3-MLKL 信号通路抑制程序性坏死影响结直肠癌进展的机制研究。您参加本项研究是自愿的，本知情同意书提供给您一些信息以帮助您决定是否参加此项临床研究。本研究已通过福建医科大学附属第一医院伦理委员会审查同意，伦理委员会办公室电话 0591-87981029 。如果您同意加入此项研究，请您仔细阅读，如有任何疑问请向负责该项研究的研究者提出。

一、研究目的：

我们课题组在前面研究过程中发现，TRAF6 能够通过调控 NF-κB 和 AP-1 信号通路促进结直肠癌的进展。研究表明 TRAF6 能够调控肿瘤进展中的多个信号通路，处于肿瘤信号调控的核心位置，是肿瘤进展的关键限速节点。但是，TRAF6 能否程序性坏死通路影响结直肠癌的坏死，迄今未知。因此，本项目首先探讨 TRAF6 对结直肠癌坏死的影响；随后，进一步研究 TRAF6 是否通过 RIPK1-RIPK3-MLKL 信号通路调控结直肠癌的坏死；最后更加深入的研究 TRAF6 调控 RIPK1-RIPK3-MLKL 信号通路的确切机制。研究结果将进一步的揭示结直肠癌发展的机制，为抗结直肠癌复发转移理论研究和靶向药物设计提供新的思路。

二、研究过程和方法：

如果您同意参与这项研究，我们将对每位受试者进行编号，建立研究档案。在研究的过程中我们需要采集您手术后的组织标本，由专业人员在您手术后当日，选取手术后标本的癌组织和癌旁组织，大小各约 0.8cm*0.8cm。您的所有采集样本仅用于"TRAF6 调控 CREB/RIPK1-RIPK3-MLKL 信号通路抑制程序性坏死影响结直肠癌进展的机制研究"该实验的研究。如果您同意参与这项研究，我们将对每位受试者进行编号，建立研究档案。

三、研究可能的受益：

参与本研究将为结直肠癌疾病的研究提供有益的信息。

四、研究风险与不适：

本研究您无需承担任何费用及压力，也不会给您造成损伤。本研究将收集您的临床病理信息和剩余结直肠癌手术的组织标本，不会对您产生常规诊疗之外的风险与不适。您的血样品收集由专业人员严格按照无菌要求操作，采集过程可能出现一些并发症，包括局部血肿、疼痛、过敏以及少数人晕血晕针等。

五、隐私问题：

如果您决定参加本项研究，您参加试验及在试验中的个人资料均属保密。对于您来说，所有的信息将是保密的。您的标本将以研究编号数字而非您的姓名加以标识。可以识别您身份的信息将不会透露给研究小组以外的成员，除非获得您的许可。所有的研究成员和研究申办方都被要求对您的身份保密。您的档案将保存在福建医科大学附属第一医院 16 区胃肠外科档案柜中，仅供研究人员查阅。为确保研究按照规定进行，必要时，政府管理部门或伦理审查委员会的成员按规定可以在研究单位查阅您的个人资料。这项研究结果发表时，将不会披露您个人的任何资料。

六、费用：

参与本研究无常规诊疗之外费用。

七、补偿：

参与该研究无干预，没有风险和补偿。

八、自由退出：

作为受试者，您可随时了解与本研究有关的信息资料和研究进展，自愿决定（继续）参加还是不（继续）参加。参加后，无论是否发生伤害，您可以选择在任何时候通知研究者要求退出研究，您的数据将不纳入研究结果，您的任何医疗待遇与权益不会因此而受到影响。在试验过程中，若会对您造成严重的伤害，研究者也将会中止研究的进行。

但在参加研究期间，请您提供有关自身病史和当前身体状况的真实情况；告诉研究医生自己在本次研究期间所出现的任何不适；不得服用受限制的药物、食物等；告诉研究医生自己在最近是否曾参与其他研究，或目前正参与其他研究。如果因为您没有遵守研究计划，或者发生了与研究相关的损伤或者有任何其它原因，研究医师可以终止您继续参与本项研究。

九、联系方式：

如果您有与本研究有关的问题，或您在研究过程中发生了任何不适与损伤，或有关于本项研究参加者权益方面的问题，您可以与朱广伟医生联系，电话 18060473703。

十、知情同意签字：

我已经阅读了本知情同意书，并且我的医生 （签字）已经将此次临床试验的目的、内容、风险和受益情况向我作了详细的解释说明，对我询问的所有问题也给予了解答，我对此项临床研究已经了解，我自愿参加本项研究。

受试者签名：潘环英
联系手机号：13489888551
日期：2018 年 5 月 7 日

研究者签字：朱广伟
联系手机号：18060473703
日期：2018 年 5 月 7 日

（如果受试者不识字时尚需见证人签名，如果受试者无行为能力时则需代理人同意）
法定代理人/ 见证人签名：
与受试者的关系：

日期：_____ 年_____ 月_____ 日

Patient T10

临床研究知情同意书

尊敬的受试者：

您将被邀请参加一项由福建医科大学附属第一医院朱广伟（项目负责人）（PI 电话号码：18060473703）主持的研究，研究项目名称：TRAF6 调控 CREB/RIPK1-RIPK3-MLKL 信号通路抑制程序性坏死影响结直肠癌进展的机制研究。您参加本项研究是自愿的，本知情同意书提供给您一些信息以帮助您决定是否参加此项临床研究。本研究已通过福建医科大学附属第一医院伦理委员会审查同意，伦理委员会办公室电话 0591-87981029 。如果您同意加入此项研究，请您仔细阅读，如有任何疑问请向负责该项研究的研究者提出。

一、研究目的：

我们课题组在前面研究过程中发现，TRAF6 能够通过调控 NF-κB 和 AP-1 信号通路促进结直肠癌的进展。研究表明 TRAF6 能够调控肿瘤进展中的多个信号通路，处于肿瘤信号调控的核心位置，是肿瘤进展的关键限速节点。但是，TRAF6 能否程序性坏死通路影响结直肠癌的坏死，迄今未知。因此，本项目首先探讨 TRAF6 对结直肠癌坏死的影响；随后，进一步研究 TRAF6 是否通过 RIPK1-RIPK3-MLKL 信号通路调控结直肠癌的坏死；最后更加深入的研究 TRAF6 调控 RIPK1-RIPK3-MLKL 信号通路的确切机制。研究结果将进一步的揭示结直肠癌发展的机制，为抗结直肠癌复发转移理论研究和靶向药物设计提供新的思路。

二、研究过程和方法：

如果您同意参与这项研究，我们将对每位受试者进行编号，建立研究档案。在研究的过程中我们需要采集您手术后的组织标本，由专业人员在您手术后当日，选取手术后标本的癌组织和癌旁组织，大小各约 0.8cm*0.8cm。您的所有采集样本仅用于"TRAF6 调控 CREB/RIPK1-RIPK3-MLKL 信号通路抑制程序性坏死影响结直肠癌进展的机制研究"该实验的研究。如果您同意参与这项研究，我们将对每位受试者进行编号，建立研究档案。

三、研究可能的受益：

参与本研究将为结直肠癌疾病的研究提供有益的信息。

四、研究风险与不适：

本研究您无需承担任何费用及压力，也不会给您造成损伤。本研究将收集您的临床病理信息和剩余结直肠癌手术的组织标本，不会对您产生常规诊疗之外的风险与不适。您的血样品收集由专业人员严格按照无菌要求操作，采集过程可能出现一些并发症，包括局部血肿、疼痛、过敏以及少数人晕血晕针等。

五、隐私问题：

如果您决定参加本项研究，您参加试验及在试验中的个人资料均属保密。对于您来说，所有的信息将是保密的。您的标本将以研究编号数字而非您的姓名加以标识。可以识别您身份的信息将不会透露给研究小组以外的成员，除非获得您的许可。所有的研究成员和研究申办方都被要求对您的身份保密。您的档案将保存在福建医科大学附属第一医院 16 区胃肠外科档案柜中，仅供研究人员查阅。为确保研究按照规定进行，必要时，政府管理部门或伦理审查委员会的成员按规定可以在研究单位查阅您的个人资料。这项研究结果发表时，将不会披露您个人的任何资料。

六、费用：

参与本研究无常规诊疗之外费用。

七、补偿：

参与该研究无干预，没有风险和补偿。

八、自由退出：

作为受试者，您可随时了解与本研究有关的信息资料和研究进展，自愿决定（继续）参加还是不（继续）参加。参加后，无论是否发生伤害，您可以选择在任何时候通知研究者要求退出研究，您的数据将不纳入研究结果，您的任何医疗待遇与权益不会因此而受到影响。在试验过程中，若会对您造成严重的伤害，研究者也将会中止研究的进行。

但在参加研究期间，请您提供有关自身病史和当前身体状况的真实情况；告诉研究医生自己在本次研究期间所出现的任何不适；不得服用受限制的药物、食物等；告诉研究医生自己在最近是否曾参与其他研究，或目前正参与其他研究。如果因为您没有遵守研究计划，或者发生了与研究相关的损伤或者有任何其它原因，研究医师可以终止您继续参与本项研究。

九、联系方式：

如果您有与本研究有关的问题，或您在研究过程中发生了任何不适与损伤，或有关于本项研究参加者权益方面的问题，您可以与朱广伟医生联系，电话 18060473703。

十、知情同意签字：

我已经阅读了本知情同意书，并且我的医生 （签字）已经将此次临床试验的目的、内容、风险和受益情况向我作了详细的解释说明，对我询问的所有问题也给予了解答，我对此项临床研究已经了解，我自愿参加本项研究。

受试者签名：伊柏林 研究者签字：朱广伟

联系手机号：13859400203 联系手机号：18060473703

日期：2018 年 5 月 15 日 日期：2018 年 5 月 15 日

（如果受试者不识字时尚需见证人签名，如果受试者无行为能力时则需代理人同意）

法定代理人/ 见证人签名：

与受试者的关系：

日期：_____年_____月_____日
